# A comprehensive analysis of common genetic variation in prolactin (PRL) and PRL receptor (PRLR) genes in relation to plasma prolactin levels and breast cancer risk: the Multiethnic Cohort

**DOI:** 10.1186/1471-2350-8-72

**Published:** 2007-12-01

**Authors:** Sulggi A Lee, Christopher A Haiman, Noel P Burtt, Loreall C Pooler, Iona Cheng, Laurence N Kolonel, Malcolm C Pike, David Altshuler, Joel N Hirschhorn, Brian E Henderson, Daniel O Stram

**Affiliations:** 1 Department of Preventive Medicine, University of Southern California Keck School of Medicine, Norris Comprehensive Cancer Center, Los Angeles, CA, USA; 2 Cancer Etiology Program, Cancer Research Center of Hawaii, University of Hawaii Honolulu, HI, USA; 3 Broad Institute, Massachusetts Institute of Technology, Cambridge, MA, USA; 4 Department of Medicine, Harvard Medical School, Boston, MA, USA; 5 Department of Molecular Biology and Diabetes Unit, Massachusetts General Hospital, Boston, MA, USA; 6 Department of Genetics, Harvard Medical School, Boston, MA, USA; 7 Division of Endocrinology, Children's Hospital and Department of Pediatrics, Boston, MA, USA; 8 Department of Epidemiology and Biostatistics and Institute of Human Genetics, University of California, San Francisco, San Francisco, CA, USA

## Abstract

**Background:**

Studies in animals and humans clearly indicate a role for prolactin (PRL) in breast epithelial proliferation, differentiation, and tumorigenesis. Prospective epidemiological studies have also shown that women with higher circulating PRL levels have an increase in risk of breast cancer, suggesting that variability in PRL may also be important in determining a woman's risk.

**Methods:**

We evaluated genetic variation in the PRL and PRL receptor (PRLR) genes as predictors of plasma PRL levels and breast cancer risk among African-American, Native Hawaiian, Japanese-American, Latina, and White women in the Multiethnic Cohort Study (MEC). We selected single nucleotide polymorphisms (SNPs) from both the public (dbSNP) and private (Celera) databases to construct high density SNP maps that included up to 20 kilobases (kb) upstream of the transcription initiation site and 10 kb downstream of the last exon of each gene, for a total coverage of 59 kb in PRL and 210 kb in PRLR. We genotyped 80 SNPs in PRL and 173 SNPs in PRLR in a multiethnic panel of 349 unaffected subjects to characterize linkage disequilibrium (LD) and haplotype patterns. We sequenced the coding regions of PRL and PRLR in 95 advanced breast cancer cases (19 of each racial/ethnic group) to uncover putative functional variation. A total of 33 and 60 haplotype "tag" SNPs (tagSNPs) that allowed for high predictability (R_h_^2 ^≥ 0.70) of the common haplotypes in PRL and PRLR, respectively, were then genotyped in a multiethnic breast cancer case-control study of 1,615 invasive breast cancer cases and 1,962 controls in the MEC. We also assessed the association of common genetic variation with circulating PRL levels in 362 postmenopausal controls without a history of hormone therapy use at blood draw. Because of the large number of comparisons being performed we used a relatively stringent type I error criteria (p < 0.0005) for evaluating the significance of any single association to correct for performing approximately 100 independent tests, close to the number of tagSNPs genotyped for both genes.

**Results:**

We observed no significant associations between PRL and PRLR haplotypes or individual SNPs in relation to breast cancer risk. A nominally significant association was noted between prolactin levels and a tagSNP (tagSNP 44, rs2244502) in intron 1 of PRL. This SNP showed approximately a 50% increase in levels between minor allele homozygotes vs. major allele homozygotes. However, this association was not significant (p = 0.002) using our type I error criteria to correct for multiple testing, nor was this SNP associated with breast cancer risk (p = 0.58).

**Conclusion:**

In this comprehensive analysis covering 59 kb of the PRL locus and 210 kb of the PRLR locus, we found no significant association between common variation in these candidate genes and breast cancer risk or plasma PRL levels. The LD characterization of PRL and PRLR in this multiethnic population provide a framework for studying these genes in relation to other disease outcomes that have been associated with PRL, as well as for larger studies of plasma PRL levels.

## Background

Prolactin (PRL) is an essential regulator of mammary development, acting synergistically with a wide variety of hormones during puberty and pregnancy [1,2]. Early studies in animals first demonstrated that prolactin could induce spontaneous mammary tumors [3-6]. Results from in vitro studies support the findings from animal studies and suggest that PRL stimulates proliferation, [7-10] increases cell motility and cytoskeleton alterations [11], and promotes angiogenesis [12] in human breast cells. Prolactin receptor (PRLR), found in both normal and malignant breast tissue, has been reported to be slightly more prevalent in malignant tissue [13]. Though early clinical studies of patients treated with bromocriptine, an inhibitor of pituitary PRL, found no association with breast cancer, recent evidence of autocrine/paracrine regulation [14,15] of PRL in extra-pituitary tissue provides further support for a possible role of PRL in tumorigenesis.

There are few prospective epidemiological studies evaluating plasma PRL levels and breast cancer risk. The largest prospective cohort study of postmenopausal women reported a 34% increase in risk of breast cancer when comparing top to bottom quartiles (> 12 vs. < 7.4 ng/mL) of PRL levels [16]; these findings were similar to results from an earlier study reporting a non-significant increase in risk of 1.34, based on a smaller sample size [17]. Two smaller studies of postmenopausal women also reported a positive association, but these were also non-significant [18,19]. Results from case-control studies [20-27] give conflicting results and are difficult to interpret due to the retrospective nature of blood collection. There have been limited prospective data on prolactin levels and breast cancer risk among premenopausal women [18,19,28] until recently; the Nurses' Health Study reported a non-significant 30% increase in breast cancer risk among premenopausal women when comparing top to bottom quartiles (> 17.6 vs. < 9.8 ng/mL) of PRL levels among 377 cases and 786 controls [29].

In humans, the PRL gene lies on chromosome 6 and is approximately 10 kilobases (kb) in length with five coding exons [30]. An additional non-coding first exon has been described that lies 5.8 kb upstream of the pituitary promoter site [31]. This distal promoter region has been associated with extra-pituitary expression of PRL, described in a variety of tissues including decidua, lymphocytes, and breast tissue. Depending on promoter usage, PRL mRNAs may differ slightly in length but encode the same mature polypeptide protein hormone [32].

The human PRLR gene is located on chromosome 5 and is approximately 180 kb in length and is originally described as having 10 exons, of which exons 3–10 are coding exons [33]. Recently, six alternative non-coding first exons have been described whose functions are unknown but have been found to be expressed in human ovary, testis, liver, breast tissue, and breast cells [34,35]. In addition, an exon 11 located 15 kb downstream of exon 10 has been reported; alternative splicing of exons 10 and 11 appear to produce novel short forms of the receptor that may be involved in distinct signaling pathways than the common long form [36,37].

Previous studies have demonstrated that genetic polymorphisms in candidate genes can lead to variations in plasma levels of encoded proteins [38,39]. In this study, we used a combination of approaches that included sequencing the coding regions to identify common missense variation, and haplotype-based analyses to characterize common patterns of genetic variation across each locus to test the hypothesis that genetic variations in PRL and PRLR are associated with plasma PRL levels and breast cancer risk. Tests of association were performed in a large case-control study of breast cancer among African-American (AA), Native Hawaiian (NH), Japanese-American (JA), Latina (LA), and White (WH) women in the prospective Multiethnic Cohort Study (MEC). To our knowledge, this is the first comprehensive study of common genetic variation in PRL and PRLR genes in relation to breast cancer risk and plasma PRL levels in a multiethnic population

## Results

### Characterization of Genetic Variation at PRL and PRLR loci

**Figure 1:**
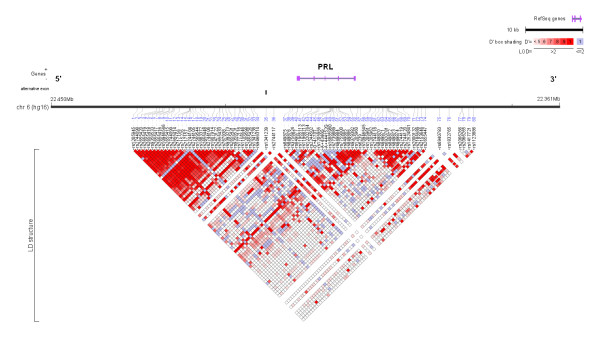
Linkage disequilibrium (LD) plot across the prolactin (PRL) locus for all racial/ethnic groups combined. The horizontal black line depicts the 59-kilobase region of chromosome (chr) 6 analyzed in our multiethnic panel. The PRL gene is shown in grey (RefSeq gene = completed genes from the human genome assembly). Alternative exon 1a (associated with the distal extra-pituitary promoter region) lies 5.8 kb upstream of exon 1 (associated with the pituitary promoter region). The 80 single nucleotide polymorphisms (SNPs) used for genetic characterization are listed below the black line. The LD plot, presented at the bottom of the figures, is based on the measure of *D*'. Each diamond indicates the pairwise magnitude of LD, with dark grey indicating strong LD (*D*' > 0.8) and a logarithm of odds score of greater than 2.0. (Figure prepared with LocusView, Broad Institute, Cambridge, MA, unpublished software by T. Petryshen, A. Kirby, and M. Ainscow [61]).

We genotyped 80 SNPs in PRL and 173 SNPs in PRLR (approximately 1 SNP every 1 kb) to characterize linkage disequilibrium (LD) and haplotype patterns in a multiethnic panel of 349 unaffected subjects (69–70 of each of the 5 racial/ethnic populations in the MEC). We characterized genetic variation across 59 kb of the PRL locus, 24 kb upstream of PRL's alternative first exon 1a (5.8 kb upstream of pituitary promoter site) to 20 kb downstream of exon 5, using 80 common (minor allele frequency, MAF, ≥ 5%) SNPs (Additional File 1, Table S1). In PRL, we observed three regions of LD (blocks 1, 3, 4, see the Methods section for a description of the criteria used to define LD block regions), and one 19 kb region ("pseudo-block 2") with little evidence of LD. Based on the dense coverage across this 19 kb region (1 common SNP every < 1 kb apart, on average), we decided to construct haplotypes to test associations with common variation (Figure 1, Additional File 1, Table S1). In this region, the multivariate squared correlation, *R*_s_^2^, between the selected tagSNPs and all SNPs examined in the multiethnic panel was = 0.70 in all ethnic groups, which suggests that unmeasured SNPs in this region are most likely well predicted by our set of tags. Thus, we describe four regions in PRL: block 1 (SNPs 1–24; 14 kb), "block" 2 (SNPs 25–45; 19 kb), block 3 (SNPs 46–59; 7 kb), and block 4 (SNPs 61–77; 14 kb). In general, block sizes in PRL were similar among racial/ethnic groups (Additional Files 2, 3, 4, 5, 6).

**Figure 2:**
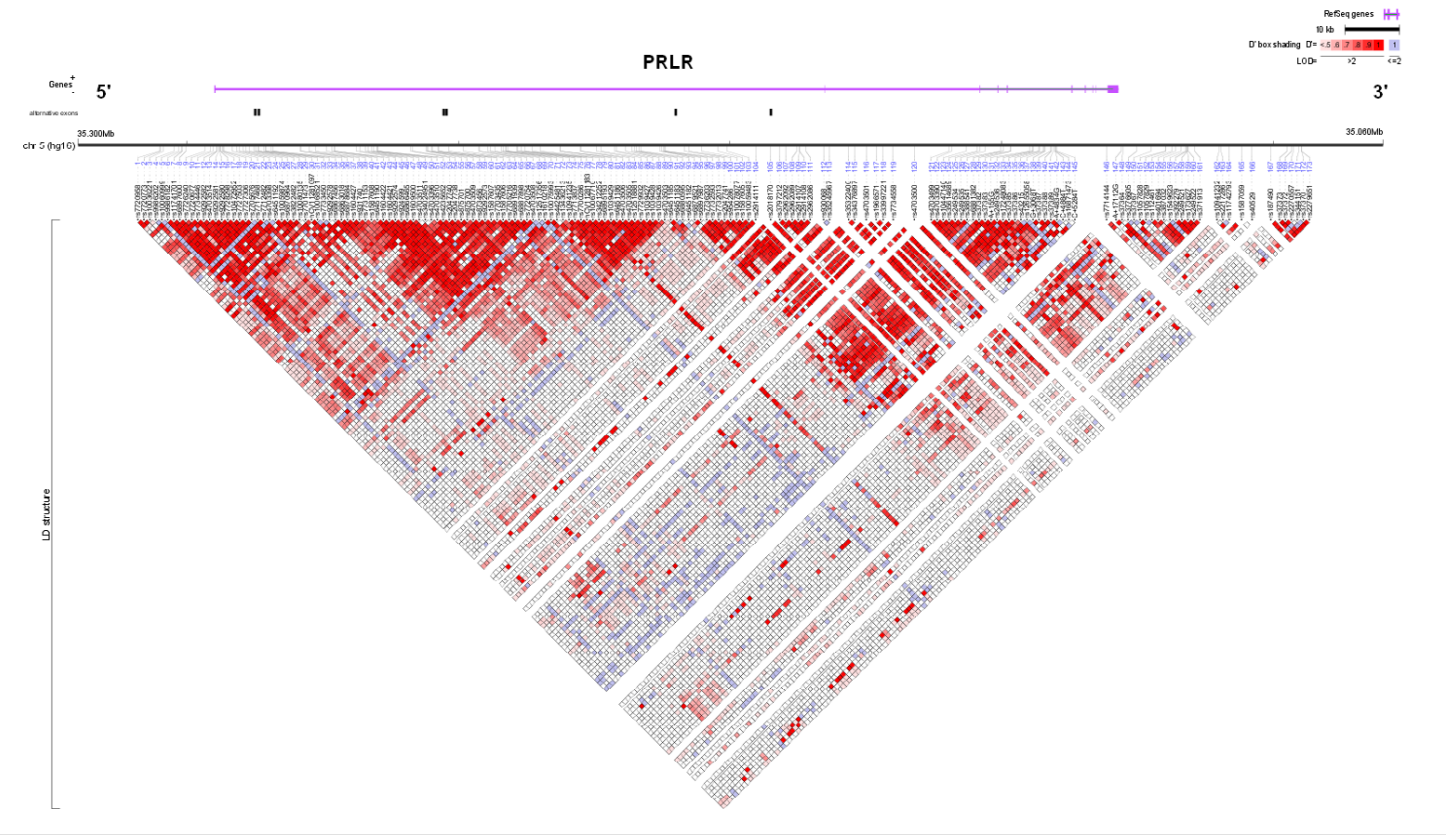
Linkage disequilibrium (LD) plot across the prolactin receptor (PRLR) locus for all racial/ethnic groups combined. The horizontal black line depicts the 210-kilobase region of chromosome (chr) 5 analyzed in our multiethnic panel. The PRLR gene is shown in grey (RefSeq gene = completed genes from the human genome assembly). Alternative first exons are shown in black below the gene: hE13, hE1N1, hE1N2, hE1N3, hE1N4, and hE1N5. The 173 single nucleotide polymorphisms (SNPs) used for genetic characterization are listed below the black line. The LD plot, presented at the bottom of the figures, is based on the measure of *D*'. Each diamond indicates the pairwise magnitude of LD, with dark grey indicating strong LD (*D*' > 0.8) and a logarithm of odds score of greater than 2.0. (Figure prepared with LocusView, Broad Institute, Cambridge, MA, unpublished software by T. Petryshen, A. Kirby, and M. Ainscow [61]).

In PRLR, we assessed 210 kb of the locus, from 25 kb upstream of the first alternative exon E13 to 10 kb downstream of exon 11 (the alternatively spliced exon 10) (Additional File 1, Table S2). Using 173 common SNPs, we defined nine blocks of LD in PRLR: block 1 (SNPs 6–30; 14 kb), block 2 (SNPs 31–39; 10 kb), block 3 (SNPs 41–66; 29 kb), block 4 (SNPs 73–88; 22 kb), block 5 (SNPs 95–113; 31 kb), block 6 (SNPs 114–135; 35 kb), block 7 (SNPs 136–153; 24 kb), block 8 (SNPs 154–161; 3 kb), and block 9 (SNPs 167–173; 6 kb) (Figure 2, Additional File 1, Table S2). Compared to the other racial/ethnic groups, African-Americans demonstrated smaller block sizes for block 3 (SNPs 49–58), block 5 (SNPs 102–113), block 6 (SNPs 114–124), and block 7 (SNPs 147–153), and Native Hawaiians had larger block sizes, with combined blocks 1–3 (SNPs 1–72) and blocks 5–9 (SNPs 97–173) (Additional Files 7, 8, 9, 10, 11). "Tagging" SNPs (tagSNPs) were selected to allow for high predictability of the common haplotypes (= 5% frequency in any one ethnic population) with LD blocks in both genes: 33 tagSNPs in PRL and 60 tagSNPs in PRLR (Additional File 1, Tables S1 and S2; see Methods for a description of the approach utilized to select tagSNPs). African-Americans demonstrated a greater number of common haplotypes per block (Additional File 1, Table S9). Therefore, in order to accurately predict the common haplotypes in PRLR for African-Americans, additional tagSNPs were genotyped for blocks 1, 2, 3, 5, 7, and 9 (tagSNPs 16, 24, 35, 49, 111, 112, 151, 153, 167, and 171).

Of the 60 tagSNPs selected in PRLR we were unable to genotype four of them in the case-control study because Illumina assays could not be designed, block 1: SNP6 (rs9986182), SNP12 (rs9292582), SNP24 (rs6451192), and SNP29 (rs7701473). This resulted in the inability to distinguish between haplotypes 1A1, 1A2, and 1A3 in LA (minor allele frequency 16.9%, 6.4%, and 6.6%), between haplotypes 1A1 and 1A3 in AA (9.2% and 2.2%), and between 1A1 and 1A2 in NH (17.2% and 4.5%) and in WH (34.6% and 5.9%) (Additional File 1, Table S9) which spans 14.2 kb, 142 kb upstream of the start codon in exon 3. Aside from block 1 of PRLR, the predicted common haplotypes frequencies in the multiethnic panel were similar to those observed in the larger case-control sample (Additional File 1, Tables S8-S11). Therefore, only haplotypes with ≥ 5% frequency in cases or controls, per each racial/ethnic group, are shown in Additional File 1, Tables S10 and S11. To assess how well the selected tagSNP perform in capturing the common SNPs that were not selected as tagSNPs in each population, we calculated multi-marker R^2 ^measures for both genes [40]. For PRL, the fraction of SNPs predicted with a multi-marker R^2 ^> 0.7 was 89%, 93%, 98%, 100%, and 100% for AA, NH, JA, LA, and WH, respectively. For PRLR (even without the four tagSNPs), the fraction of SNPs captured with multi-marker R^2 ^> 0.7 was 84%, 92%, 90%, 92%, and 93%. Thus, the selected tagSNPs capture most of the SNPs evaluated in the LD characterization phase, and based on high-density SNPs coverage in this study (1 SNPs every ~1 kb, on average), we expect these tags to also predict the vast majority of all common alleles in these genes.

We sequenced the exons and splice-site regions of PRL and PRLR in germline DNA from 95 advanced breast cancer cases (19 of each racial/ethnic group). PRL and PRLR sequencing confirmed only one missense SNP, Ile^100^Val (rs16871473) in exon 5 of PRLR. The SNP was observed most commonly among Native Hawaiians (MAFs, 11%, 15%, 5%, 1%, and 2% in AA, NH, JA, LA, and WH, respectively) (Additional File 1, Table S2). A previously reported missense SNP in exon 6 of PRLR (Ile^170^Leu) was monomorphic in all ethnic groups [41]. For PRL, we discovered a low frequency synonymous SNP in exon 3 (A+444152G). We were also able to validate a previously reported synonymous SNP in exon 5 (rs6239), but not a synonymous SNP in exon 2 (rs6240) or a missense SNP in exon 4 (rs6238) (Additional File 1, Table S1).

### Case-control analysis

The distribution of breast cancer risk factors among the 1,615 breast cancer cases and 1,962 controls were consistent with the patterns observed in the overall cohort, and have been previously published [42] (Additional File 1, Table S3). We tested the independent effects of each tagSNP for PRL and PRLR in the case-control population (Additional File 1, Tables S4 and S5). Odds ratios (ORs) and 95% confidence intervals (CIs) were estimated for each tagSNP using unconditional logistic regression adjusted for age and ethnicity (co-dominant effects are reported in the manuscript, detailed genotype-specific effects are shown in the tables). Because of the large number of comparisons being performed, we used a relatively stringent type I error criteria (p < 0.0005) for evaluating the significance of any single association. (This "corrects" for performing approximately 100 independent tests, close to the number of tagSNPs genotyped for both genes). The strongest associations between individual SNPs and breast cancer risk were with SNP34 (rs9466314) in "block 2" of PRL (co-dominant effect OR, 1.48; 95% CI, 1.00–2.18; p = 0.049) and SNP49 (rs34024951) in block 3 of PRLR (co-dominant effect OR, 0.85; 95% CI, 0.73–0.99; p = 0.032) (Table 1). Of note, SNP34 in PRL was only observed among AAs, with a MAF of 6% in cases and 5% in controls in our sample. The missense Ile^100^Val SNP in PRLR was not associated with breast cancer risk (co-dominant effect OR, 1.02; 95%CI, 0.83–1.24; p = 0.883) (Additional File 1, Table S5).

**Table 1:** Nominally significant associations between prolactin (PRL) and prolactin receptor (PRLR) tagSNPs and breast cancer risk

SNP	AA		NH		JA		LA		WH		OR (95%CI)^a^
	% cases	% controls	% cases	% controls	% cases	% controls	% cases	% controls	% cases	% controls	
**PRL**											
"BLOCK 2"											
SNP 34	(rs9466314)										
AA	85.96	90.80	99.07	100.00	100.00	100.00	97.90	98.44	100.00	99.54	ref
AT	13.45	8.73	0.93	0.00	0.00	0.00	2.10	1.56	0.00	0.46	1.54 (1.02–2.34)
TT	0.58	0.47	0.00	0.00	0.00	0.00	0.00	0.00	0.00	0.00	1.29 (0.18–9.22)
**PRLR**											*P trend = 0.049*
BLOCK 3											
SNP 49	(rs34024951)										
CC	51.63	46.57	93.4	88.97	86.76	86.19	84.34	83.77	84.96	84.02	ref
CT	43.03	42.79	6.60	11.03	12.77	13.81	15.06	15.18	14.79	15.30	0.92 (0.77–1.10)
TT	5.34	10.64	0.00	0.00	0.47	0.00	0.60	1.05	0.25	0.68	0.50 (0.30–0.83)
											***P trend = 0.032***

AA, African Americans; NH, Native Hawaiians; JA, Japanese Americans; LA, Latinas, WH, Whites.

^a ^ORs adjusted for age and ethnicity.

^b ^P-value for test of trend.

We performed haplotype analyses using the most common haplotype as the reference group (Additional File 1, Tables S10 and S11); results were similar when we used all other haplotypes as the reference group (data not shown). In the analysis of the common haplotypes, haplotype 3I of PRL was nominally associated with risk (OR, 1.27; 95%CI, 1.02–1.59; p = 0.036) (Additional File 1, Table S10). This haplotype was only common in NH (14%) and JA (18%), and the effect was observed only in JA (OR, 1.39; 95%CI, 1.07–1.81; p = 0.015; p-heterogeneity = 0.193). No haplotypes in PRL or PRLR haplotypes were significantly associated with breast cancer risk using our type I error criteria (p < 0.0005) (Additional File 1, Tables S10 and S11).

### Plasma prolactin level analysis

Among the 362 postmenopausal controls in the biomarker analysis, the median plasma PRL level was 8.1 ng/mL. Prolactin levels did not vary by race/ethnicity, before or after adjusting for potential confounders: parity, age at first pregnancy, body mass index, family history of breast cancer, and menopause age and type (p-heterogeneity = 0.447) (data not shown). The strongest association between a single SNP and PRL levels was with SNP44 (rs2244502) of PRL, which showed approximately a 50% increase in levels between minor allele homozygotes versus major allele homozygotes (Additional File 1, Tables S6 and S7). We also observed nominally significant associations between prolactin levels and seven SNPs in PRL (SNP33, SNP34, SNP39, SNP44, SNP54, SNP62, SNP65) and two SNPs (SNP73, SNP148) in PRLR (Table 2). None of these associations were significant at the p < 0.0005 level.

**Table 2:** Nominally significant associations between prolactin (PRL) and prolactin receptor (PRLR) tagSNPs and plasma PRL levels

SNP	SNP Name	N (%)	Genotype	LS means^a ^(95% CI)		p^b^
**PRL**						
"BLOCK" 2						
SNP 33	rs1341238	167 (64.98)	GG	7.15	(6.35 – 8.06)	0.025
		65 (25.29)	AG	9.23	(7.68 – 11.09)	
		25 (9.73)	AA	9.02	(6.69 – 12.16)	
SNP 34	rs9466314	248 (96.50)	AA	7.67	(6.94 – 8.49)	0.039
		8 (3.11)	AT	10.48	(6.25 – 17.58)	
		1 (0.39)	TT	31.75	(7.69 – 131.02)	
SNP 39	rs3756824	246 (81.19)	CC	7.56	(6.86 – 8.34)	0.035
		56 (18.48)	CG	9.73	(8.00 – 11.82)	
		1 (0.33)	GG	6.15	(1.55 – 24.49)	
SNP 44	rs2244502	145 (47.85)	AA	6.96	(6.15 – 7.88)	0.002
		126 (41.58)	AT	8.64	(7.60 – 9.82)	
		32 (10.56)	TT	10.30	(7.91 – 13.41)	
BLOCK 3						
SNP 54	rs849886	83 (25.54)	CC	9.33	(7.83 – 11.13)	0.048
		157 (48.31)	CT	8.22	(7.31 – 9.24)	
		85 (26.15)	TT	7.27	(6.15 – 8.59)	
BLOCK 4						
SNP 62	rs849870	191 (64.09)	CC	8.72	(7.81 – 9.72)	0.013
		96 (32.21)	CT	7.28	(6.30 – 8.41)	
		11 (3.69)	TT	5.66	(3.69 – 8.70)	
SNP 65	rs849872	142 (47.65)	TT	8.96	(7.92 – 10.14)	0.018
		123 (41.28)	CT	7.47	(6.59 – 8.48)	
		33 (11.07)	CC	6.93	(5.42 – 8.85)	
**PRLR**						
BLOCK 4						
SNP 73	rs10941235	135 (49.45)	CC	8.97	(7.78 – 10.33)	0.042
		107 (39.19)	CT	7.09	(6.13 – 8.20)	
SNP 148	rs37364	136 (49.1)	TT	7.34	(6.44 – 8.35)	0.037
		106 (38.27)	GT	8.49	(7.32 – 9.86)	
		35 (12.64)	GG	9.72	(7.50 – 12.6)	

AA, African Americans; NH, Native Hawaiians; JA, Japanese Americans; LA, Latinas, WH, Whites.

^a ^Least square means of prolactin levels and 95% confidence interval; LS means adjusted for age, ethnicity, and assay batch.

^b ^P-value for test of trend.

## Discussion

We genotyped a high density of SNPs to characterize the haplotype structure of PRL and PRLR genes, using the criterion for haplotype-based studies described by Gabriel et al. [43] and the multivariate R_h_^2 ^statistic [44] to provide high predictability of the common haplotypes in PRL and PRLR. We found that in almost all ethnic groups and for both genes, the selected tagSNPs performed well in predicting the common SNPs typed in the LD characterization phase (average multi-marker R^2 ^= 0.95) and the common haplotypes defined by the tagSNPs (average minimum R_h_^2 ^= 0.87).

Assuming an average multi-marker R^2 ^= 0.90 between causal alleles and tagSNPs or haplotype predictors, we had 96% power to detect relative risks of 1.29 per haplotype or genotype copy with 10% frequency, allowing for a 5% type I error rate. However, given the large number of statistical tests for each gene, we expected several false positive associations. By a more stringent type I error criteria (p < 0.0005) the detectable relative risk, at 90% power, for a dominant allele with 10% frequency, is 1.45 per copy. By ethnic group, we had 78–82% power to detect large ORs ≥ 2.1 (except in NH, ORs ≥ 3.0) with this significance level. The purpose of this study however, was to assess shared common genetic variation across ethnic groups. For PRL levels among 362 controls, only fairly large differences in mean levels could be detected with good power. For example, after correcting for 100 comparisons (e.g. using p < 0.0005), we estimate that we had 90% power to detect an association between PRL levels and a common (10%) variant only when that variant was associated with approximately a 50% change in mean levels per genotype/haplotype copy.

A recent German study of 441 cases and 552 controls reported an increase in breast cancer risk associated with genetic variation in PRL: rs1341239 (SNP35) (OR, 1.67; 95%CI, 1.11–2.50 for homozygous individuals) and rs12210179 (OR, 2.09; 95%CI, 1.23–3.52), which we did not genotype in our sample. SNP35 has been shown to be functionally significant in relation to Systemic Lupus Erythematosus (SLE) [45,46]. Vaclavicek et al. reported that rs12210179 does not lie within any transcription binding site and is in high LD (|D'| = 0.91) with SNP35 [47]. Among Whites in the MEC, SNP35 is well predicted by tagSNP33, pairwise R^2 ^= 0.86. Using HapMap data [48], rs12210179 is common (27%) among Caucasians (vs. Yorubans 4%, Japanese 1%) and for Caucasians, is well predicted by tagSNP43 (pairwise R^2 ^= 1.00). Though we did not test these SNPs directly in our study, using these "surrogate" tagSNPs, we did not find any significant association with breast cancer risk among Whites (tagSNP33: OR 0.96; 95%CI, 0.80–1.16, p = 0.705; tagSNP43: OR 0.98; 95%CI, 0.78–1.23, p = 0.879) or overall (tagSNP33: OR 1.03; 95%CI, 0.93–1.14, p = 0.584; tagSNP43: OR 1.07; 95%CI, 0.93–1.22, p = 0.346).

Vaclavicek et al. also reported a TGTG haplotype in PRL comprised of rs1341239 (SNP35), rs12210179 (not genotyped in our sample), rs2244502 (tagSNP44), and rs1205960 (tagSNP56) associated with breast cancer risk (OR, 1.42; 95%CI, 1.07 – 1.90) [47]. This haplotype falls in "block" 2 and block 3 of our characterization of the PRL locus (Additional File 1, Table S1). Using 11 tagSNPs for "block 2" (multi-marker R^2 ^= 0.79–1.00 for Whites) and 7 tagSNPs for block 3 (multi-marker R^2 ^= 0.92–1.00 for Whites), we did not observe an association with breast cancer risk (Additional File 1, Table S10). We used "surrogate" tagSNPs 33, 43, 44, and 56 to best approximate the TGTG haplotype but did not observe an association between common surrogate haplotypes and breast cancer risk among Whites (global test p = 0.78) or overall (global test p = 0.70). Further studies are needed to directly evaluate the TGTG haplotype in relation to breast cancer risk, especially among Whites.

We found that tagSNP34 (2.1 kb upstream of SNP35 in the promoter region of PRL) had the strongest association with risk of breast cancer (p = 0.049). It is possible that this SNP may be functionally significant as both SNP34 and SNP35 lie in the distal extra-pituitary promoter region of prolactin. However, this SNP was only observed among AAs, with a minor allele frequency (MAF) of 6% in cases and 5% in controls in our sample. Further studies are needed to assess the relevance of this finding. The strongest association in PRL between a haplotype and breast cancer risk was with haplotype 3I in block 3 (p = 0.036). This haplotype was only observed in JA and NH, and the association with risk was confined to JA.

For PRLR, the only missense SNP previously described in relation to breast cancer risk is a Leu^150^Ile SNP in exon 6 which was reported in 2 of 38 cases in a Turkish study [41]. In our large sample, this SNP was monomorphic; however, it is possible that it is rare or only observed in certain populations.

Vaclavicek et al. also reported a protective TCC haplotype in PRLR (OR, 0.69; 95%CI, 0.54–0.89; p = 0.004) using just three tagSNPs. The TCC haplotype consists of rs13354826 (not genotyped in our sample, block 2), rs9292573 (SNP59, block 3), and rs37389 (SNP141, block 7). In Whites, these SNPs are well predicted: rs13354826 (tagSNPs 7 and 35, HapMap data, multi-marker R^2 ^= 1.00), SNP59 (tagSNP55, pairwise R^2 ^= 1.00), and SNP141 (tagSNP139, pairwise R^2 ^= 0.94). We used "surrogate" tagSNPs 7, 35, 55, and 139 to approximate the TCC haplotype and found that the common haplotypes comprised of these surrogate SNPs were not significantly associated with risk. Though we are unable to form a direct prediction of the TCC haplotype, we believe that our approach is comprehensive enough to have detected a true association within this region of the strength reported by Vaclavicek et al. Using 56 tagSNPs across high density coverage of 210 kb of the PRLR locus (25 kb upstream of first alternative exon E1_3 _to 10 kb downstream of exon 11), we did not find an association between SNPs or haplotypes in PRLR and breast cancer risk.

We did not generate convincing evidence of an association between PRL levels and common genetic variation in PRL and PRLR, although our study was limited by small sample size. The most significant p-value was 0.002 for SNP44 in PRL, which corresponds to a 48% increase in PRL levels between major and minor allele homozygotes. The Nurses Health Study [16] demonstrated that > 1.6-fold difference between upper and lower quartiles of PRL levels was associated with a 34% increase in breast cancer risk. We did not observe an association between breast cancer risk and SNP44 (p = 0.575). However, even if the association between SNP44 and prolactin levels were correct, and assuming a direct influence of genetically determined prolactin levels on breast cancer risk consistent with the Nurses Health Study, the 48% increase in PRL levels for minor allele homozygotes of SNP44 would still only correspond to a 10% risk increase between carriers and non-carriers of two copies. Such an increase in risk is not detectable in this study with reasonable power, which could explain the apparent lack of association between SNP44 and breast cancer risk in this study. Further studies in larger samples are needed to definitively assess the relationship between this polymorphism, plasma PRL levels and breast cancer. In addition, our results may not be generalizable to premenopausal women since we only included postmenopausal women in our analysis. Prolactin levels have been shown to decline slightly among postmenopausal women compared to premenopausal women [2]. However, the NHS study evaluated prolactin levels among premenopausal and postmenopausal women and found no difference in risk of breast cancer by menopausal status: premenopausal (RR 1.3, 95% CI 0.9–1.9) vs. postmenopausal (RR 1.3, 95% CI 1.0–1.8) women [16,29]. It is unclear whether we could draw similar conclusions from our study population.

Strengths of this study include the large case-control sample size, comprehensive assessment of LD block structure, and tagSNP selection providing excellent prediction of nearly all SNPs or common haplotypes, across five racial/ethnic populations. However, the ability to definitively evaluate ethnic-specific risks and associations with plasma PRL levels should be interpreted with caution, due to the small number of subjects in these groups. Further studies using larger samples of PRL levels are needed to assess the relationship with polymorphisms in the PRL and PRLR genes, and in particular, to validate the association observed between PRL levels and SNP44 in PRL.

## Conclusion

This the largest and most comprehensive study of common genetic variation in PRL pathway genes in relation to breast cancer risk and plasma PRL levels. In contrast to a recent study of PRL and PRLR in relation to breast cancer, we observed no strongly significant associations with breast cancer risk. We also did not find an association between common genetic variation in PRL or PRLR and circulating plasma PRL levels. Our results emphasize the importance of using high density genotyping to adequately characterize genes for use in association studies and caution against false positive results when interpreting these data. Though we did not observe an association with breast cancer risk, results from our study provide a framework for future association studies of PRL pathway genes in relation to other diseases (such as Systemic Lupus Erythematosus) and for larger studies of plasma PRL levels.

## Methods

### Subjects

The MEC consists of over 215,000 men and women in Hawaii and Los Angeles (with additional African-Americans from elsewhere in California) and has been previously described in detail [49]. The cohort is mainly comprised of five self-described racial-ethnic populations: Native Hawaiians, Japanese-Americans and Whites from Hawaii, and African-Americans, Japanese-Americans and Latinos from Los Angeles. Between 1993 and 1996, participants entered the MEC by completing a self-administered mail questionnaire that asked detailed information about dietary habits, demographic factors, personal behaviors, history of prior medical conditions, family history of common cancers, and for women, reproductive history and exogenous hormone use. The participants were between the ages 45 and 75 when they entered the cohort.

Incident cancers in the MEC are identified by record linkage to the Hawaii Tumor Registry, the Cancer Surveillance Program for Los Angeles County, and the California State Cancer Registry. These population-based tumor registries participate in the National Cancer Institute's Surveillance, Epidemiology and End Results (SEER) program of cancer registration which is known to have an excellent (98%) case ascertainment. From the registries we also obtained information about stage of disease at diagnosis. Breast cancer cases were classified as "advanced" cases when diagnosed with invasive/non-localized disease (SEER stage ≥ 2) at diagnosis.

Beginning in 1996, blood samples were collected from incident breast cancer cases. At this time, blood collection was also initiated in a random sample of MEC participants to serve as a control pool for genetic analyses. The participation rates for providing blood sample were ≥ 65% for cases and controls. Demographic characteristics related to socio-economic status and acculturation (e.g. age at cohort entry, education, place of birth, and years living in the United States) were similar among those who provided a blood sample and women in the entire cohort. Eligible breast cancer cases in this study consisted of women with incident breast cancer diagnosed after enrollment in the MEC through April 2002. Controls were women without breast cancer prior to entry into the cohort and without a cancer diagnosis up to April 2002, and were frequency matched to cases by age and ethnicity. Because < 6% of cohort members have moved outside of the Hawaii and Los Angeles between enrollment (1993–1996) and the cut-off date for diagnosis (April 2002) the likelihood of missing cases that accrued in the cohort over this period of time is low.

The study consists of 1,615 invasive breast cancer cases (345 African Americans, 425 Japanese Americans, 335 Latinas, 109 Native Hawaiians, and 401 Whites) and 1,962 controls. By racial/ethnic group, the number of cases and controls were 345/426 AA, 109/290 NH, 425/420 JA, 335/386 LA, and 401/440 WH. The study protocol was approved by the Institutional Review Boards at the University of Hawaii and at the University of Southern California.

Subjects included in the analysis of plasma PRL levels were a random sample of the controls in the case-control panel. A total of 500 postmenopausal women with previously collected biospecimens (100 in each ethnic group) were included. Women reporting hormone therapy use at blood draw were excluded (n = 128), and individuals with PRL levels that were 2.5-fold outside the normal range were excluded (n = 10).

### Gene Sequencing

We sequenced the exons and splice-site regions of PRL and PRLR in germline DNA from 95 advanced breast cancer cases (19 of each racial/ethnic group). We used DNA samples from advanced cases to increase the probability of discovering single nucleotide polymorphisms (SNPs) that are biologically relevant to breast cancer. Sequencing was performed using ABI BigDye terminator chemistry on the ABI 3730 DNA Analyzer (Applied Biosystems, Foster City, CA). The PolyPhred program was used to identify polymorphisms with manual review by at least two observers, and all putative coding variants were validated by genotyping in the same panel of advanced cases and in the multiethnic panel (discussed below).

### Characterization of Linkage Disequilibrium and Haplotype Patterns

We used a haplotype-based approach to study common variation in PRL and PRLR in the MEC, previously described elsewhere [42]. We selected single nucleotide polymorphisms (SNPs) from both the public (National Center of Biotechnology Information [50]) and private (Celera [51]) databases to construct high density SNP maps that included up to 20 kilobases (kb) upstream of the transcription initiation site and 10 kb downstream of the last exon of each gene, for a total coverage of 59 kb in PRL and 210 kb in PRLR. Block structure was assessed using SNPs with MAF ≥ 10%. Blocks were initially defined following alignment across racial/ethnic groups; borders were characterized by SNPs at the extreme ends of the block in any one ethnic group, except for African-Americans, whose block sizes, as expected, were modestly smaller than the other groups. We tested the suitability of this block definition by evaluating whether SNPs surrounding presumed block borders modified the number or identity of common haplotypes estimated within the blocks; changes in the number of haplotypes and the introduction of recombinant haplotypes would indicate whether SNPs were spanning a potentially important site of historical recombination and guided us in redefining a block boundary.

We genotyped common SNPs (MAF > 5% in at least one racial/ethnic group) at a density of 1 SNP every ~1 kb on average across the locus, all known missense SNPs in public database, and all newly identified missense SNPs in our sequencing effort. In total, 139 (PRL) and 276 (PRLR) SNPs were selected and genotyped in a multiethnic panel of 349 women in the MEC without a history of cancer (n = 69–70 per racial-ethnic group). This sample size allows > 99% power to detect common haplotypes (≥ 5% frequency) that are shared across all ethnic groups, and about 90% power to detect common ethnic-specific haplotypes. Of these SNPs, 36 (PRL) and 74 (PRLR) were identified as monomorphic and 17 (PRL) and 22 (PRLR) genotyped poorly (SNPs missing genotype data for ≥ 25% of samples or out of Hardy-Weinberg equilibrium more than one of the populations, p ≤ 0.01). This left 80 (PRL) and 173 (PRLR) SNPs with MAF = 5% in at least one racial-ethnic group to be included in the haplotype analysis.

The |D'| and r^2 ^statistics were used to assess pairwise linkage disequilibrium (LD) between the common SNPs. Within regions of strong LD [43], haplotype frequency estimates were constructed from the genotype data in the multiethnic panel (one ethnicity at a time) using the expectation-maximization (E-M) algorithm of Excoffier and Slatkin [52]. The squared correlation (R_h_^2^) between the true haplotypes (h) and their estimates were then calculated as described by Stram *et al.*[44]. "Tagging" SNPs (tagSNPs) for the case-control study were then chosen by finding the minimum set of SNPs for each ethnic group that would have R_h_^2 ^> 0.7 for all common haplotypes with an estimated frequency of ≥ 5%. TagSNP selection was performed using the tagSNPs program [53].

Values of the multi-marker and pairwise R^2 ^values between tagSNPs and unmeasured SNPs were calculated using the Tagger algorithm [40] in Haploview and the slightly more general method given in Stram 2004 [54].

### Genotyping

DNA for all subjects was extracted from white blood cell fractions using the Qiagen Blood Kit (Qiagen, Chatsworth, CA). SNP genotyping in the multiethnic panel was performed using the Sequenom (Sequenom Inc, San Diego, CA) platform. Tag SNP genotyping in the breast cancer cases and controls was performed by the 5' nuclease TaqMan allelic discrimination assay (ABI7900) and the Illumina (Illumina Inc, San Diego, CA) platforms. Replicate blinded quality control samples (5%) were included to assess reproducibility of the genotyping procedure; the concordance was ≥ 99.7% for all platforms.

### Plasma Prolactin Measurements

Prolactin was measured using a double-antibody, immunoradiometric assay from Diagnostic System Laboratories (Webster, Texas) in hormone analysis laboratories at the International Agency for Research on Cancer. The assay was performed in multiple batches with equal numbers of each population in each batch. The theoretic sensitivity (as stated by the manufacturer) is 0.1 ng/ml. Mean intra- and inter-batch coefficients of variation were 5.4% and 12.8% respectively, using 25 microliters sample volumes. Plasma PRL levels have been shown to be stable in whole blood for 24–48 hours [55]. In the MEC, time from blood collection to processing was no more than six hours.

### Statistical Analysis

Haplotype frequencies among breast cancer cases and controls were estimated using the tagSNPs selected to distinguish the common haplotypes (≥ 5% frequency) for each ethnic group in the multiethnic panel as described [56]. The E-M algorithm was used to estimate haplotype frequencies for the tagSNPs in the combined dataset (cases + controls) and individual estimates of haplotype count (expected number of copies of each haplotype carried by each individual) from the E-M were outputted to an external file and merged with case-control status. These estimates were then used as explanatory variables in logistic regression models.

As shown empirically [57], the majority of common variation is shared across racial and ethnic populations [57,58] while the biological effects on risk for the majority of common disease-associated alleles have also been shown to be consistent across populations [59]. These observations justify pooling genetic data across racial and ethnic populations if no heterogeneity is noted. To assess the consistency of genetic effects across populations, we first tested for heterogeneity across racial-ethnic groups prior to pooling genetic data. These tests were performed using a likelihood ratio test following the inclusion of an interaction term between the each haplotype (or SNP) and ethnicity in the logistic regression model. Pooled odds ratios (ORs) and 95% confidence intervals (CIs) were then estimated for each haplotype and tagSNP using unconditional logistic regression adjusted for age and ethnicity. Because of the large number of comparisons being performed we used a relatively stringent type I error criteria (p < 0.0005) for evaluating the significance of any single association. (This "corrects" for performing approximately 100 independent tests, close to the number of tagSNPs genotyped for both genes).

We used the methods described by Zaykin et al. to perform global tests of association between haplotypes and cancer risk within each LD block and to estimate haplotype-specific odds ratios [60]. ORs were estimated for each common haplotype using the most common haplotype as the reference group and for each SNP using the more common genotype as the reference group. We also performed the haplotype analyses using all other haplotypes as the reference group and performed individual SNP analyses for co-dominant effects, both of which yielded similar results (data not shown). Because further adjustment for study area (Hawaii or Los Angeles) and the established breast cancer risk factors (first-degree family history of breast cancer, body mass index, parity, age at first birth, age at menarche, type and age at menopause, use of hormone replacement therapy, and alcohol consumption) did not impact our results, we only present results from the age- and ethnicity-adjusted models.

We also calculated the effect of SNPs and estimated haplotypes on plasma PRL levels using generalized linear models adjusted for continuous (age, anthropometry) and categorical (reproductive history) variables. The hormone measurements were log-transformed to best approximate a normal distribution. These values were transformed back to normal physiologic values for presentation. Means are presented as least-squares means (LS means). For all analyses, a dominant, co-dominant, and recessive model were fitted.

The haplotype frequencies and counts were estimated using tagSNPs program [53]. All other statistical analyses were conducted using SAS version 9.1 (SAS Institute, Cary, NC).

## Abbreviations

PRL: prolactin; PRLR: prolactin receptor; MEC: Multiethnic Cohort; AA: African-Americans; NH: Native Hawaiians; JA: Japanese-Americans; LA: Latinos; WH: Whites; SNP: single nucleotide polymorphism; tagSNP: "tagging" SNP; LD: linkage disequilibrium; E-M: expectation-maximization; OR: odds ratio; CI: confidence interval.

## Competing interests

The author(s) declare that they have no competing interests.

## Authors' contributions

SAL, CAH, LNK, MCP, BEH helped conceive the study aims and design. CAH, NPB, LCP, DA, JNH, BEH coordinated the genetic studies at the Broad Institute and the University of Southern California. SAL, NPB, LCP performed genotyping. SAL, CAH, MCP, DA, JNH, BEH, DOS helped perform tagSNP selection. MCP and DOS provided expertise in genotyping and results analyses. SAL and DOS performed the statistical analyses. SAL, CAH, DA, JNH, BEH, DOS interpreted the data. SAL, CAH, JNH, DOS drafted the manuscript. IC helped create the LD plot figures using LocusView. All authors have given final approval of the version to be published.

## Pre-publication history

The pre-publication history for this paper can be accessed here:



## Supplementary Material

Additional file 1 Supplemental Tables S1-S15. Click here for file

Additional file 2 Linkage disequilibrium (LD) plot across the prolactin (PRL) locus for African-Americans. The horizontal black line depicts the 59-kilobase region of chromosome (chr) 6 analyzed in our multiethnic panel. The PRL gene is shown in grey (RefSeq gene = completed genes from the human genome assembly). Alternative exon 1a (associated with the distal extra-pituitary promoter region) lies 5.8 kb upstream of exon 1 (associated with the pituitary promoter region). The LD plot, presented at the bottom of the figures, is based on the measure of *D*'. Each diamond indicates the pairwise magnitude of LD, with dark grey indicating strong LD (*D*' > 0.8) and a logarithm of odds score of greater than 2.0. Single nucleotide polymorphisms (SNPs) < 5% for this ethnic group not shown. (Figure prepared with LocusView, Broad Institute, Cambridge, MA, unpublished software by T. Petryshen, A. Kirby, and M. Ainscow [61]). Click here for file

Additional file 3 Linkage disequilibrium (LD) plot across the prolactin (PRL) locus for Native-Hawaiians. The horizontal black line depicts the 59-kilobase region of chromosome (chr) 6 analyzed in our multiethnic panel. The PRL gene is shown in grey (RefSeq gene = completed genes from the human genome assembly). Alternative exon 1a (associated with the distal extra-pituitary promoter region) lies 5.8 kb upstream of exon 1 (associated with the pituitary promoter region). The LD plot, presented at the bottom of the figures, is based on the measure of *D*'. Each diamond indicates the pairwise magnitude of LD, with dark grey indicating strong LD (*D*' > 0.8) and a logarithm of odds score of greater than 2.0. Single nucleotide polymorphisms (SNPs) < 5% for this ethnic group not shown. (Figure prepared with LocusView, Broad Institute, Cambridge, MA, unpublished software by T. Petryshen, A. Kirby, and M. Ainscow [61]). Click here for file

Additional file 4 Linkage disequilibrium (LD) plot across the prolactin (PRL) locus for Japanese-Americans. The horizontal black line depicts the 59-kilobase region of chromosome (chr) 6 analyzed in our multiethnic panel. The PRL gene is shown in grey (RefSeq gene = completed genes from the human genome assembly). Alternative exon 1a (associated with the distal extra-pituitary promoter region) lies 5.8 kb upstream of exon 1 (associated with the pituitary promoter region). The LD plot, presented at the bottom of the figures, is based on the measure of *D*'. Each diamond indicates the pairwise magnitude of LD, with dark grey indicating strong LD (*D*' > 0.8) and a logarithm of odds score of greater than 2.0. Single nucleotide polymorphisms (SNPs) < 5% for this ethnic group not shown. (Figure prepared with LocusView, Broad Institute, Cambridge, MA, unpublished software by T. Petryshen, A. Kirby, and M. Ainscow [61]). Click here for file

Additional file 5 Linkage disequilibrium (LD) plot across the prolactin (PRL) locus for Latinas. The horizontal black line depicts the 59-kilobase region of chromosome (chr) 6 analyzed in our multiethnic panel. The PRL gene is shown in grey (RefSeq gene = completed genes from the human genome assembly). Alternative exon 1a (associated with the distal extra-pituitary promoter region) lies 5.8 kb upstream of exon 1 (associated with the pituitary promoter region). The LD plot, presented at the bottom of the figures, is based on the measure of *D*'. Each diamond indicates the pairwise magnitude of LD, with dark grey indicating strong LD (*D*' > 0.8) and a logarithm of odds score of greater than 2.0. Single nucleotide polymorphisms (SNPs) < 5% for this ethnic group not shown. (Figure prepared with LocusView, Broad Institute, Cambridge, MA, unpublished software by T. Petryshen, A. Kirby, and M. Ainscow [61]). Click here for file

Additional file 6 Linkage disequilibrium (LD) plot across the prolactin (PRL) locus for Whites. The horizontal black line depicts the 59-kilobase region of chromosome (chr) 6 analyzed in our multiethnic panel. The PRL gene is shown in grey (RefSeq gene = completed genes from the human genome assembly). Alternative exon 1a (associated with the distal extra-pituitary promoter region) lies 5.8 kb upstream of exon 1 (associated with the pituitary promoter region). The LD plot, presented at the bottom of the figures, is based on the measure of *D*'. Each diamond indicates the pairwise magnitude of LD, with dark grey indicating strong LD (*D*' > 0.8) and a logarithm of odds score of greater than 2.0. Single nucleotide polymorphisms (SNPs) < 5% for this ethnic group not shown. (Figure prepared with LocusView, Broad Institute, Cambridge, MA, unpublished software by T. Petryshen, A. Kirby, and M. Ainscow [61]). Click here for file

Additional file 7 Linkage disequilibrium (LD) plot across the prolactin receptor (PRLR) locus for African-Americans. The horizontal black line depicts the 210-kilobase region of chromosome (chr) 5 analyzed in our multiethnic panel. The PRLR gene is shown in grey (RefSeq gene = completed genes from the human genome assembly). Alternative first exons are shown in black below the gene: hE13, hE1N1, hE1N2, hE1N3, hE1N4, and hE1N5. The LD plot, presented at the bottom of the figures, is based on the measure of *D*'. Each diamond indicates the pairwise magnitude of LD, with dark grey indicating strong LD (*D*' > 0.8) and a logarithm of odds score of greater than 2.0. Single nucleotide polymorphisms (SNPs) < 5% for this ethnic group not shown. (Figure prepared with LocusView, Broad Institute, Cambridge, MA, unpublished software by T. Petryshen, A. Kirby, and M. Ainscow [61]). Click here for file

Additional file 8 Linkage disequilibrium (LD) plot across the prolactin receptor (PRLR) locus for Native-Hawaiians. The horizontal black line depicts the 210-kilobase region of chromosome (chr) 5 analyzed in our multiethnic panel. The PRLR gene is shown in grey (RefSeq gene = completed genes from the human genome assembly). Alternative first exons are shown in black below the gene: hE13, hE1N1, hE1N2, hE1N3, hE1N4, and hE1N5. The LD plot, presented at the bottom of the figures, is based on the measure of *D*'. Each diamond indicates the pairwise magnitude of LD, with dark grey indicating strong LD (*D*' > 0.8) and a logarithm of odds score of greater than 2.0. Single nucleotide polymorphisms (SNPs) < 5% for this ethnic group not shown. (Figure prepared with LocusView, Broad Institute, Cambridge, MA, unpublished software by T. Petryshen, A. Kirby, and M. Ainscow [61]). Click here for file

Additional file 9 Linkage disequilibrium (LD) plot across the prolactin receptor (PRLR) locus for Japanese-Americans. The horizontal black line depicts the 210-kilobase region of chromosome (chr) 5 analyzed in our multiethnic panel. The PRLR gene is shown in grey (RefSeq gene = completed genes from the human genome assembly). Alternative first exons are shown in black below the gene: hE13, hE1N1, hE1N2, hE1N3, hE1N4, and hE1N5. The LD plot, presented at the bottom of the figures, is based on the measure of *D*'. Each diamond indicates the pairwise magnitude of LD, with dark grey indicating strong LD (*D*' > 0.8) and a logarithm of odds score of greater than 2.0. Single nucleotide polymorphisms (SNPs) < 5% for this ethnic group not shown. (Figure prepared with LocusView, Broad Institute, Cambridge, MA, unpublished software by T. Petryshen, A. Kirby, and M. Ainscow [61]). Click here for file

Additional file 10 Linkage disequilibrium (LD) plot across the prolactin receptor (PRLR) locus for Latinas. The horizontal black line depicts the 210-kilobase region of chromosome (chr) 5 analyzed in our multiethnic panel. The PRLR gene is shown in grey (RefSeq gene = completed genes from the human genome assembly). Alternative first exons are shown in black below the gene: hE13, hE1N1, hE1N2, hE1N3, hE1N4, and hE1N5. The LD plot, presented at the bottom of the figures, is based on the measure of *D*'. Each diamond indicates the pairwise magnitude of LD, with dark grey indicating strong LD (*D*' > 0.8) and a logarithm of odds score of greater than 2.0. Single nucleotide polymorphisms (SNPs) < 5% for this ethnic group not shown. (Figure prepared with LocusView, Broad Institute, Cambridge, MA, unpublished software by T. Petryshen, A. Kirby, and M. Ainscow [61]). Click here for file

Additional file 11 Linkage disequilibrium (LD) plot across the prolactin receptor (PRLR) locus for Whites. The horizontal black line depicts the 210-kilobase region of chromosome (chr) 5 analyzed in our multiethnic panel. The PRLR gene is shown in grey (RefSeq gene = completed genes from the human genome assembly). Alternative first exons are shown in black below the gene: hE13, hE1N1, hE1N2, hE1N3, hE1N4, and hE1N5. The LD plot, presented at the bottom of the figures, is based on the measure of *D'*. Each diamond indicates the pairwise magnitude of LD, with dark grey indicating strong LD (*D*' > 0.8) and a logarithm of odds score of greater than 2.0. Single nucleotide polymorphisms (SNPs) < 5% for this ethnic group not shown. (Figure prepared with LocusView, Broad Institute, Cambridge, MA, unpublished software by T. Petryshen, A. Kirby, and M. Ainscow [61]). Click here for file

## References

[B1] Clevenger CV, Furth PA, Hankinson SE, Schuler LA (2003). The role of prolactin in mammary carcinoma. Endocr Rev.

[B2] Yen SS, Jaffe RB (1999). Reproductive endocrinology.

[B3] Muhlbock O, Boot LM (1959). Induction of mammary cancer in mice without the mammary tumor agent by isografts of hypophyses.. Cancer Res.

[B4] Boot LM, Muhlbock O, Ropcke G (1962). Prolactin and the induction of mammary tumors in mice.. General and Comparative Endocrinology.

[B5] Welsch CW, Gribler C (1973). Prophylaxis of spontaneously developing mammary carcinoma in C3H-HeJ female mice by suppression of prolactin. Cancer Res.

[B6] Welsch CW, Nagasawa H (1977). Prolactin and murine mammary tumorigenesis: a review. Cancer Res.

[B7] Liby K, Neltner B, Mohamet L, Menchen L, Ben-Jonathan N (2003). Prolactin overexpression by MDA-MB-435 human breast cancer cells accelerates tumor growth. Breast Cancer Res Treat.

[B8] Schroeder MD, Symowicz J, Schuler LA (2002). PRL modulates cell cycle regulators in mammary tumor epithelial cells. Mol Endocrinol.

[B9] Gutzman JH, Miller KK, Schuler LA (2004). Endogenous human prolactin and not exogenous human prolactin induces estrogen receptor alpha and prolactin receptor expression and increases estrogen responsiveness in breast cancer cells. J Steroid Biochem Mol Biol.

[B10] Ormandy CJ, Hall RE, Manning DL, Robertson JF, Blamey RW, Kelly PA, Nicholson RI, Sutherland RL (1997). Coexpression and cross-regulation of the prolactin receptor and sex steroid hormone receptors in breast cancer. J Clin Endocrinol Metab.

[B11] Maus MV, Reilly SC, Clevenger CV (1999). Prolactin as a chemoattractant for human breast carcinoma. Endocrinology.

[B12] Struman I, Bentzien F, Lee H, Mainfroid V, D'Angelo G, Goffin V, Weiner RI, Martial JA (1999). Opposing actions of intact and N-terminal fragments of the human prolactin/growth hormone family members on angiogenesis: an efficient mechanism for the regulation of angiogenesis. Proc Natl Acad Sci U S A.

[B13] Touraine P, Martini JF, Zafrani B, Durand JC, Labaille F, Malet C, Nicolas A, Trivin C, Postel-Vinay MC, Kuttenn F, Kelly PA (1998). Increased expression of prolactin receptor gene assessed by quantitative polymerase chain reaction in human breast tumors versus normal breast tissues. J Clin Endocrinol Metab.

[B14] Ben-Jonathan N, Liby K, McFarland M, Zinger M (2002). Prolactin as an autocrine/paracrine growth factor in human cancer. Trends Endocrinol Metab.

[B15] Clevenger CV, Chang WP, Ngo W, Pasha TL, Montone KT, Tomaszewski JE (1995). Expression of prolactin and prolactin receptor in human breast carcinoma. Evidence for an autocrine/paracrine loop. Am J Pathol.

[B16] Tworoger SS, Eliassen AH, Rosner B, Sluss P, Hankinson SE (2004). Plasma prolactin concentrations and risk of postmenopausal breast cancer.. Cancer Res.

[B17] Manjer J, Johansson R, Berglund G, Janzon L, Kaaks R, Agren A, Lenner P (2003). Postmenopausal breast cancer risk in relation to sex steroid hormones, prolactin, and SHBG (Sweden).. Cancer Causes Control.

[B18] Wang DY, De Stavola BL, Bulbrook RD, Allen DS, Kwa HG, Fentiman IS, Hayward JL, Millis RR (1992). Relationship of blood prolactin levels and the risk of subsequent breast cancer. Int J Epidemiol.

[B19] Kabuto M, Akiba S, Stevens RG, Neriishi K, Land CE (2000). A prospective study of estradiol and breast cancer in Japanese women. Cancer Epidemiol Biomarkers Prev.

[B20] Cole EN, England PC, Sellwood RA, Griffiths K (1977). Serum prolactin concentrations throughout the menstrual cycle of normal women and patients with recent breast cancer. Eur J Cancer.

[B21] Malarkey WB, Schroeder LL, Stevens VC, James AG, Lanese RR (1977). Disordered nocturnal prolactin regulation in women with breast cancer. Cancer Res.

[B22] Rose DP, Pruitt BT (1981). Plasma prolactin levels in patients with breast cancer. Cancer.

[B23] Meyer F, Brisson J, Morrison AS, Brown JB (1986). Endogenous sex hormones, prolactin, and mammographic features of breast tissue in premenopausal women. J Natl Cancer Inst.

[B24] Love RR, Rose DR, Surawicz TS, Newcomb PA (1991). Prolactin and growth hormone levels in premenopausal women with breast cancer and healthy women with a strong family history of breast cancer. Cancer.

[B25] Ingram DM, Nottage EM, Roberts AN (1990). Prolactin and breast cancer risk. Med J Aust.

[B26] Secreto G, Recchione C, Cavalleri A, Miraglia M, Dati V (1983). Circulating levels of testosterone, 17 beta-oestradiol, luteinising hormone and prolactin in postmenopausal breast cancer patients. Br J Cancer.

[B27] Bernstein L, Ross RK (1993). Endogenous hormones and breast cancer risk. Epidemiol Rev.

[B28] Helzlsouer KJ, Alberg AJ, Bush TL, Longcope C, Gordon GB, Comstock GW (1994). A prospective study of endogenous hormones and breast cancer. Cancer Detect Prev.

[B29] Tworoger SS, Eliassen AH, Sluss P, Hankinson SE (2007). A prospective study of plasma prolactin concentrations and risk of premenopausal and postmenopausal breast cancer. J Clin Oncol.

[B30] Truong AT, Duez C, Belayew A, Renard A, Pictet R, Bell GI, Martial JA (1984). Isolation and characterization of the human prolactin gene. Embo J.

[B31] Berwaer M, Martial JA, Davis JR (1994). Characterization of an up-stream promoter directing extrapituitary expression of the human prolactin gene. Mol Endocrinol.

[B32] DiMattia GE, Gellersen B, Duckworth ML, Friesen HG (1990). Human prolactin gene expression. The use of an alternative noncoding exon in decidua and the IM-9-P3 lymphoblast cell line. J Biol Chem.

[B33] Arden KC, Boutin JM, Djiane J, Kelly PA, Cavenee WK (1990). The receptors for prolactin and growth hormone are localized in the same region of human chromosome 5. Cytogenet Cell Genet.

[B34] Hu ZZ, Zhuang L, Meng J, Tsai-Morris CH, Dufau ML (2002). Complex 5' genomic structure of the human prolactin receptor: multiple alternative exons 1 and promoter utilization. Endocrinology.

[B35] Hu ZZ, Zhuang L, Meng J, Leondires M, Dufau ML (1999). The human prolactin receptor gene structure and alternative promoter utilization: the generic promoter hPIII and a novel human promoter hP(N). J Clin Endocrinol Metab.

[B36] Hu ZZ, Meng J, Dufau ML (2001). Isolation and characterization of two novel forms of the human prolactin receptor generated by alternative splicing of a newly identified exon 11. J Biol Chem.

[B37] Trott JF, Hovey RC, Koduri S, Vonderhaar BK (2003). Alternative splicing to exon 11 of human prolactin receptor gene results in multiple isoforms including a secreted prolactin-binding protein. J Mol Endocrinol.

[B38] Dunning AM, Dowsett M, Healey CS, Tee L, Luben RN, Folkerd E, Novik KL, Kelemen L, Ogata S, Pharoah PD, Easton DF, Day NE, Ponder BA (2004). Polymorphisms associated with circulating sex hormone levels in postmenopausal women. J Natl Cancer Inst.

[B39] Miller DT, Zee RY, Suk Danik J, Kozlowski P, Chasman DI, Lazarus R, Cook NR, Ridker PM, Kwiatkowski DJ (2005). Association of common CRP gene variants with CRP levels and cardiovascular events. Ann Hum Genet.

[B40] de Bakker PI, Yelensky R, Pe'er I, Gabriel SB, Daly MJ, Altshuler D (2005). Efficiency and power in genetic association studies. Nat Genet.

[B41] Canbay E, Degerli N, Gulluoglu BM, Kaya H, Sen M, Bardakci F (2004). Could prolactin receptor gene polymorphism play a role in pathogenesis of breast carcinoma?. Curr Med Res Opin.

[B42] Haiman CA, Stram DO, Pike MC, Kolonel LC, Burtt NP, Altshuler D, Hirschhorn J, Henderson BE (2003). A comprehensive haplotype analysis of CYP19 and breast cancer risk: the Multiethnic Cohort. Hum Mol Genet.

[B43] Gabriel SB, Schaffner SF, Nguyen H, Moore JM, Roy J, Blumenstiel B, Higgins J, DeFelice M, Lochner A, Faggart M, Liu-Cordero SN, Rotimi C, Adeyemo A, Cooper R, Ward R, Lander ES, Daly MJ, Altshuler D (2002). The structure of haplotype blocks in the human genome. Science.

[B44] Stram DO, Haiman CA, Hirschhorn JN, Altshuler D, Kolonel LN, Henderson BE, Pike MC (2003). Choosing haplotype-tagging SNPS based on unphased genotype data using a preliminary sample of unrelated subjects with an example from the Multiethnic Cohort Study. Hum Hered.

[B45] Stevens A, Ray D, Alansari A, Hajeer A, Thomson W, Donn R, Ollier WE, Worthington J, Davis JR (2001). Characterization of a prolactin gene polymorphism and its associations with systemic lupus erythematosus. Arthritis & Rheumatism.

[B46] Stevens A, Ray DW, Worthington J, Davis JR (2001). Polymorphisms of the human prolactin gene--implications for production of lymphocyte prolactin and systemic lupus erythematosus. Lupus.

[B47] Vaclavicek A, Hemminki K, Bartram CR, Wagner K, Wappenschmidt B, Meindl A, Schmutzler RK, Klaes R, Untch M, Burwinkel B, Forsti A (2006). Association of prolactin and its receptor gene regions with familial breast cancer. Journal of Clinical Endocrinology & Metabolism.

[B48] International HapMap Project. http://www.hapmap.org.

[B49] Kolonel LC, Henderson BE, Hankin JH, Nomura AMY, Wilkens LR, Pike MC, Stram DO, Monroe KR, Earle ME, Nagamine FS (2000). A multiethnic cohort in Hawaii and Los Angeles: Baseline Characteristics. American Journal of Epidemiology.

[B50] National Center of Biotechnology Information. http://www.ncbi.nlm.nih.gov/projects/SNP.

[B51] Celera. http://www.celera.com.

[B52] Excoffier L, Slatkin M (1995). Maximum-likelihood estimation of molecular haplotype frequencies in a diploid population. Mol Biol Evol.

[B53] TagSNPs Program. http://www-rcf.usc.edu/~stram.

[B54] Stram DO (2004). Tag SNP selection for association studies. Genet Epidemiol.

[B55] Hankinson SE, London SJ, Chute CG, Barbieri RL, Jones L, Kaplan LA, Sacks FM, Stampfer MJ (1989). Effect of transport conditions on the stability of biochemical markers in blood. Clin Chem.

[B56] Freedman ML, Penney KL, Stram DO, Le Marchand L, Hirschhorn JN, Kolonel LN, Altshuler D, Henderson BE, Haiman CA (2004). Common variation in BRCA2 and breast cancer risk: a haplotype-based analysis in the Multiethnic Cohort. Human Molecular Genetics.

[B57] International HapMap C (2005). A haplotype map of the human genome.[see comment]. Nature.

[B58] Rosenberg NA, Pritchard JK, Weber JL, Cann HM, Kidd KK, Zhivotovsky LA, Feldman MW (2002). Genetic structure of human populations.[see comment]. Science.

[B59] Ioannidis JP, Ntzani EE, Trikalinos TA (2004). 'Racial' differences in genetic effects for complex diseases.[see comment]. Nature Genetics.

[B60] Zaykin DV, Westfall PH, Young SS, Karnoub MA, Wagner MJ, Ehm MG (2002). Testing association of statistically inferred haplotypes with discrete and continuous traits in samples of unrelated individuals. Hum Hered.

[B61] LocusView. http://www.broad.mit.edu/mpg/locusview/.

