# Supplementary material for: A comprehensive analysis of common genetic variation in prolactin (PRL) and PRL receptor (PRLR) genes in relation to plasma prolactin levels and breast cancer risk: the Multiethnic Cohort
Source: BMC Med Genet. 2007 Dec 1;8:72. doi: 10.1186/1471-2350-8-72 (PMC2219987; doi:10.1186/1471-2350-8-72)
Supplement: Additional file 1 — Supplemental Tables S1-S15. [file 1471-2350-8-72-S1.doc]

**Table S1. Eighty SNPs used in the analysis of prolactin (PRL)**

|  |  |  |  |  |  | Minor allele frequency (%)  in the multiethnic panel^c^ | | | | |
| --- | --- | --- | --- | --- | --- | --- | --- | --- | --- | --- |
| SNP# | SNP | Location | Position^a^ | Nucleotide Change | Minor Allele^b^ | AA | NH | JA | LA | WH |
|  |  |  |  |  |  |  |  |  |  |  |
| 1 | rs1205945^d^ | 5' | 22434674 | C/T | T | 12.14 | 3.62 | 4.35 | 2.14 | 6.43 |
| 2 | rs2744098^d^ | 5' | 22433728 | G/T | T | 18.57 | 14.71 | 13.77 | 10.29 | 7.86 |
| 3 | rs2655420 | 5' | 22431291 | C/T | C | 25.00 | 23.53 | 28.99 | 22.06 | 11.76 |
| 4 | rs2655419^d^ | 5' | 22430714 | A/G | A | 19.85 | 23.88 | 28.36 | 20.77 | 11.43 |
| 5 | rs2655418 | 5' | 22430235 | G/T | T | 15.91 | 12.50 | 5.15 | 7.46 | 7.14 |
| 6 | rs9358533^d^ | 5' | 22429983 | C/T | T | 27.94 | 22.22 | 28.03 | 13.57 | 9.7 |
| 7 | rs2655417 | 5' | 22429107 | C/T | T | 20.29 | 18.38 | 7.25 | 8.09 | 9.29 |
| 8 | rs2655416 | 5' | 22429033 | A/G | A | 25.83 | 22.03 | 25.44 | 17.24 | 8.62 |
| 9 | rs10946546^d^ | 5' | 22428479 | C/T | T | 17.14 | 39.86 | 32.61 | 29.29 | 37.86 |
| 10 | rs2655415 | 5' | 22428301 | A/G | A | 16.67 | 13.24 | 5.07 | 8.09 | 7.86 |
| 11 | rs2744099 | 5' | 22428144 | C/T | T | 16.67 | 13.04 | 5.22 | 7.97 | 7.25 |
| 12 | rs2655414 | 5' | 22427227 | A/G | G | 16.43 | 12.50 | 5.00 | 7.86 | 7.14 |
| 13 | rs717156 | 5' | 22425601 | A/C | C | 9.65 | 6.78 | 1.54 | 3.23 | 4.55 |
| 14 | rs715226 | 5' | 22425542 | C/T | C | 19.30 | 19.49 | 27.69 | 17.21 | 8.21 |
| 15 | rs717157 | 5' | 22425494 | C/T | T | 16.67 | 12.88 | 5.07 | 5.47 | 7.25 |
| 16 | rs2744104 | 5' | 22425388 | C/G | G | 1.47 | 0.82 | 1.54 | 2.31 | 3.73 |
| 17 | rs2744105 | 5' | 22425298 | C/G | C | 22.95 | 18.42 | 25.42 | 13.89 | 7.94 |
| 18 | rs1569544 | 5' | 22424921 | A/C | A | 16.67 | 13.04 | 5.00 | 7.86 | 7.25 |
| 19 | rs1883372 | 5' | 22424437 | A/T | A | 16.67 | 13.71 | 3.45 | 9.84 | 7.03 |
| 20 | rs2655448 | 5' | 22423583 | C/T | T | 15.44 | 10.16 | 4.35 | 7.25 | 5.30 |
| 21 | rs2744106 | 5' | 22423005 | C/T | T | 16.67 | 12.50 | 4.41 | 8.09 | 5.22 |
| 22 | rs1807914 | 5' | 22421224 | A/C | C | 16.91 | 13.04 | 1.43 | 5.88 | 7.14 |
| 23 | rs2473122^d^ | 5' | 22420266 | A/G | A | 22.86 | 18.84 | 23.57 | 20.29 | 9.29 |
| 24 | rs2655433 | 5' | 22420241 | C/T | T | 22.86 | 19.57 | 23.57 | 20.71 | 9.56 |
| 25 | rs767938^d^ | 5' | 22419843 | C/T | T | 51.47 | 32.35 | 32.14 | 54.35 | 48.55 |
| 26 | rs9393273 | 5' | 22419171 | C/T | C | 16.91 | 40.58 | 35.51 | 30.00 | 39.13 |
| 27 | rs2655431 | 5' | 22418624 | C/T | T | 30.00 | 20.15 | 24.26 | 35.29 | 34.85 |
| 28 | rs9358531^d^ | 5' | 22417609 | G/T | G | 23.53 | 41.30 | 32.86 | 30.00 | 41.91 |
| 29 | rs1772116 | 5' | 22416724 | A/G | G | 6.45 | 1.79 | 0.74 | 0.78 | 2.31 |
| 30 | rs1156546^d^ | 5' | 22416671 | A/C | C | 44.12 | 34.78 | 43.57 | 35.71 | 25.00 |
| 31 | rs1209598 | 5' | 22416122 | A/T | T | 2.86 | 3.68 | 0.72 | 1.47 | 5.71 |
| 32 | rs2655426^d^ | 5' | 22415704 | G/T | T | 37.68 | 25.36 | 26.43 | 36.43 | 38.24 |
| 33 | rs1341238^d^ | 5' | 22414679 | A/G | A | 34.29 | 21.01 | 23.57 | 35.71 | 38.57 |
| 34 | rs9466314^d^ | 5' | 22414284 | A/T | T | 4.84 | 0.00 | 0.00 | 0.82 | 0.00 |
| 35 | rs1341239 | 5' | 22412183 | G/T | T | 33.33 | 14.49 | 1.43 | 22.14 | 34.78 |
| 36 | rs2744117^d^ | promoter | 22410818 | G/T | T | 3.62 | 17.42 | 28.57 | 12.5 | 9.29 |
| 37 | rs849875 | promoter | 22407979 | A/G | A | 33.06 | 14.49 | 3.85 | 20.00 | 30.47 |
| 38 | rs849876^d^ | promoter | 22407617 | A/G | G | 17.65 | 6.15 | 3.57 | 3.85 | 10.16 |
| 39 | rs3756824^d^ | promoter | 22406716 | C/G | C | 1.43 | 12.32 | 30.00 | 11.43 | 2.9 |
| 40 | rs849877 | promoter | 22406678 | C/T | C | 44.78 | 27.54 | 30.71 | 33.57 | 34.56 |
| 41 | rs11965113 | promoter | 22405857 | A/G | A | 3.68 | 0.72 | 0.00 | 0.72 | 0.00 |
| 42 | rs11968713 | intron 1 | 22404295 | A/G | G | 0.00 | 0.00 | 0.00 | 0.00 | 0.00 |
| 43 | rs12202764^d^ | intron 1 | 22403312 | A/T | A | 21.77 | 6.72 | 0.00 | 18.03 | 21.09 |
| 44 | rs2244502^d^ | intron 1 | 22402966 | A/T | T | 47.86 | 27.61 | 32.14 | 30.71 | 28.57 |
| 45 | G+4170T | intron 2 | 22401058 | G/T | G | 4.29 | 0.00 | 0.00 | 1.45 | 0.00 |
| 46 | rs1205955 | intron 2 | 22400955 | A/T | T | 36.43 | 24.64 | 10 | 18.84 | 27.94 |
| 47 | A+4346G^d^ | intron 2 | 22400881 | A/G | A | 5.80 | 0.00 | 0.00 | 0.00 | 0.00 |
| 48 | A+444152G | exon 3 | 22400776 | A/G | A | 3.62 | 0.75 | 0.00 | 0.00 | 0.00 |
| 49 | rs16885980 | intron 3 | 22400569 | A/G | G | 0.00 | 11.59 | 22.14 | 16.43 | 0.74 |
| 50 | rs7739889 | intron 3 | 22400532 | A/G | A | 15.71 | 6.52 | 0.00 | 15.00 | 20.71 |
| 51 | rs849884^d^ | intron 3 | 22400433 | C/T | T | 2.14 | 3.62 | 0.71 | 1.45 | 5.07 |
| 52 | rs7759000^d^ | intron 3 | 22399938 | C/T | C | 17.86 | 7.97 | 0.00 | 16.67 | 21.74 |
| 53 | rs849885 | intron 3 | 22399517 | A/G | G | 66.67 | 36.23 | 12.86 | 35.51 | 47.79 |
| 54 | rs849886^d^ | intron 3 | 22399346 | C/T | T | 27.94 | 52.9 | 65.00 | 47.01 | 50.00 |
| 55 | rs9379382 | intron 4 | 22397440 | C/T | C | 26.09 | 52.17 | 65.71 | 42.03 | 44.85 |
| 56 | rs1205960^d^ | intron 4 | 22396139 | C/T | T | 33.82 | 24.64 | 10.45 | 16.92 | 26.52 |
| 57 | rs6239^d^ | exon 5 | 22395724 | C/T | T | 1.47 | 11.54 | 21.01 | 16.67 | 0.78 |
| 58 | rs10946545^d^ | 3' | 22394053 | A/G | A | 2.90 | 19.57 | 22.86 | 7.97 | 7.14 |
| 59 | rs1205961^d^ | 3' | 22393991 | A/G | A | 62.86 | 29.71 | 2.14 | 33.33 | 38.97 |
| 60 | rs1205962 | 3' | 22393116 | A/T | T | 15.00 | 18.38 | 0.00 | 1.45 | 0.71 |
| 61 | rs2744118 | 3' | 22392518 | A/G | A | 39.55 | 30.00 | 37.5 | 33.87 | 35.07 |
| 62 | rs849870^d^ | 3' | 22391864 | C/T | T | 26.09 | 27.54 | 7.14 | 12.5 | 13.57 |
| 63 | rs849871 | 3' | 22391794 | A/T | T | 26.43 | 27.94 | 7.25 | 13.24 | 12.86 |
| 64 | rs6920781 | 3' | 22391251 | C/G | G | 5.71 | 7.97 | 4.29 | 10.87 | 10.87 |
| 65 | rs849872^d^ | 3' | 22391011 | C/T | C | 26.43 | 39.13 | 38.41 | 28.26 | 13.57 |
| 66 | rs849873 | 3' | 22390910 | C/T | C | 7.86 | 5.80 | 0.00 | 8.70 | 25.0 |
| 67 | rs849874 | 3' | 22390884 | C/T | C | 25.71 | 39.13 | 38.57 | 28.57 | 13.97 |
| 68 | rs2744119^d^ | 3' | 22390710 | A/G | G | 3.62 | 16.13 | 17.42 | 16.92 | 13.49 |
| 69 | rs2744120^d^ | 3' | 22389237 | A/G | G | 34.78 | 43.28 | 53.62 | 51.52 | 52.24 |
| 70 | rs12524841^d^ | 3' | 22388193 | G/T | G | 30.6 | 13.77 | 32.61 | 23.53 | 7.14 |
| 71 | rs2000132 | 3' | 22386375 | A/C | C | 12.5 | 19.67 | 0.00 | 1.54 | 1.49 |
| 72 | rs2185589 | 3' | 22385702 | C/T | T | 5.00 | 0.00 | 0.00 | 0.00 | 0.00 |
| 73 | rs2153646^d^ | 3' | 22385128 | A/T | A | 43.38 | 28.36 | 46.38 | 34.56 | 17.86 |
| 74 | rs2655447 | 3' | 22384432 | C/G | C | 11.59 | 17.65 | 0.00 | 1.43 | 1.43 |
| 75 | rs6940783^d^ | 3' | 22381923 | C/G | G | 7.86 | 8.09 | 0.00 | 12.86 | 30.00 |
| 76 | rs1832765 | 3' | 22380277 | C/T | T | 12.14 | 18.12 | 0.00 | 1.43 | 1.47 |
| 77 | rs2066266^d^ | 3' | 22378099 | A/G | G | 51.47 | 41.18 | 6.43 | 31.62 | 46.38 |
| 78 | rs2066265 | 3' | 22377996 | A/G | G | 40.71 | 15.94 | 37.14 | 21.74 | 6.43 |
| 79 | rs1417721 | 3' | 22376808 | A/C | A | 13.97 | 7.25 | 5.71 | 1.45 | 1.43 |
| 80 | rs1123886 | 3' | 22375954 | C/T | C | 19.57 | 18.12 | 5.00 | 25.74 | 40.44 |

^a^ SNP position based on March 2006 University of California, Santa Cruz, version human genome 18, chromosome 6 (http://genome.ucsc.edu).

^b^ Based on the minor allele frequency among all groups combined.

^c^ AA, African Americans; NH, Native Hawaiians; JA, Japanese Americans; LA, Latinas; WH, Whites.

^d^ TagSNP.

Boxes indicate block 1 (SNPs 1-24), “block” 2 (SNPs 25-45), block 3 (46-59), block 4 (61-77).

**Table S2. One hundred seventy-three SNPs used in the analysis of prolactin receptor (PRLR)**

|  |  |  |  |  |  | Minor allele frequency (%)  in the multiethnic panel^c^ | | | | |
| --- | --- | --- | --- | --- | --- | --- | --- | --- | --- | --- |
| SNP# | SNP | Location | Position^a^ | Nucleotide Change | Minor Allele^b^ | AA | NH | JA | LA | WH |
|  |  |  |  |  |  |  |  |  |  |  |
| 1 | rs7720958 | 5' | 35285355 | A/G | G | 42.03 | 37.68 | 20.71 | 40.58 | 71.32 |
| 2 | rs7720773 | 5' | 35285179 | A/G | G | 42.03 | 37.68 | 20.71 | 40.71 | 71.32 |
| 3 | rs10036221 | 5' | 35284766 | C/T | C | 51.49 | 37.5 | 20.29 | 38.46 | 70.71 |
| 4 | rs9986202 | 5' | 35282670 | C/G | G | 28.57 | 51.45 | 55.07 | 25.00 | 14.71 |
| 5 | rs10060999 | 5' | 35281898 | G/T | T | 3.57 | 0.00 | 0.00 | 0.00 | 0.00 |
| 6 | rs9986182^d,e^ | 5' | 35281437 | A/G | A | 16.42 | 17.42 | 5.07 | 23.81 | 36.36 |
| 7 | rs11741701^d^ | 5' | 35281220 | A/G | A | 22.46 | 21.64 | 25.36 | 18.46 | 33.82 |
| 8 | rs6897600 | 5' | 35280609 | A/C | C | 24.29 | 21.01 | 25.36 | 22.14 | 36.03 |
| 9 | rs7726240^d^ | 5' | 35277020 | C/T | C | 23.19 | 7.97 | 8.57 | 5.00 | 7.14 |
| 10 | rs7720677 | 5' | 35275958 | C/T | T | 30.30 | 49.26 | 60.00 | 42.86 | 15.44 |
| 11 | rs7734446^d^ | 5' | 35275489 | A/G | G | 5.00 | 42.75 | 46.43 | 22.14 | 5.15 |
| 12 | rs9292582^d,e^ | 5' | 35274907 | C/T | T | 14.49 | 19.40 | 5.71 | 20.77 | 35.48 |
| 13 | rs4703514 | 5' | 35274785 | A/C | C | 22.86 | 22.46 | 13.57 | 38.57 | 44.12 |
| 14 | rs9292581 | 5' | 35274536 | A/C | A | 22.46 | 22.46 | 13.57 | 38.57 | 44.12 |
| 15 | rs9292580 | 5' | 35274406 | A/G | G | 17.86 | 21.74 | 5.71 | 29.71 | 41.18 |
| 16 | rs7728298^d^ | 5' | 35274266 | A/G | G | 60.87 | 29.71 | 22.14 | 43.57 | 51.47 |
| 17 | rs10472952 | 5' | 35274122 | C/T | C | 22.46 | 22.46 | 13.57 | 38.41 | 43.28 |
| 18 | rs7727503 | 5' | 35273804 | A/G | G | 22.46 | 22.46 | 13.57 | 38.41 | 44.03 |
| 19 | rs7727306 | 5' | 35273617 | C/T | T | 17.86 | 21.32 | 5.80 | 30.15 | 41.79 |
| 20 | rs7705676 | 5' | 35273493 | C/T | C | 22.86 | 22.46 | 13.57 | 39.29 | 44.03 |
| 21 | rs7717468 | 5' | 35272006 | G/T | T | 3.57 | 0.00 | 0.00 | 0.00 | 0.00 |
| 22 | rs7712482 | 5' | 35271156 | A/G | A | 25.00 | 7.25 | 8.57 | 4.29 | 7.35 |
| 23 | rs4703398^d^ | 5' | 35270636 | A/G | A | 5.15 | 0.74 | 6.62 | 5.56 | 2.86 |
| 24 | rs6451192^d,e^ | 5' | 35270086 | A/G | G | 29.29 | 7.25 | 7.86 | 4.29 | 7.35 |
| 25 | rs10059874 | 5' | 35269543 | A/G | G | 34.78 | 6.62 | 7.86 | 4.29 | 7.35 |
| 26 | rs6870964 | 5' | 35268859 | A/T | T | 14.49 | 48.55 | 52.86 | 34.29 | 12.5 |
| 27 | rs3822482 | 5' | 35267984 | A/G | G | 26.81 | 7.25 | 7.86 | 4.29 | 7.35 |
| 28 | rs10080135 | 5' | 35267744 | C/T | T | 14.29 | 48.55 | 52.86 | 26.43 | 12.50 |
| 29 | rs7701473^d,e^ | 5' | 35267230 | G/T | G | 0.76 | 4.35 | 8.09 | 0.71 | 4.41 |
| 30 | hCV11281097^d^ | 5' | 35267223 | G/T | T | 61.76 | 56.62 | 69.29 | 37.68 | 22.86 |
| 31 | rs10068521^d^ | exon 1_3_ | 35266137 | C/G | G | 3.57 | 0.72 | 7.86 | 8.57 | 2.94 |
| 32 | rs7733450^d^ | intron 1_3_ | 35265909 | A/T | T | 10.29 | 7.97 | 9.29 | 2.14 | 8.09 |
| 33 | rs9292578 | intron 1_3_ | 35265832 | A/C | A | 3.57 | 0.72 | 7.86 | 8.57 | 2.94 |
| 34 | rs6867456 | intron 1_N1_ | 35264834 | C/G | G | 28.57 | 50.72 | 51.43 | 26.43 | 20.00 |
| 35 | rs6863018^d^ | intron 1_N1_ | 35264510 | A/G | A | 43.57 | 39.86 | 31.43 | 62.14 | 70.00 |
| 36 | rs6878684 | intron 1_N1_ | 35259599 | C/T | C | 25.71 | 50.72 | 55.71 | 27.21 | 20.00 |
| 37 | rs1604427 | intron 1_N1_ | 35259367 | C/G | C | 42.86 | 39.55 | 31.34 | 61.94 | 70.90 |
| 38 | rs931740^d^ | intron 1_N1_ | 35257797 | C/T | T | 24.64 | 51.45 | 56.43 | 26.43 | 20.00 |
| 39 | rs7731153^d^ | intron 1_N1_ | 35256622 | A/G | G | 5.00 | 42.03 | 54.29 | 12.86 | 5.00 |
| 40 | rs1587608 | intron 1_N1_ | 35254746 | A/T | T | 22.66 | 47.01 | 59.70 | 27.21 | 24.64 |
| 41 | rs6451190^d^ | intron 1_N1_ | 35253722 | C/G | G | 3.57 | 42.75 | 62.14 | 21.43 | 8.70 |
| 42 | rs1604422 | intron 1_N1_ | 35253264 | C/T | T | 3.57 | 41.3 | 60.71 | 18.57 | 7.25 |
| 43 | rs1604421 | intron 1_N1_ | 35253133 | A/G | A | 3.62 | 41.18 | 60.71 | 18.12 | 8.70 |
| 44 | rs9292574 | intron 1_N1_ | 35249253 | C/T | C | 50.72 | 39.13 | 30.00 | 55.8 | 66.91 |
| 45 | rs924599 | intron 1_N1_ | 35248441 | C/T | T | 3.62 | 41.79 | 61.76 | 18.84 | 7.14 |
| 46 | rs6866465 | intron 1_N1_ | 35247531 | C/T | C | 3.68 | 41.3 | 60.87 | 17.65 | 8.70 |
| 47 | rs1609500 | intron 1_N1_ | 35246997 | A/C | A | 50.72 | 39.13 | 30.00 | 55.00 | 66.18 |
| 48 | rs4703511 | intron 1_N1_ | 35246386 | A/G | A | 14.49 | 6.52 | 3.62 | 10.71 | 3.57 |
| 49 | rs34024951^d^ | intron 1_N1_ | 35246079 | C/T | T | 31.88 | 6.52 | 4.29 | 10.14 | 8.70 |
| 50 | rs4703396 | intron 1_N1_ | 35245411 | C/T | C | 0.71 | 6.06 | 4.41 | 10.45 | 3.73 |
| 51 | rs4703510^d^ | intron 1_N1_ | 35245262 | A/T | A | 34.33 | 47.79 | 66.18 | 27.61 | 17.14 |
| 52 | rs4235652^d^ | intron 1_N1_ | 35245096 | C/T | C | 37.68 | 52.94 | 67.65 | 28.68 | 20.29 |
| 53 | rs2047740^d^ | intron 1_N1_ | 35239149 | A/G | A | 44.29 | 39.13 | 30.00 | 57.14 | 65.71 |
| 54 | rs2047738 | intron 1_N1_ | 35238848 | C/T | C | 54.29 | 54.35 | 65.71 | 33.57 | 30.71 |
| 55 | rs875701^d^ | intron 1_N1_ | 35236413 | A/G | G | 47.76 | 39.55 | 30.15 | 56.72 | 64.39 |
| 56 | rs875700 | intron 1_N1_ | 35236177 | C/T | T | 3.62 | 41.30 | 60.71 | 19.29 | 9.42 |
| 57 | rs4703509^d^ | intron 1_N1_ | 35235951 | C/T | C | 48.57 | 19.40 | 8.70 | 28.68 | 26.47 |
| 58 | rs873456 | intron 1_N1_ | 35235414 | A/C | C | 36.07 | 38.24 | 21.64 | 48.25 | 57.41 |
| 59 | rs9292573 | intron 1_N1_ | 35235190 | A/G | A | 41.18 | 38.41 | 24.29 | 55.71 | 66.18 |
| 60 | rs1587607^d^ | intron 1_N1_ | 35234219 | A/G | G | 39.39 | 13.04 | 11.03 | 13.24 | 22.79 |
| 61 | rs7703435 | intron 1_N1_ | 35234081 | C/G | C | 3.57 | 40.58 | 60.71 | 19.57 | 8.09 |
| 62 | rs1587606 | intron 1_N1_ | 35232187 | A/G | A | 17.69 | 41.91 | 61.76 | 20.90 | 8.82 |
| 63 | rs7705216^d^ | intron 1_N3_ | 35229426 | C/G | G | 5.71 | 13.77 | 0.00 | 5.71 | 13.57 |
| 64 | rs6891939 | intron 1_N3_ | 35226742 | A/G | A | 2.90 | 40.58 | 61.43 | 20 | 8.82 |
| 65 | rs6887898 | intron 1_N3_ | 35226505 | A/G | G | 2.90 | 40.58 | 61.59 | 20 | 10.00 |
| 66 | rs7720754 | intron 1_N3_ | 35224972 | A/G | A | 3.57 | 40.58 | 61.59 | 20.29 | 10.00 |
| 67 | rs4703508 | intron 1_N3_ | 35224559 | C/T | C | 7.35 | 7.25 | 2.90 | 11.76 | 6.43 |
| 68 | rs12517356 | intron 1_N3_ | 35223746 | C/T | C | 0.00 | 0.00 | 0.00 | 6.52 | 0.00 |
| 69 | rs1610218 | intron 1_N3_ | 35222451 | C/T | T | 15.94 | 2.17 | 0.00 | 1.43 | 3.62 |
| 70 | rs10075993 | intron 1_N3_ | 35222130 | A/T | A | 3.57 | 40.58 | 63.77 | 16.43 | 5.22 |
| 71 | rs4425481 | intron 1_N3_ | 35221654 | A/C | A | 1.47 | 7.25 | 0.74 | 13.24 | 7.14 |
| 72 | rs13436213 | intron 1_N3_ | 35221583 | C/T | T | 16.43 | 14.18 | 20.71 | 28.03 | 34.29 |
| 73 | rs10941235^d^ | intron 1_N3_ | 35221337 | C/T | T | 40.58 | 35.51 | 12.86 | 30.00 | 30.88 |
| 74 | rs7713837 | intron 1_N3_ | 35219733 | C/T | C | 44.2 | 40.44 | 12.86 | 30.43 | 31.16 |
| 75 | rs7700286^d^ | intron 1_N3_ | 35219509 | C/T | C | 9.70 | 12.32 | 12.32 | 10.00 | 7.97 |
| 76 | hCV11281183 | intron 1_N3_ | 35218778 | C/T | C | 27.86 | 21.01 | 12.86 | 23.19 | 16.67 |
| 77 | rs10067710^d^ | intron 1_N3_ | 35218163 | A/T | T | 10.71 | 13.77 | 0.71 | 27.14 | 31.62 |
| 78 | rs34212252^d^ | intron 1_N3_ | 35216846 | C/G | C | 23.85 | 14.93 | 11.94 | 11.36 | 9.85 |
| 79 | rs6895193^d^ | intron 1_N3_ | 35215350 | A/G | A | 39.29 | 42.75 | 75.00 | 41.43 | 34.29 |
| 80 | rs1039429 | intron 1_N3_ | 35211735 | C/T | C | 23.48 | 14.49 | 11.76 | 10.87 | 10.00 |
| 81 | rs6451186 | intron 1_N3_ | 35211730 | C/T | T | 41.3 | 39.86 | 12.86 | 28.57 | 31.62 |
| 82 | rs4703506 | intron 1_N3_ | 35207964 | C/T | C | 42.14 | 39.86 | 12.32 | 29.29 | 31.43 |
| 83 | rs4703505 | intron 1_N3_ | 35207778 | A/G | A | 41.30 | 39.86 | 12.86 | 28.57 | 31.62 |
| 84 | rs12518881^d^ | intron 1_N3_ | 35204918 | A/G | A | 13.08 | 7.14 | 0.00 | 3.33 | 12.30 |
| 85 | rs7729932 | intron 1_N3_ | 35204127 | A/G | A | 37.14 | 39.23 | 12.32 | 28.26 | 31.62 |
| 86 | rs1039427^d^ | intron 1_N3_ | 35201692 | C/T | T | 35.38 | 38.97 | 12.32 | 26.87 | 31.34 |
| 87 | rs1039428 | intron 1_N3_ | 35201354 | G/T | T | 41.3 | 39.13 | 12.50 | 28.57 | 32.14 |
| 88 | rs1039426 | intron 1_N3_ | 35199578 | A/G | A | 36.76 | 39.13 | 12.86 | 28.26 | 31.62 |
| 89 | rs4703503 | intron 1_N3_ | 35198519 | C/T | T | 15.44 | 39.86 | 80.88 | 38.24 | 24.29 |
| 90 | rs6451185 | intron 1_N3_ | 35197908 | C/T | C | 36.23 | 16.67 | 22.86 | 16.43 | 5.15 |
| 91 | rs6451183 | intron 1_N3_ | 35197677 | A/T | A | 24.64 | 8.09 | 16.43 | 3.85 | 0.76 |
| 92 | rs6880595 | intron 1_N3_ | 35197089 | A/G | G | 21.43 | 22.46 | 0.72 | 12.14 | 24.64 |
| 93 | rs6451182 | intron 1_N3_ | 35196845 | A/T | A | 46.43 | 19.85 | 17.86 | 14.29 | 7.35 |
| 94 | rs6879521 | intron 1_N3_ | 35193247 | C/T | C | 5.00 | 0.00 | 0.00 | 0.00 | 0.00 |
| 95 | rs6897987 | intron 1_N3_ | 35192993 | A/G | G | 35.00 | 9.42 | 4.29 | 5.07 | 1.45 |
| 96 | rs7736203 | intron 1_N3_ | 35192587 | C/G | C | 2.86 | 0.00 | 0.00 | 0.71 | 0.00 |
| 97 | rs7732593 | intron 1_N3_ | 35192466 | G/T | T | 35.00 | 10.14 | 5.00 | 5.71 | 1.47 |
| 98 | rs7732013^d^ | intron 1_N3_ | 35192289 | C/T | T | 36.43 | 10.14 | 5.00 | 7.14 | 4.48 |
| 99 | rs2047741^d^ | intron 1_N3_ | 35188812 | C/G | G | 47.62 | 20.29 | 23.53 | 9.09 | 6.52 |
| 100 | rs954286 | intron 1_N4_ | 35187998 | C/T | C | 51.47 | 26.52 | 22.73 | 8.59 | 8.09 |
| 101 | rs10078077 | intron 1_N4_ | 35185662 | C/T | C | 50.82 | 26.19 | 23.44 | 8.46 | 7.46 |
| 102 | rs1587605^d^ | intron 1_N4_ | 35182622 | C/T | C | 16.67 | 9.56 | 18.94 | 5.07 | 6.43 |
| 103 | rs10059483 | intron 1_N4_ | 35181019 | C/T | C | 35.29 | 11.76 | 18.12 | 5.22 | 6.43 |
| 104 | rs2914111 | intron 1_N4_ | 35175682 | C/T | T | 50.00 | 27.94 | 23.19 | 8.09 | 7.86 |
| 105 | rs2018170 | intron 1_N4_ | 35172923 | G/T | G | 50.00 | 27.54 | 22.46 | 7.97 | 7.86 |
| 106 | rs3797212^d^ | exon 1_N5_ | 35171233 | C/T | T | 13.57 | 15.94 | 5.00 | 3.57 | 1.43 |
| 107 | rs2962092 | intron 1_N5_ | 35170188 | C/T | C | 54.29 | 27.54 | 22.86 | 9.29 | 7.86 |
| 108 | rs2962089 | intron 1_N5_ | 35168107 | C/T | C | 54.35 | 27.54 | 22.86 | 9.29 | 8.82 |
| 109 | rs2914107 | intron 1_N5_ | 35167641 | A/G | G | 38.41 | 11.59 | 17.86 | 5.71 | 6.62 |
| 110 | rs2914108 | intron 1_N5_ | 35167562 | C/T | C | 42.86 | 11.59 | 17.86 | 5.80 | 6.62 |
| 111 | rs2962086^d^ | intron 1_N5_ | 35166505 | A/G | G | 39.71 | 11.59 | 17.65 | 5.71 | 5.88 |
| 112 | rs930068^d^ | intron 1_N5_ | 35163135 | G/T | G | 8.82 | 0.74 | 0.00 | 2.24 | 5.00 |
| 113 | rs35245967 | intron 1_N5_ | 35162128 | A/G | A | 15.22 | 10.14 | 17.65 | 3.57 | 1.45 |
| 114 | rs35322400^d^ | intron 1_N5_ | 35158580 | C/T | C | 28.99 | 10.87 | 23.19 | 7.25 | 6.52 |
| 115 | rs34376897 | intron 1_N5_ | 35157467 | C/G | G | 52.24 | 27.27 | 27.27 | 8.46 | 10.29 |
| 116 | rs4703501 | intron 1_N5_ | 35155337 | A/G | A | 27.86 | 10.29 | 22.46 | 7.46 | 7.35 |
| 117 | rs1966571^d^ | intron 2 | 35153510 | C/T | C | 52.17 | 27.54 | 28.26 | 10.14 | 10.00 |
| 118 | rs33997221 | intron 2 | 35152214 | A/G | A | 52.94 | 27.94 | 27.27 | 8.59 | 10.87 |
| 119 | rs7734558^d^ | intron 2 | 35150368 | A/G | A | 20.00 | 22.22 | 1.49 | 30.00 | 55.15 |
| 120 | rs4703500^d^ | intron 2 | 35146484 | A/G | G | 24.62 | 10.00 | 20.63 | 3.79 | 1.52 |
| 121 | rs4703499 | intron 2 | 35140124 | C/T | T | 15.22 | 8.46 | 22.46 | 4.41 | 2.21 |
| 122 | rs4703390 | intron 2 | 35139654 | A/G | A | 14.71 | 10.14 | 22.79 | 4.41 | 2.14 |
| 123 | rs10447140 | intron 2 | 35136935 | C/T | T | 15.44 | 9.23 | 22.73 | 3.17 | 0.76 |
| 124 | rs35614689 | intron 2 | 35134877 | A/G | A | 20.71 | 23.91 | 2.14 | 30.71 | 53.57 |
| 125 | rs249534 | intron 2 | 35134428 | A/G | A | 15.22 | 9.56 | 20.71 | 3.68 | 2.14 |
| 126 | rs249535 | intron 2 | 35133439 | C/T | T | 16.92 | 10.29 | 19.84 | 4.48 | 2.21 |
| 127 | rs388541 | intron 2 | 35130581 | A/G | A | 47.86 | 26.81 | 27.86 | 10.14 | 10.71 |
| 128 | rs6881382 | intron 2 | 35130051 | C/T | T | 6.43 | 0.00 | 0.00 | 0.00 | 0.00 |
| 129 | rs37382^d^ | intron 2 | 35128825 | A/G | G | 58.96 | 27.27 | 25.38 | 4.69 | 8.82 |
| 130 | rs37383 | intron 2 | 35128726 | C/T | C | 61.43 | 27.54 | 27.54 | 10.87 | 11.59 |
| 131 | A+155G | intron 3 | 35125334 | A/G | A | 5.71 | 0.72 | 0.00 | 0.00 | 0.00 |
| 132 | rs249536 | intron 3 | 35124779 | A/G | G | 61.76 | 26.87 | 27.86 | 10.14 | 10.71 |
| 133 | rs17248083 | intron 3 | 35124590 | C/T | C | 15.71 | 23.91 | 2.17 | 29.71 | 52.90 |
| 134 | rs37385 | intron 3 | 35123558 | C/T | T | 58.09 | 27.61 | 26.87 | 10.71 | 11.59 |
| 135 | rs37386 | intron 3 | 35123481 | A/G | G | 58.21 | 27.21 | 27.34 | 9.09 | 10.87 |
| 136 | rs43215^d^ | intron 3 | 35122726 | C/T | T | 37.14 | 17.39 | 31.43 | 8.70 | 12.14 |
| 137 | rs12655058^d^ | intron 3 | 35122615 | A/G | A | 1.43 | 1.45 | 0.00 | 4.29 | 6.43 |
| 138 | G+3068T | intron 3 | 35122421 | G/T | G | 6.43 | 0.74 | 0.00 | 0.00 | 0.00 |
| 139 | rs37387^d^ | intron 4 | 35121357 | C/T | T | 34.29 | 16.91 | 30.71 | 10.14 | 13.57 |
| 140 | rs37388 | intron 4 | 35121319 | C/T | T | 27.21 | 16.42 | 30.43 | 9.42 | 13.04 |
| 141 | rs37389 | intron 4 | 35120937 | A/G | A | 40.71 | 17.39 | 30.43 | 9.29 | 12.86 |
| 142 | A+4834G | intron 4 | 35120655 | A/G | G | 4.48 | 0.00 | 0.00 | 0.00 | 0.00 |
| 143 | C+4884T | intron 4 | 35120605 | C/T | T | 2.14 | 0.72 | 0.00 | 0.00 | 0.00 |
| 144 | rs16871473^d, f^ | exon 5 | 35120404 | C/T | C | 10.87 | 14.71 | 5.07 | 1.45 | 2.21 |
| 145 | C+5284T | intron 5 | 35120205 | C/T | T | 5.22 | 0.00 | 0.00 | 0.00 | 0.00 |
| 146 | rs7714144 | intron 5 | 35111110 | C/T | T | 20.29 | 4.62 | 4.29 | 2.24 | 4.48 |
| 147 | A+17112G^d^ | intron 6 | 35108377 | A/G | G | 22.86 | 7.25 | 4.29 | 2.14 | 2.86 |
| 148 | rs37364^d^ | intron 6 | 35108137 | G/T | G | 65.22 | 31.16 | 33.58 | 14.93 | 24.26 |
| 149 | rs3776605^d^ | intron 7 | 35105638 | C/T | T | 1.43 | 0.00 | 5.00 | 0.00 | 0.00 |
| 150 | rs37367 | intron 9 | 35102917 | C/T | T | 40.44 | 23.13 | 30.43 | 13.57 | 20.00 |
| 151 | rs1057828^d^ | intron 10 | 35100272 | A/C | A | 9.56 | 3.03 | 2.24 | 1.47 | 2.14 |
| 152 | rs1057829 | intron 10 | 35100217 | C/G | C | 23.57 | 7.35 | 4.29 | 2.14 | 3.57 |
| 153 | rs112461^d^ | intron 10 | 35099049 | A/T | T | 25.38 | 23.53 | 29.85 | 13.24 | 19.57 |
| 154 | rs401694^d^ | intron 10 | 35098375 | C/T | T | 50.71 | 27.54 | 30.71 | 12.86 | 19.57 |
| 155 | rs387032 | intron 10 | 35097488 | C/T | C | 34.78 | 28.03 | 30.6 | 8.82 | 16.18 |
| 156 | rs1549623^d^ | intron 10 | 35096599 | A/G | A | 30.43 | 0.74 | 0.00 | 2.14 | 0.00 |
| 157 | rs392279^d^ | intron 10 | 35096488 | C/T | C | 33.33 | 27.94 | 31.43 | 9.29 | 16.67 |
| 158 | rs249521 | intron 10 | 35096270 | A/G | G | 35.71 | 28.26 | 31.43 | 9.29 | 16.67 |
| 159 | rs173627 | intron 10 | 35096258 | C/T | T | 31.34 | 26.87 | 28.03 | 7.97 | 16.67 |
| 160 | rs249522 | intron 10 | 35096076 | C/T | T | 32.86 | 27.21 | 31.88 | 9.29 | 16.43 |
| 161 | rs371913^d^ | intron 10 | 35094931 | A/G | A | 31.88 | 23.91 | 30.71 | 6.52 | 6.52 |
| 162 | rs10941232 | intron 10 | 35090396 | A/G | A | 64.29 | 34.06 | 15.94 | 56.52 | 66.43 |
| 163 | rs2217286 | intron 10 | 35090090 | C/T | T | 21.74 | 5.80 | 0.71 | 10.00 | 13.24 |
| 164 | rs11742793 | intron 10 | 35088531 | C/T | C | 1.45 | 5.80 | 2.86 | 2.86 | 5.00 |
| 165 | rs1587059 | intron 10 | 35086265 | C/G | G | 54.35 | 22.46 | 15.22 | 38.57 | 33.57 |
| 166 | rs40529 | 3' | 35084329 | C/G | C | 45.52 | 7.35 | 18.84 | 8.09 | 9.56 |
| 167 | rs187490^d^ | 3' | 35080884 | A/G | G | 40.15 | 32.31 | 29.29 | 22.79 | 36.72 |
| 168 | rs37373^d^ | 3' | 35077915 | C/T | C | 31.88 | 32.61 | 30.71 | 22.46 | 36.76 |
| 169 | rs37372 | 3' | 35077077 | C/T | T | 31.75 | 31.82 | 31.75 | 23.85 | 35.83 |
| 170 | rs2270557^d^ | 3' | 35076678 | A/G | A | 12.86 | 3.62 | 12.86 | 3.57 | 0.00 |
| 171 | rs344151^d^ | 3' | 35075807 | C/T | C | 15.94 | 0.72 | 0.00 | 2.14 | 0.00 |
| 172 | rs37370^d^ | 3' | 35075243 | C/T | C | 0.82 | 25.45 | 35.11 | 25.51 | 4.39 |
| 173 | rs2279651 | 3' | 35075194 | A/G | G | 47.10 | 33.33 | 27.86 | 40.71 | 55.15 |

^a^ SNP position based on March 2006 University of California, Santa Cruz, version human genome 18, chromosome 5 (http://genome.ucsc.edu).

^b^ Based on the minor allele frequency among all groups combined.

^c^ AA, African Americans; NH, Native Hawaiians; JA, Japanese Americans; LA, Latinas; WH, Whites.

^d^ TagSNP.

^e^ TagSNP was not genotyped in the breast cancer case-control study.

^f^ Missense SNP Ile^100^Val.

Boxes indicate block 1 (SNPs 6-30), block 2 (SNPs 31-39), block 3 (41-66), block 4 (73-88), block 5 (95-113), block 6 (114-135), block 7 (136-153), block 8 (154-161), and block 9 (167-173).

**Table S3. Descriptive characteristics of cases and controls by race/ethnicity**

|  | **AA** | **NH** | **JA** | **LA** | **WH** |
| --- | --- | --- | --- | --- | --- |
|  | Cases/Controls | Cases/Controls | Cases/Controls | Cases/Controls | Cases/Controls |
|  |  |  |  |  |  |
| n | 345/426 | 109/290 | 425/420 | 335/386 | 401/440 |
|  |  |  |  |  |  |
| Age (mean) | 64.9/64.8 | 61.1/59.6 | 64.6/64.1 | 64.2/63.8 | 65.0/62.1 |
|  |  |  |  |  |  |
| Menopausal Status^a^ |  |  |  |  |  |
| Premenopausal | 13.0/10.3 | 19.3/26.2 | 12.2/19.5 | 9.3/8.8 | 7.2/20.0 |
| Postmenopausal^b^ | 61.2/57.5 | 59.6/55.9 | 74.1/66.4 | 68.1/68.1 | 72.8/62.7 |
| Hysterectomy | 24.1/30.5 | 18.4/16.9 | 11.1/11.9 | 17.9/19.7 | 19.0/17.1 |
|  |  |  |  |  |  |
| HRT use ^a^ |  |  |  |  |  |
| Never | 52.1/50.2 | 41.5/38.9 | 26.7/31.2 | 45.6/48.7 | 26.0/35.1 |
| Ever | 47.9/48.6 | 58.5/61.1 | 73.0/67.7 | 53.1/47.9 | 74.0/64.9 |
|  |  |  |  |  |  |
| Age at menarche^a^ |  |  |  |  |  |
| ≤ 12 | 54.2/43.9 | 52.3/59.0 | 55.5/51.0 | 47.2/46.4 | 54.4/48.9 |
| 13-14 | 34.8/41.8 | 30.3/29.0 | 32.2/34.5 | 38.2/39.4 | 35.9/43.4 |
| 15+ | 9.9/12.9 | 13.8/11.4 | 9.9/13.6 | 12.2/12.7 | 8.7/7.7 |
|  |  |  |  |  |  |
| Parity^a^ |  |  |  |  |  |
| 0 | 12.8/12.7 | 10.1/8.6 | 15.5/10.7 | 10.2/7.5 | 19.2/15.0 |
| 1 | 18.8/14.3 | 5.5/9.3 | 10.8/10.0 | 8.1/5.7 | 11.7/9.3 |
| 2-3 | 38.3/40.4 | 42.2/38.3 | 55.3/60.0 | 35.8/35.5 | 49.1/53.4 |
| 4+ | 27.3/31.0 | 41.3/43.8 | 17.2/18.1 | 45.4/50.0 | 19.0/21.6 |
|  |  |  |  |  |  |
| Age at 1^st^ birth^ac^ |  |  |  |  |  |
| <20 | 44.0/52.3 | 43.9/40.4 | 8.2/11.5 | 37.8/41.0 | 23.6/23.3 |
| 21-30 | 45.6/39.1 | 49.0/49.1 | 75.8/74.9 | 51.2/51.4 | 64.6/62.6 |
| 31+ | 7.7/5.7 | 2.0/6.4 | 12.7/11.5 | 8.0/4.8 | 10.6/12.3 |
|  |  |  |  |  |  |
| Family history of breast cancer (%) | |  |  |  |  |
| Yes | 20.3/12.7 | 18.4/14.1 | 16.7/10.7 | 14.6/9.8 | 16.2/9.1 |
|  |  |  |  |  |  |
| BMI^ab^ |  |  |  |  |  |
| <23 | 11.0/11.7 | 14.7/17.6 | 42.1/45.7 | 13.1/15.3 | 31.4/30.2 |
| 23-24.9 | 15.4/8.9 | 19.3/17.6 | 22.1/20.2 | 17.6/17.6 | 23.9/21.8 |
| 25-29.9 | 36.5/36.9 | 33.9/29.0 | 28.7/26.9 | 36.1/40.2 | 26.9/29.6 |
| 30-34.9 | 21.5/25.8 | 14.7/18.3 | 4.7/5.2 | 22.1/16.8 | 11.7/10.5 |
| 35+ | 13.6/14.8 | 17.4/16.2 | 0.9/1.2 | 10.8/8.8 | 5.7/8.0 |
|  |  |  |  |  |  |
| Alcohol consumption (drinks/day)^a^ | |  |  |  |  |
| 0 | 51.0/53.3 | 63.3/56.6 | 69.9/75.2 | 54.6/51.8 | 30.9/40.0 |
| <1 | 29.6/29.1 | 20.2/30.0 | 21.2/16.9 | 30.8/33.4 | 38.2/39.6 |
| 1+ | 9.6/10.1 | 11.0/9.3 | 4.7/3.1 | 5.7/5.4 | 24.4/18.2 |

AA, African Americans; NH, Native Hawaiians; JA, Japanese Americans; LA, Latinas, WH, Whites.

^a^  Numbers do not add up to 100% because of missing data.

^b^ Women reporting natural menopause or having had a bilateral oophorectomy.

^c^ Among parous women.

**Table S4. Associations between prolactin (PRL) tagSNPs and breast cancer risk**

|  |  |  | AA |  | NH |  | JA |  | LA |  | WH |  |  |
| --- | --- | --- | --- | --- | --- | --- | --- | --- | --- | --- | --- | --- | --- |
| SNP | SNP Name | Genotype | %  cases | % controls | %  cases | % controls | %  cases | % controls | %  cases | % controls | %  cases | % controls | OR (95%CI)^a^ |
|  |  |  |  |  |  |  |  |  |  |  |  |  |  |
| Block 1 | |  |  |  |  |  |  |  |  |  |  |  |  |
| SNP 1 | rs1205945 | CC | 83.72 | 78.30 | 96.23 | 95.8 | 91.92 | 92.77 | 92.77 | 95.06 | 86.08 | 85.45 | ref |
|  |  | CT | 15.70 | 20.99 | 3.77 | 3.85 | 8.08 | 6.99 | 6.93 | 4.94 | 13.92 | 13.63 | 0.96 (0.77-1.19) |
|  |  | TT | 0.58 | 0.71 | 0.00 | 0.35 | 0.00 | 0.24 | 0.30 | 0.00 | 0.00 | 0.92 | 0.38 (0.10-1.44) |
|  |  |  |  |  |  |  |  |  |  |  |  |  | *P trend =0.392* |
|  |  |  |  |  |  |  |  |  |  |  |  |  |  |
| SNP 2 | rs2744098 | GG | 49.06 | 52.04 | 62.24 | 63.86 | 52.03 | 61.06 | 59.02 | 56.17 | 77.72 | 74.82 | ref |
|  |  | GT | 43.75 | 40.05 | 35.71 | 32.28 | 41.29 | 32.21 | 37.00 | 39.11 | 20.76 | 23.06 | 1.11 (0.95-1.28) |
|  |  | TT | 7.19 | 7.91 | 2.04 | 3.86 | 6.68 | 6.73 | 3.98 | 4.72 | 1.52 | 2.12 | 0.91 (0.66-1.25) |
|  |  |  |  |  |  |  |  |  |  |  |  |  | *P trend =0.581* |
|  |  |  |  |  |  |  |  |  |  |  |  |  |  |
| SNP 4 | rs2655419 | GG | 61.36 | 60.19 | 63.16 | 64.11 | 53.17 | 61.06 | 61.4 | 58.27 | 77.27 | 76.21 | ref |
|  |  | AG | 34.92 | 35.07 | 32.63 | 31.36 | 40.49 | 31.97 | 34.65 | 37.27 | 21.12 | 21.48 | 0.87 (0.61-1.22) |
|  |  | AA | 3.73 | 4.74 | 4.21 | 4.53 | 6.34 | 6.97 | 3.95 | 4.46 | 1.60 | 2.31 | 1.07 (0.92-1.24) |
|  |  |  |  |  |  |  |  |  |  |  |  |  | *P trend =0.902* |
|  |  |  |  |  |  |  |  |  |  |  |  |  |  |
| SNP 6 | rs9358533 | CC | 51.52 | 49.14 | 61.86 | 55.88 | 50.87 | 46.77 | 69.75 | 71.8 | 79.35 | 82.83 | ref |
|  |  | CT | 40.91 | 42.51 | 36.08 | 38.60 | 38.46 | 42.12 | 27.78 | 26.89 | 19.90 | 16.47 | 0.97 (0.84-1.13) |
|  |  | TT | 7.58 | 8.35 | 2.06 | 5.51 | 10.67 | 11.11 | 2.47 | 1.31 | 0.76 | 0.70 | 0.93 (0.68-1.27) |
|  |  |  |  |  |  |  |  |  |  |  |  |  | *P trend =0.595* |
|  |  |  |  |  |  |  |  |  |  |  |  |  |  |
| SNP 9 | rs10946546 | CC | 76.38 | 77.31 | 34.74 | 36.05 | 44.14 | 43.37 | 48.28 | 45.99 | 36.60 | 34.86 | ref |
|  |  | CT | 22.09 | 21.45 | 45.26 | 46.90 | 46.38 | 44.90 | 41.38 | 41.98 | 48.45 | 50.48 | 0.97 (0.83-1.13) |
|  |  | TT | 1.53 | 1.25 | 20.00 | 17.05 | 9.48 | 11.73 | 10.34 | 12.03 | 14.95 | 14.66 | 0.93 (0.73-1.18) |
|  |  |  |  |  |  |  |  |  |  |  |  |  | *P trend =0.519* |
|  |  |  |  |  |  |  |  |  |  |  |  |  |  |
| SNP 23 | rs2473122 | GG | 59.23 | 59.90 | 72.16 | 67.75 | 59.52 | 66.01 | 59.38 | 58.07 | 78.43 | 79.45 | ref |
|  |  | AG | 34.52 | 35.08 | 24.74 | 28.99 | 35.66 | 29.58 | 36.31 | 38.02 | 20.30 | 18.48 | 1.06 (0.91-1.23) |
|  |  | AA | 6.25 | 5.01 | 3.09 | 3.26 | 4.82 | 4.40 | 4.31 | 3.91 | 1.27 | 2.08 | 1.09 (0.77-1.55) |
|  |  |  |  |  |  |  |  |  |  |  |  |  | *P trend =0.395* |
| Block 2 | |  |  |  |  |  |  |  |  |  |  |  |  |
| SNP 25 | rs767938 | CC | 22.43 | 23.47 | 49.43 | 47.04 | 47.33 | 53.37 | 23.17 | 25.41 | 27.49 | 26.57 | ref |
|  |  | CT | 47.66 | 46.45 | 35.63 | 44.27 | 44.02 | 36.79 | 50.79 | 47.24 | 51.31 | 51.21 | 1.11 (0.95-1.31) |
|  |  | TT | 29.91 | 30.07 | 14.94 | 8.70 | 8.65 | 9.84 | 26.03 | 27.35 | 21.20 | 22.22 | 1.05 (0.85-1.28) |
|  |  |  |  |  |  |  |  |  |  |  |  |  | *P trend = 0.546* |
|  |  |  |  |  |  |  |  |  |  |  |  |  |  |
| SNP 28 | rs9358531 | TT | 68.4 | 67.79 | 38.00 | 33.45 | 45.58 | 44.23 | 48.62 | 46.01 | 33.67 | 31.11 | ref |
|  |  | GT | 28.83 | 29.09 | 41.00 | 48.78 | 45.11 | 43.27 | 40.31 | 41.76 | 47.70 | 51.84 | 0.91 (0.79-1.06) |
|  |  | GG | 2.76 | 3.13 | 21.00 | 17.77 | 9.31 | 12.50 | 11.08 | 12.23 | 18.62 | 17.05 | 0.92 (0.73-1.16) |
|  |  |  |  |  |  |  |  |  |  |  |  |  | *P trend = 0.279* |
|  |  |  |  |  |  |  |  |  |  |  |  |  |  |
| SNP 30 | rs1156546 | AA | 34.12 | 30.5 | 42.00 | 38.19 | 32.45 | 31.08 | 48.64 | 47.91 | 61.11 | 63.22 | ref |
|  |  | AC | 48.96 | 49.65 | 42.00 | 45.49 | 48.32 | 46.75 | 41.69 | 43.46 | 34.6 | 33.33 | 0.96 (0.83-1.11) |
|  |  | CC | 16.91 | 19.86 | 16.00 | 16.32 | 19.23 | 22.17 | 9.67 | 8.64 | 4.29 | 3.45 | 0.89 (0.71-1.11) |
|  |  |  |  |  |  |  |  |  |  |  |  |  | *P trend = 0.294* |
|  |  |  |  |  |  |  |  |  |  |  |  |  |  |
| SNP 32 | rs2655426 | GG | 28.23 | 32.46 | 61.54 | 61.72 | 56.29 | 63.72 | 35.14 | 37.08 | 36.36 | 34.71 | ref |
|  |  | GT | 51.95 | 49.88 | 35.58 | 34.83 | 38.72 | 30.79 | 46.85 | 45.43 | 47.73 | 48.51 | 1.15 (0.99-1.33) |
|  |  | TT | 19.82 | 17.66 | 2.88 | 3.45 | 4.99 | 5.49 | 18.02 | 17.49 | 15.91 | 16.78 | 1.11 (0.89-1.38) |
|  |  |  |  |  |  |  |  |  |  |  |  |  | *P trend = 0.148* |
|  |  |  |  |  |  |  |  |  |  |  |  |  |  |
| SNP 33 | rs1341238 | GG | 52.85 | 52.54 | 84.69 | 78.17 | 60.44 | 66.59 | 55.86 | 56.95 | 66.75 | 66.51 | ref |
|  |  | AG | 31.65 | 35.35 | 14.29 | 18.66 | 35.19 | 28.78 | 27.16 | 26.47 | 19.07 | 17.33 | 1.06 (0.9-1.24) |
|  |  | AA | 15.51 | 12.11 | 1.02 | 3.17 | 4.37 | 4.63 | 16.98 | 16.58 | 14.18 | 16.16 | 1.04 (0.83-1.3) |
|  |  |  |  |  |  |  |  |  |  |  |  |  | *P trend = 0.584* |
|  |  |  |  |  |  |  |  |  |  |  |  |  |  |
| SNP 34 | rs9466314 | AA | 85.96 | 90.80 | 99.07 | 100.00 | 100.00 | 100.00 | 97.90 | 98.44 | 100.00 | 99.54 | ref |
|  |  | AT | 13.45 | 8.73 | 0.93 | 0.00 | 0.00 | 0.00 | 2.10 | 1.56 | 0.00 | 0.46 | 1.54 (1.02-2.34) |
|  |  | TT | 0.58 | 0.47 | 0.00 | 0.00 | 0.00 | 0.00 | 0.00 | 0.00 | 0.00 | 0.00 | 1.29 (0.18-9.22) |
|  |  |  |  |  |  |  |  |  |  |  |  |  | *P trend =0.049* |
|  |  |  |  |  |  |  |  |  |  |  |  |  |  |
| SNP 36 | rs2744117 | GG | 93.96 | 93.05 | 66.02 | 66.32 | 48.43 | 45.43 | 72.64 | 77.92 | 80.95 | 85.29 | ref |
|  |  | GT | 6.04 | 6.95 | 33.98 | 30.53 | 43.61 | 42.55 | 24.32 | 20.26 | 18.80 | 14.25 | 1.13 (0.95-1.34) |
|  |  | TT | 0.00 | 0.00 | 0.00 | 3.16 | 7.95 | 12.02 | 3.04 | 1.82 | 0.25 | 0.46 | 0.73 (0.49-1.09) |
|  |  |  |  |  |  |  |  |  |  |  |  |  | *P trend = 0.897* |
|  |  |  |  |  |  |  |  |  |  |  |  |  |  |
| SNP 38 | rs849876 | AA | 76.49 | 74.1 | 96.12 | 92.00 | 95.00 | 94.65 | 87.27 | 90.08 | 82.91 | 79.22 | ref |
|  |  | AG | 21.73 | 23.98 | 3.88 | 7.27 | 4.05 | 5.11 | 12.42 | 9.66 | 16.33 | 19.63 | 0.90 (0.73-1.10) |
|  |  | GG | 1.79 | 1.92 | 0.00 | 0.73 | 0.95 | 0.24 | 0.30 | 0.26 | 0.75 | 1.14 | 0.95 (0.46-1.94) |
|  |  |  |  |  |  |  |  |  |  |  |  |  | *P trend = 0.323* |
|  |  |  |  |  |  |  |  |  |  |  |  |  |  |
| SNP 39 | rs3756824 | GG | 97.94 | 97.6 | 76.53 | 77.34 | 50.36 | 55.28 | 78.46 | 77.45 | 93.4 | 93.74 | ref |
|  |  | CG | 2.06 | 2.40 | 21.43 | 21.94 | 42.65 | 38.82 | 20.92 | 22.28 | 6.35 | 6.03 | 1.07 (0.88-1.29) |
|  |  | CC | 0.00 | 0.00 | 2.04 | 0.72 | 6.99 | 5.90 | 0.62 | 0.27 | 0.25 | 0.23 | 1.34 (0.79-2.27) |
|  |  |  |  |  |  |  |  |  |  |  |  |  | *P trend = 0.275* |
|  |  |  |  |  |  |  |  |  |  |  |  |  |  |
| SNP 43 | rs12202764 | TT | 54.52 | 54.85 | 84.26 | 86.55 | 99.53 | 99.52 | 64.46 | 68.93 | 61.35 | 59.17 | ref |
|  |  | AT | 38.19 | 40.19 | 14.81 | 13.10 | 0.47 | 0.48 | 31.93 | 26.89 | 31.92 | 35.55 | 1.03 (0.87-1.22) |
|  |  | AA | 7.29 | 4.96 | 0.93 | 0.34 | 0.00 | 0.00 | 3.61 | 4.18 | 6.73 | 5.28 | 1.23 (0.86-1.78) |
|  |  |  |  |  |  |  |  |  |  |  |  |  | *P trend = 0.346* |
|  |  |  |  |  |  |  |  |  |  |  |  |  |  |
| SNP 44 | rs2244502 | AA | 25.87 | 25.55 | 56.6 | 53.38 | 45.76 | 52.29 | 43.56 | 45.55 | 49.48 | 48.60 | ref |
|  |  | AT | 48.90 | 50.61 | 38.68 | 40.57 | 45.28 | 39.04 | 44.48 | 43.72 | 43.26 | 41.86 | 1.06 (0.92-1.23) |
|  |  | TT | 25.24 | 23.84 | 4.72 | 6.05 | 8.96 | 8.67 | 11.96 | 10.73 | 7.25 | 9.53 | 1.03 (0.82-1.29) |
|  |  |  |  |  |  |  |  |  |  |  |  |  | *P trend = 0.575* |
| Block 3 | |  |  |  |  |  |  |  |  |  |  |  |  |
| SNP 47 | A+4346G | GG | 95.36 | 91.29 | 100.00 | 99.66 | 100.00 | 100.00 | 98.2 | 98.96 | 99.75 | 99.54 | ref |
|  |  | AG | 4.06 | 8.47 | 0.00 | 0.34 | 0.00 | 0.00 | 1.80 | 1.04 | 0.25 | 0.46 | 0.58 (0.34-1.00) |
|  |  | AA | 0.58 | 0.24 | 0.00 | 0.00 | 0.00 | 0.00 | 0.00 | 0.00 | 0.00 | 0.00 | 2.42 (0.22-26.88) |
|  |  |  |  |  |  |  |  |  |  |  |  |  | *P trend = 0.129* |
|  |  |  |  |  |  |  |  |  |  |  |  |  |  |
| SNP 51 | rs849884 | CC | 98.14 | 97.14 | 100.00 | 97.39 | 100.00 | 99.51 | 94.55 | 97.12 | 91.71 | 89.59 | ref |
|  |  | CT | 1.55 | 2.62 | 0.00 | 2.61 | 0.00 | 0.49 | 5.13 | 2.88 | 8.29 | 9.93 | 0.86 (0.59-1.24) |
|  |  | TT | 0.31 | 0.24 | 0.00 | 0.00 | 0.00 | 0.00 | 0.32 | 0.00 | 0.00 | 0.48 | 0.79 (0.13-4.77) |
|  |  |  |  |  |  |  |  |  |  |  |  |  | *P trend = 0.392* |
|  |  |  |  |  |  |  |  |  |  |  |  |  |  |
| SNP 52 | rs7759000 | TT | 56.55 | 63.08 | 84.91 | 88.73 | 99.76 | 99.52 | 67.52 | 71.35 | 60.26 | 58.88 | ref |
|  |  | CT | 36.1 | 32.27 | 12.26 | 10.92 | 0.24 | 0.48 | 29.94 | 25.46 | 35.06 | 35.28 | 1.16 (0.98-1.39) |
|  |  | CC | 7.35 | 4.65 | 2.83 | 0.35 | 0.00 | 0.00 | 2.55 | 3.18 | 4.68 | 5.84 | 1.20 (0.81-1.77) |
|  |  |  |  |  |  |  |  |  |  |  |  |  | *P trend = 0.083* |
|  |  |  |  |  |  |  |  |  |  |  |  |  |  |
| SNP 54 | rs849886 | CC | 58.79 | 56.93 | 23.71 | 18.93 | 10.92 | 8.09 | 29.41 | 29.37 | 24.42 | 27.10 | ref |
|  |  | CT | 35.46 | 37.71 | 53.61 | 56.79 | 47.64 | 43.87 | 48.92 | 49.74 | 51.69 | 51.87 | 0.98 (0.82-1.16) |
|  |  | TT | 5.75 | 5.35 | 22.68 | 24.29 | 41.44 | 48.04 | 21.67 | 20.90 | 23.90 | 21.03 | 0.92 (0.75-1.13) |
|  |  |  |  |  |  |  |  |  |  |  |  |  | *P trend = 0.450* |
|  |  |  |  |  |  |  |  |  |  |  |  |  |  |
| SNP 56 | rs1205960 | CC | 48.04 | 42.41 | 61.86 | 58.27 | 82.73 | 81.71 | 64.11 | 64.75 | 55.42 | 53.95 | ref |
|  |  | CT | 38.07 | 47.47 | 28.87 | 34.89 | 17.03 | 17.32 | 31.60 | 31.07 | 39.29 | 38.60 | 0.90 (0.78-1.05) |
|  |  | TT | 13.90 | 10.12 | 9.28 | 6.83 | 0.24 | 0.98 | 4.29 | 4.18 | 5.29 | 7.44 | 0.98 (0.73-1.33) |
|  |  |  |  |  |  |  |  |  |  |  |  |  | *P trend = 0.356* |
|  |  |  |  |  |  |  |  |  |  |  |  |  |  |
| SNP 57 | rs6239 | CC | 94.77 | 94.37 | 75.47 | 72.41 | 57.55 | 65.48 | 74.40 | 70.68 | 93.98 | 95.21 | ref |
|  |  | CT | 5.23 | 5.40 | 22.64 | 27.24 | 37.26 | 31.90 | 24.10 | 27.75 | 6.02 | 4.57 | 1.05 (0.87-1.26) |
|  |  | TT | 0.00 | 0.23 | 1.89 | 0.34 | 5.19 | 2.62 | 1.51 | 1.57 | 0.00 | 0.23 | 1.65 (0.92-2.96) |
|  |  |  |  |  |  |  |  |  |  |  |  |  | *P trend = 0.224* |
|  |  |  |  |  |  |  |  |  |  |  |  |  |  |
| SNP 58 | rs10946545 | GG | 95.06 | 94.12 | 64.42 | 61.94 | 59.29 | 55.71 | 82.34 | 85.16 | 84.00 | 87.70 | ref |
|  |  | AG | 4.94 | 5.88 | 33.65 | 34.60 | 36.19 | 35.95 | 17.07 | 14.32 | 15.75 | 12.07 | 1.06 (0.89-1.27) |
|  |  | AA | 0.00 | 0.00 | 1.92 | 3.46 | 4.52 | 8.33 | 0.60 | 0.52 | 0.25 | 0.23 | 0.57 (0.34-0.96) |
|  |  |  |  |  |  |  |  |  |  |  |  |  | *P trend = 0.559* |
|  |  |  |  |  |  |  |  |  |  |  |  |  |  |
| SNP 59 | rs1205961 | GG | 13.02 | 11.86 | 59.18 | 52.82 | 94.3 | 94.65 | 45.10 | 46.95 | 42.67 | 36.00 | ref |
|  |  | AG | 45.4 | 47.46 | 30.61 | 39.44 | 5.70 | 5.11 | 42.48 | 41.91 | 45.55 | 50.12 | 0.93 (0.74-1.18) |
|  |  | AA | 41.59 | 40.68 | 10.20 | 7.75 | 0.00 | 0.24 | 12.42 | 11.14 | 11.78 | 13.88 | 0.89 (0.75-1.06) |
|  |  |  |  |  |  |  |  |  |  |  |  |  | *P trend = 0.418* |
| Block 4 | |  |  |  |  |  |  |  |  |  |  |  |  |
| SNP 62 | rs849870 | CC | 47.02 | 46.4 | 58.59 | 54.84 | 87.68 | 88.22 | 74.38 | 73.63 | 74.94 | 73.83 | ref |
|  |  | CT | 43.05 | 44.42 | 36.36 | 34.77 | 11.11 | 11.54 | 24.69 | 23.76 | 22.76 | 24.07 | 0.98 (0.83-1.16) |
|  |  | TT | 9.93 | 9.18 | 5.05 | 10.39 | 1.21 | 0.24 | 0.93 | 2.61 | 2.30 | 2.10 | 0.88 (0.61-1.27) |
|  |  |  |  |  |  |  |  |  |  |  |  |  | *P trend = 0.559* |
|  |  |  |  |  |  |  |  |  |  |  |  |  |  |
| SNP 65 | rs849872 | TT | 45.98 | 47.6 | 45.98 | 47.6 | 36.89 | 42.03 | 52.02 | 46.17 | 67.95 | 67.98 | ref |
|  |  | CT | 43.41 | 41.83 | 43.41 | 41.83 | 46.12 | 45.65 | 40.50 | 41.42 | 28.21 | 28.31 | 1.04 (0.90-1.20) |
|  |  | CC | 10.61 | 10.58 | 10.61 | 10.58 | 16.99 | 12.32 | 7.48 | 12.40 | 3.85 | 3.71 | 0.94 (0.74-1.19) |
|  |  |  |  |  |  |  |  |  |  |  |  |  | *P trend = 0.895* |
|  |  |  |  |  |  |  |  |  |  |  |  |  |  |
| SNP 68 | rs2744119 | AA | 94.08 | 93.79 | 57.14 | 64.1 | 50.84 | 52.22 | 67.78 | 73.68 | 79.35 | 78.37 | ref |
|  |  | AG | 5.92 | 6.21 | 30.61 | 30.4 | 39.81 | 40.39 | 28.57 | 24.21 | 18.64 | 19.77 | 0.99 (0.57-1.71) |
|  |  | GG | 0.00 | 0.00 | 12.24 | 5.49 | 9.35 | 7.39 | 3.65 | 2.11 | 2.02 | 1.86 | 1.65 (0.42-6.38) |
|  |  |  |  |  |  |  |  |  |  |  |  |  | *P trend = 0.100* |
|  |  |  |  |  |  |  |  |  |  |  |  |  |  |
| SNP 69 | rs2744120 | AA | 43.17 | 42.68 | 20.88 | 32.03 | 21.55 | 18.77 | 28.25 | 28.07 | 23.44 | 23.64 | ref |
|  |  | AG | 45.34 | 43.66 | 52.75 | 45.31 | 45.11 | 46.02 | 43.17 | 52.14 | 48.44 | 48.46 | 1.28 (0.73-2.27) |
|  |  | GG | 11.49 | 13.66 | 26.37 | 22.66 | 33.33 | 35.22 | 28.57 | 19.79 | 28.13 | 27.9 | 1.10 (0.58-2.09) |
|  |  |  |  |  |  |  |  |  |  |  |  |  | *P trend = 0.663* |
|  |  |  |  |  |  |  |  |  |  |  |  |  |  |
| SNP 70 | rs12524841 | TT | 63.50 | 58.29 | 77.57 | 69.20 | 44.21 | 49.40 | 63.75 | 59.16 | 84.00 | 83.14 | ref |
|  |  | GT | 30.86 | 36.73 | 20.56 | 26.64 | 42.08 | 39.62 | 32.93 | 35.08 | 15.25 | 15.95 | 0.92 (0.79-1.07) |
|  |  | GG | 5.64 | 4.98 | 1.87 | 4.15 | 13.71 | 10.98 | 3.32 | 5.76 | 0.75 | 0.91 | 0.99 (0.73-1.33) |
|  |  |  |  |  |  |  |  |  |  |  |  |  | *P trend = 0.435* |
|  |  |  |  |  |  |  |  |  |  |  |  |  |  |
| SNP 73 | rs2153646 | TT | 49.23 | 44.53 | 52.08 | 47.14 | 32.35 | 34.31 | 49.21 | 45.84 | 66.75 | 65.65 | ref |
|  |  | AT | 40.31 | 44.77 | 41.67 | 42.50 | 45.68 | 48.66 | 42.59 | 41.82 | 28.13 | 29.88 | 1.10 (0.68-1.76) |
|  |  | AA | 10.46 | 10.71 | 6.25 | 10.36 | 21.98 | 17.03 | 8.20 | 12.33 | 5.12 | 4.47 | 0.61 (0.17-2.13) |
|  |  |  |  |  |  |  |  |  |  |  |  |  | *P trend = 0.436* |
|  |  |  |  |  |  |  |  |  |  |  |  |  |  |
| SNP 75 | rs6940783 | CC | 74.54 | 76.67 | 86.14 | 73.78 | 98.57 | 98.99 | 70.59 | 71.88 | 56.04 | 54.74 | ref |
|  |  | CG | 22.70 | 20.84 | 13.86 | 25.17 | 1.43 | 1.01 | 26.93 | 24.93 | 39.07 | 38.86 | 0.98 (0.82-1.17) |
|  |  | GG | 2.76 | 2.48 | 0.00 | 1.05 | 0.00 | 0.00 | 2.48 | 3.18 | 4.88 | 6.40 | 0.82 (0.53-1.28) |
|  |  |  |  |  |  |  |  |  |  |  |  |  | *P trend = 0.524* |
|  |  |  |  |  |  |  |  |  |  |  |  |  |  |
| SNP 77 | rs2066266 | AA | 15.63 | 18.23 | 46.53 | 39.86 | 88.78 | 88.54 | 44.07 | 42.59 | 31.54 | 30.11 | ref |
|  |  | AG | 47.79 | 44.36 | 43.56 | 43.36 | 10.5 | 11.22 | 41.34 | 44.97 | 49.23 | 48.05 | 0.99 (0.83-1.17) |
|  |  | GG | 36.58 | 37.41 | 9.90 | 16.78 | 0.72 | 0.24 | 14.59 | 12.43 | 19.23 | 21.84 | 0.94 (0.75-1.16) |
|  |  |  |  |  |  |  |  |  |  |  |  |  | *P trend = 0.581* |

AA, African Americans; NH, Native Hawaiians; JA, Japanese Americans; LA, Latinas, WH, Whites.

^a^ ORs adjusted for age and ethnicity.

^b^ P-value for test of trend.

**Table S5. Associations between prolactin receptor (PRLR) tagSNPs and breast cancer risk**

|  |  |  | AA |  | NH |  | JA |  | LA |  | WH |  |  |
| --- | --- | --- | --- | --- | --- | --- | --- | --- | --- | --- | --- | --- | --- |
| SNP | SNP Name | Genotype | %  cases | % controls | %  cases | % controls | %  cases | % controls | %  cases | % controls | %  cases | % controls | OR (95%CI)^a^ |
|  |  |  |  |  |  |  |  |  |  |  |  |  |  |
| Block 1 | |  |  |  |  |  |  |  |  |  |  |  |  |
| SNP 7 | rs11741701 | GG | 61.99 | 63.17 | 80.00 | 68.38 | 55.28 | 61.03 | 53.11 | 50.80 | 47.09 | 43.37 | ref |
|  |  | AG | 31.78 | 33.17 | 19.00 | 27.94 | 37.84 | 34.62 | 43.48 | 41.22 | 43.12 | 43.86 | 0.99 (0.86-1.15) |
|  |  | AA | 6.23 | 3.66 | 1.00 | 3.68 | 6.88 | 4.36 | 3.42 | 7.98 | 9.79 | 12.77 | 0.89 (0.67-1.18) |
|  |  |  |  |  |  |  |  |  |  |  |  |  | *P trend = 0.529* |
|  |  |  |  |  |  |  |  |  |  |  |  |  |  |
| SNP 9 | rs7726240 | TT | 60.88 | 52.52 | 89.52 | 86.22 | 78.74 | 80.78 | 92.57 | 90.74 | 91.37 | 90.63 | ref |
|  |  | CT | 35.00 | 40.77 | 10.48 | 13.78 | 19.08 | 17.27 | 6.81 | 8.99 | 8.63 | 9.13 | 0.87 (0.72-1.05) |
|  |  | CC | 4.12 | 6.71 | 0.00 | 0.00 | 2.17 | 1.95 | 0.62 | 0.26 | 0.00 | 0.23 | 0.71 (0.42-1.20) |
|  |  |  |  |  |  |  |  |  |  |  |  |  | *P trend = 0.071* |
|  |  |  |  |  |  |  |  |  |  |  |  |  |  |
| SNP 11 | rs7734446 | AA | 93.86 | 94.13 | 23.30 | 32.87 | 27.01 | 25.06 | 64.95 | 66.75 | 82.58 | 87.41 | ref |
|  |  | AG | 6.14 | 5.87 | 50.49 | 49.48 | 47.63 | 48.67 | 32.02 | 28.76 | 16.92 | 11.67 | 1.13 (0.95-1.35) |
|  |  | GG | 0.00 | 0.00 | 26.21 | 17.65 | 25.36 | 26.27 | 3.02 | 4.49 | 0.51 | 0.92 | 1.11 (0.84-1.46) |
|  |  |  |  |  |  |  |  |  |  |  |  |  | *P trend = 0.262* |
|  |  |  |  |  |  |  |  |  |  |  |  |  |  |
| SNP 16 | rs7728298 | AA | 13.91 | 12.35 | 47.12 | 43.25 | 60.85 | 61.34 | 35.05 | 36.46 | 28.72 | 26.16 | ref |
|  |  | AG | 46.75 | 48.93 | 42.31 | 45.33 | 33.96 | 31.50 | 47.73 | 48.18 | 50.13 | 48.84 | 0.98 (0.84-1.15) |
|  |  | GG | 39.35 | 38.72 | 10.58 | 11.42 | 5.19 | 7.16 | 17.22 | 15.36 | 21.16 | 25.00 | 0.92 (0.75-1.12) |
|  |  |  |  |  |  |  |  |  |  |  |  |  | *P trend = 0.424* |
|  |  |  |  |  |  |  |  |  |  |  |  |  |  |
| SNP 23 | rs4703398 | GG | 95.68 | 97.27 | 96.00 | 97.01 | 89.08 | 91.48 | 88.72 | 88.33 | 95.26 | 92.74 | ref |
|  |  | AG | 4.01 | 2.48 | 4.00 | 2.99 | 10.92 | 7.77 | 10.06 | 11.41 | 4.74 | 7.02 | 1.06 (0.81-1.39) |
|  |  | AA | 0.31 | 0.25 | 0.00 | 0.00 | 0.00 | 0.75 | 1.22 | 0.27 | 0.00 | 0.24 | 0.95 (0.29-3.15) |
|  |  |  |  |  |  |  |  |  |  |  |  |  | *P trend = 0.742* |
|  |  |  |  |  |  |  |  |  |  |  |  |  |  |
| SNP 30 | hCV11281097 | TT | 38.62 | 39.18 | 40.38 | 30.91 | 46.52 | 46.57 | 13.68 | 15.38 | 7.91 | 5.44 | ref |
|  |  | GT | 43.11 | 45.67 | 42.31 | 47.64 | 38.85 | 41.42 | 44.98 | 38.73 | 38.78 | 31.68 | 0.98 (0.83-1.17) |
|  |  | GG | 18.26 | 15.14 | 17.31 | 21.45 | 14.63 | 12.01 | 41.34 | 45.89 | 53.32 | 62.88 | 0.88 (0.72-1.07) |
|  |  |  |  |  |  |  |  |  |  |  |  |  | *P trend = 0.191* |
| Block 2 | |  |  |  |  |  |  |  |  |  |  |  |  |
| SNP 31 | rs10068521 | GG | 82.22 | 90.35 | 94.44 | 96.89 | 90.02 | 91.59 | 88.86 | 87.99 | 0.00 | 0.00 | ref |
|  |  | CG | 16.62 | 9.65 | 5.56 | 3.11 | 9.98 | 7.69 | 10.54 | 11.49 | 95.26 | 92.91 | 1.21 (0.96-1.53) |
|  |  | CC | 1.17 | 0.00 | 0.00 | 0.00 | 0.00 | 0.72 | 0.60 | 0.52 | 4.74 | 7.09 | 1.38 (0.42-4.54) |
|  |  |  |  |  |  |  |  |  |  |  |  |  | *P trend = 0.097* |
|  |  |  |  |  |  |  |  |  |  |  |  |  |  |
| SNP 32 | rs7733450 | TT | 81.82 | 77.01 | 89.81 | 87.85 | 78.44 | 80.43 | 96.12 | 96.09 | 90.75 | 89.04 | ref |
|  |  | AT | 17.60 | 20.85 | 10.19 | 11.81 | 19.43 | 17.66 | 3.28 | 3.91 | 9.25 | 10.73 | 0.93 (0.75-1.13) |
|  |  | AA | 0.59 | 2.13 | 0.00 | 0.35 | 2.13 | 1.91 | 0.60 | 0.00 | 0.00 | 0.23 | 0.74 (0.36-1.52) |
|  |  |  |  |  |  |  |  |  |  |  |  |  | *P trend = 0.303* |
|  |  |  |  |  |  |  |  |  |  |  |  |  |  |
| SNP 35 | rs6863018 | GG | 31.75 | 30.88 | 49.51 | 32.87 | 46.68 | 46.63 | 13.76 | 13.02 | 9.39 | 9.15 | ref |
|  |  | AG | 46.88 | 52.73 | 36.89 | 46.02 | 42.42 | 43.99 | 52.91 | 49.74 | 44.92 | 40.96 | 0.90 (0.76-1.07) |
|  |  | AA | 21.36 | 16.39 | 13.59 | 21.11 | 10.90 | 9.38 | 0.00 | 0.00 | 45.69 | 49.89 | 0.89 (0.72-1.08) |
|  |  |  |  |  |  |  |  |  |  |  |  |  | *P trend = 0.232* |
|  |  |  |  |  |  |  |  |  |  |  |  |  |  |
| SNP 38 | rs931740 | CC | 64.05 | 55.07 | 17.00 | 29.08 | 21.36 | 21.34 | 44.51 | 50.26 | 57.03 | 64.87 | ref |
|  |  | CT | 33.23 | 38.65 | 46.00 | 46.45 | 49.03 | 46.15 | 48.17 | 42.63 | 36.83 | 31.62 | 1.11 (0.95-1.29) |
|  |  | TT | 2.72 | 6.28 | 37.00 | 24.47 | 29.61 | 32.51 | 7.32 | 7.11 | 6.14 | 3.51 | 1.09 (0.87-1.37) |
|  |  |  |  |  |  |  |  |  |  |  |  |  | *P trend = 0.273* |
|  |  |  |  |  |  |  |  |  |  |  |  |  |  |
| SNP 39 | rs7731153 | AA | 91.76 | 90.8 | 26.92 | 34.26 | 23.22 | 20.10 | 72.81 | 74.14 | 83.80 | 88.05 | ref |
|  |  | AG | 8.24 | 9.20 | 45.19 | 47.75 | 49.53 | 49.04 | 24.77 | 23.75 | 15.70 | 11.03 | 1.07 (0.89-1.28) |
|  |  | GG | 0.00 | 0.00 | 27.88 | 17.99 | 27.25 | 30.86 | 2.42 | 2.11 | 0.51 | 0.92 | 1.04 (0.79-1.37) |
|  |  |  |  |  |  |  |  |  |  |  |  |  | *P trend = 0.625* |
| Block 3 | |  |  |  |  |  |  |  |  |  |  |  |  |
| SNP 41 | rs6451190 | CC | 91.52 | 92.00 | 29.81 | 36.59 | 19.67 | 17.94 | 65.17 | 65.63 | 83.38 | 83.79 | ref |
|  |  | CG | 8.19 | 7.53 | 49.04 | 47.39 | 50.00 | 50.72 | 31.23 | 31.25 | 15.87 | 15.75 | 1.02 (0.86-1.22) |
|  |  | GG | 0.29 | 0.47 | 21.15 | 16.03 | 30.33 | 31.34 | 3.60 | 3.13 | 0.76 | 0.46 | 1.05 (0.80-1.38) |
|  |  |  |  |  |  |  |  |  |  |  |  |  | ***P trend = 0.691*** |
|  |  |  |  |  |  |  |  |  |  |  |  |  |  |
| SNP 49 | rs34024951 | CC | 51.63 | 46.57 | 93.4 | 88.97 | 86.76 | 86.19 | 84.34 | 83.77 | 84.96 | 84.02 | ref |
|  |  | CT | 43.03 | 42.79 | 6.60 | 11.03 | 12.77 | 13.81 | 15.06 | 15.18 | 14.79 | 15.30 | 0.92 (0.77-1.10) |
|  |  | TT | 5.34 | 10.64 | 0.00 | 0.00 | 0.47 | 0.00 | 0.60 | 1.05 | 0.25 | 0.68 | 0.50 (0.30-0.83) |
|  |  |  |  |  |  |  |  |  |  |  |  |  | ***P trend =* *0.032*** |
|  |  |  |  |  |  |  |  |  |  |  |  |  |  |
| SNP 51 | rs4703510 | TT | 48.00 | 44.39 | 29.70 | 31.02 | 13.46 | 12.38 | 54.46 | 52.91 | 69.47 | 70.33 | ref |
|  |  | AT | 46.46 | 45.37 | 49.50 | 47.08 | 48.56 | 48.02 | 37.85 | 38.62 | 27.99 | 26.17 | 0.98 (0.84-1.14) |
|  |  | AA | 5.54 | 10.24 | 20.79 | 21.90 | 37.98 | 39.60 | 7.69 | 8.47 | 2.54 | 3.50 | 0.83 (0.66-1.04) |
|  |  |  |  |  |  |  |  |  |  |  |  |  | *P trend =* *0.157* |
|  |  |  |  |  |  |  |  |  |  |  |  |  |  |
| SNP 52 | rs4235652 | TT | 45.27 | 44.00 | 26.42 | 30.90 | 13.66 | 12.17 | 54.35 | 53.93 | 69.77 | 69.25 | ref |
|  |  | CT | 48.65 | 45.65 | 48.11 | 47.92 | 47.56 | 47.97 | 38.44 | 37.7 | 27.71 | 27.33 | 1.00 (0.85-1.16) |
|  |  | CC | 6.08 | 10.35 | 25.47 | 21.18 | 38.78 | 39.86 | 7.21 | 8.38 | 2.52 | 3.42 | 0.89 (0.71-1.12) |
|  |  |  |  |  |  |  |  |  |  |  |  |  | *P trend =* *0.422* |
|  |  |  |  |  |  |  |  |  |  |  |  |  |  |
| SNP 53 | rs2047740 | GG | 25.89 | 35.90 | 46.53 | 36.04 | 45.56 | 45.65 | 18.48 | 16.76 | 12.82 | 10.77 | ref |
|  |  | AG | 53.87 | 46.75 | 40.59 | 43.11 | 43.65 | 44.69 | 50.30 | 51.06 | 43.33 | 42.86 | 1.04 (0.88-1.22) |
|  |  | AA | 20.24 | 17.35 | 12.87 | 20.85 | 10.79 | 9.66 | 31.21 | 32.18 | 43.85 | 46.37 | 1.00 (0.82-1.22) |
|  |  |  |  |  |  |  |  |  |  |  |  |  | *P trend =* *0.989* |
|  |  |  |  |  |  |  |  |  |  |  |  |  |  |
| SNP 55 | rs875701 | AA | 22.16 | 31.43 | 46.15 | 35.31 | 43.23 | 45.32 | 18.37 | 16.67 | 10.89 | 9.66 | ref |
|  |  | AG | 52.99 | 48.81 | 40.38 | 44.41 | 45.61 | 45.56 | 50.90 | 50.52 | 44.30 | 44.60 | 1.05 (0.89-1.24) |
|  |  | GG | 24.85 | 19.76 | 13.46 | 20.28 | 11.16 | 9.11 | 30.72 | 32.81 | 44.81 | 45.75 | 1.07 (0.87-1.3) |
|  |  |  |  |  |  |  |  |  |  |  |  |  | *P trend =* *0.535* |
|  |  |  |  |  |  |  |  |  |  |  |  |  |  |
| SNP 57 | rs4703509 | TT | 33.53 | 28.64 | 60.58 | 67.74 | 78.69 | 77.88 | 57.14 | 57.84 | 56.15 | 58.29 | ref |
|  |  | CT | 52.40 | 47.02 | 33.65 | 29.75 | 19.61 | 21.39 | 37.27 | 37.84 | 36.67 | 36.49 | 1.03 (0.88-1.19) |
|  |  | CC | 14.07 | 24.34 | 5.77 | 2.51 | 1.69 | 0.72 | 5.59 | 4.32 | 7.18 | 5.21 | 0.86 (0.65-1.14) |
|  |  |  |  |  |  |  |  |  |  |  |  |  | *P trend =* *0.596* |
|  |  |  |  |  |  |  |  |  |  |  |  |  |  |
| SNP 60 | rs1587607 | AA | 50.45 | 42.72 | 75.25 | 78.52 | 75.06 | 72.18 | 67.69 | 69.03 | 59.24 | 62.85 | ref |
|  |  | AG | 42.99 | 43.68 | 22.77 | 19.72 | 23.26 | 26.38 | 29.23 | 28.87 | 34.43 | 33.41 | 0.97 (0.83-1.12) |
|  |  | GG | 6.57 | 13.6 | 1.98 | 1.76 | 1.68 | 1.44 | 3.08 | 2.10 | 6.33 | 3.74 | 0.84 (0.60-1.18) |
|  |  |  |  |  |  |  |  |  |  |  |  |  | *P trend =* *0.367* |
|  |  |  |  |  |  |  |  |  |  |  |  |  |  |
| SNP 63 | rs7705216 | CC | 91.55 | 88.21 | 74.53 | 75.09 | 97.88 | 96.9 | 79.82 | 84.64 | 72.15 | 76.89 | ref |
|  |  | CG | 8.16 | 10.85 | 23.58 | 20.76 | 2.12 | 3.10 | 18.98 | 15.10 | 26.08 | 22.2 | 1.11 (0.91-1.36) |
|  |  | GG | 0.29 | 0.94 | 1.89 | 4.15 | 0.00 | 0.00 | 1.20 | 0.26 | 1.77 | 0.92 | 1.05 (0.52-2.12) |
|  |  |  |  |  |  |  |  |  |  |  |  |  | *P trend =* *0.326* |
| Block 4 | |  |  |  |  |  |  |  |  |  |  |  |  |
| SNP 73 | rs10941235 | CC | 38.12 | 31.75 | 40.38 | 44.64 | 67.7 | 65.31 | 55.05 | 54.83 | 51.25 | 53.20 | ref |
|  |  | CT | 46.63 | 50.00 | 50.96 | 44.98 | 28.71 | 31.58 | 37.61 | 39.43 | 40.00 | 40.41 | 0.94 (0.82-1.09) |
|  |  | TT | 15.25 | 18.25 | 8.65 | 10.38 | 3.59 | 3.11 | 7.34 | 5.74 | 8.75 | 6.39 | 0.98 (0.76-1.26) |
|  |  |  |  |  |  |  |  |  |  |  |  |  | *P trend =* *0.590* |
|  |  |  |  |  |  |  |  |  |  |  |  |  |  |
| SNP 75 | rs7700286 | TT | 80.70 | 79.10 | 75.24 | 76.14 | 74.05 | 70.94 | 84.98 | 85.16 | 85.14 | 86.51 | ref |
|  |  | CT | 18.71 | 19.95 | 20.95 | 21.21 | 23.57 | 26.39 | 15.02 | 14.32 | 14.11 | 12.79 | 0.96 (0.81-1.14) |
|  |  | CC | 0.58 | 0.95 | 3.81 | 2.65 | 2.38 | 2.66 | 0.00 | 0.52 | 0.76 | 0.70 | 0.84 (0.46-1.54) |
|  |  |  |  |  |  |  |  |  |  |  |  |  | *P trend =* *0.505* |
|  |  |  |  |  |  |  |  |  |  |  |  |  |  |
| SNP 77 | rs10067710 | AA | 78.65 | 79.48 | 78.30 | 69.90 | 99.06 | 97.62 | 50.76 | 55.5 | 39.75 | 41.06 | ref |
|  |  | AT | 20.18 | 19.81 | 19.81 | 25.95 | 0.94 | 2.38 | 39.58 | 36.91 | 47.85 | 45.41 | 1.03 (0.87-1.22) |
|  |  | TT | 1.17 | 0.71 | 1.89 | 4.15 | 0.00 | 0.00 | 9.67 | 7.59 | 12.41 | 13.53 | 1.02 (0.74-1.39) |
|  |  |  |  |  |  |  |  |  |  |  |  |  | *P trend =* *0.801* |
|  |  |  |  |  |  |  |  |  |  |  |  |  |  |
| SNP 78 | rs34212252 | GG | 66.07 | 59.9 | 77.78 | 75.54 | 73.32 | 71.39 | 83.69 | 80.81 | 81.75 | 80.90 | ref |
|  |  | CG | 29.46 | 36.23 | 19.19 | 21.58 | 23.8 | 25.87 | 16.00 | 18.38 | 17.22 | 18.16 | 0.85 (0.72-1.00) |
|  |  | CC | 4.46 | 3.86 | 3.03 | 2.88 | 2.88 | 2.74 | 0.31 | 0.81 | 1.03 | 0.94 | 0.98 (0.61-1.56) |
|  |  |  |  |  |  |  |  |  |  |  |  |  | *P trend =* *0.101* |
|  |  |  |  |  |  |  |  |  |  |  |  |  |  |
| SNP 79 | rs6895193 | GG | 43.20 | 41.23 | 27.88 | 34.26 | 10.66 | 9.31 | 35.35 | 30.03 | 43.29 | 42.30 | ref |
|  |  | AG | 45.86 | 47.63 | 52.88 | 48.44 | 41.47 | 42.72 | 47.73 | 49.61 | 46.08 | 44.60 | 0.94 (0.81-1.10) |
|  |  | AA | 10.95 | 11.14 | 19.23 | 17.30 | 47.87 | 47.97 | 16.92 | 20.37 | 10.63 | 13.10 | 0.89 (0.73-1.09) |
|  |  |  |  |  |  |  |  |  |  |  |  |  | *P trend =* *0.271* |
|  |  |  |  |  |  |  |  |  |  |  |  |  |  |
| SNP 84 | rs12518881 | GG | 87.35 | 80.1 | 70.48 | 73.68 | 97.87 | 97.36 | 79.28 | 84.03 | 71.03 | 76.64 | ref |
|  |  | AG | 12.06 | 19.18 | 26.67 | 23.16 | 2.13 | 2.64 | 19.52 | 15.71 | 27.2 | 22.66 | 1.06 (0.88-1.28) |
|  |  | AA | 0.59 | 0.72 | 2.86 | 3.16 | 0.00 | 0.00 | 1.20 | 0.26 | 1.76 | 0.70 | 1.46 (0.71-3.01) |
|  |  |  |  |  |  |  |  |  |  |  |  |  | *P trend =* *0.333* |
|  |  |  |  |  |  |  |  |  |  |  |  |  |  |
| SNP 86 | rs1039427 | CC | 52.73 | 42.75 | 38.14 | 41.82 | 68.30 | 65.5 | 57.28 | 55.11 | 51.03 | 52.73 | ref |
|  |  | CT | 37.88 | 46.38 | 45.36 | 44.36 | 28.01 | 31.5 | 36.53 | 40.59 | 39.69 | 39.43 | 0.87 (0.75-1.01) |
|  |  | TT | 9.39 | 10.87 | 16.49 | 13.82 | 3.69 | 3.00 | 6.19 | 4.30 | 9.28 | 7.84 | 1.05 (0.80-1.37) |
|  |  |  |  |  |  |  |  |  |  |  |  |  | *P trend =0.395* |
| Block 5 | |  |  |  |  |  |  |  |  |  |  |  |  |
| SNP 98 | rs7732013 | CC | 46.2 | 43.97 | 84.91 | 84.51 | 91.49 | 90.71 | 88.55 | 87.93 | 90.98 | 93.36 | ref |
|  |  | CT | 40.94 | 44.92 | 14.15 | 14.79 | 8.27 | 9.29 | 10.54 | 12.07 | 8.77 | 6.18 | 0.95 (0.78-1.16) |
|  |  | TT | 12.87 | 11.11 | 0.94 | 0.70 | 0.24 | 0.00 | 0.90 | 0.00 | 0.25 | 0.46 | 1.22 (0.80-1.86) |
|  |  |  |  |  |  |  |  |  |  |  |  |  | *P trend =* *0.845* |
|  |  |  |  |  |  |  |  |  |  |  |  |  |  |
| SNP 99 | rs2047741 | CC | 33.33 | 32.94 | 65.38 | 74.11 | 58.65 | 62.92 | 84.24 | 80.63 | 83.92 | 89.15 | ref |
|  |  | CG | 47.79 | 45.73 | 31.73 | 22.70 | 38.46 | 31.82 | 14.85 | 18.32 | 14.82 | 9.24 | 1.18 (1.00-1.39) |
|  |  | GG | 18.88 | 21.33 | 2.88 | 3.19 | 2.88 | 5.26 | 0.91 | 1.05 | 1.26 | 1.62 | 0.85 (0.63-1.15) |
|  |  |  |  |  |  |  |  |  |  |  |  |  | *P trend =* *0.650* |
|  |  |  |  |  |  |  |  |  |  |  |  |  |  |
| SNP 102 | rs1587605 | TT | 73.29 | 69.98 | 77.42 | 85.71 | 67.38 | 69.30 | 89.46 | 88.05 | 90.21 | 90.57 | ref |
|  |  | CT | 24.32 | 27.19 | 22.58 | 12.20 | 30.73 | 27.34 | 10.54 | 11.43 | 9.26 | 9.20 | 1.06 (0.88-1.26) |
|  |  | CC | 2.40 | 2.84 | 0.00 | 2.09 | 1.89 | 3.36 | 0.00 | 0.52 | 0.53 | 0.23 | 0.60 (0.33-1.09) |
|  |  |  |  |  |  |  |  |  |  |  |  |  | *P trend =* *0.723* |
|  |  |  |  |  |  |  |  |  |  |  |  |  |  |
| SNP 106 | rs3797212 | CC | 81.71 | 79.71 | 71.7 | 75.27 | 90.74 | 91.39 | 95.15 | 91.93 | 92.95 | 95.78 | ref |
|  |  | CT | 16.81 | 18.62 | 26.42 | 22.61 | 9.03 | 8.61 | 4.55 | 8.07 | 7.05 | 3.75 | 1.00 (0.81-1.25) |
|  |  | TT | 1.47 | 1.67 | 1.89 | 2.12 | 0.24 | 0.00 | 0.30 | 0.00 | 0.00 | 0.47 | 0.92 (0.39-2.15) |
|  |  |  |  |  |  |  |  |  |  |  |  |  | *P trend =* *0.958* |
|  |  |  |  |  |  |  |  |  |  |  |  |  |  |
| SNP 111 | rs2962086 | AA | 35.88 | 38.86 | 77.78 | 84.14 | 67.85 | 69.62 | 87.13 | 86.23 | 88.72 | 90.18 | ref |
|  |  | AG | 45.00 | 46.21 | 22.22 | 13.45 | 30.5 | 27.27 | 12.28 | 12.99 | 10.78 | 9.59 | 1.11 (0.94-1.32) |
|  |  | GG | 19.12 | 14.93 | 0.00 | 2.41 | 1.65 | 3.11 | 0.60 | 0.78 | 0.50 | 0.23 | 1.15 (0.82-1.62) |
|  |  |  |  |  |  |  |  |  |  |  |  |  | *P trend =* *0.201* |
|  |  |  |  |  |  |  |  |  |  |  |  |  |  |
| SNP 112 | rs930068 | TT | 86.05 | 85.88 | 97.22 | 98.96 | 100.00 | 99.76 | 94.93 | 94.29 | 92.52 | 93.15 | ref |
|  |  | GT | 13.66 | 12.94 | 2.78 | 1.04 | 0.00 | 0.24 | 5.07 | 5.45 | 6.98 | 6.62 | 1.05 (0.79-1.41) |
|  |  | GG | 0.29 | 1.18 | 0.00 | 0.00 | 0.00 | 0.00 | 0.00 | 0.26 | 0.50 | 0.23 | 0.46 (0.12-1.78) |
|  |  |  |  |  |  |  |  |  |  |  |  |  | *P trend =* *0.881* |
| Block 6 | |  |  |  |  |  |  |  |  |  |  |  |  |
| SNP 114 | rs35322400 | TT | 56.97 | 54.46 | 78.00 | 84.70 | 58.5 | 63.18 | 86.75 | 86.63 | 89.37 | 89.30 | ref |
|  |  | CT | 38.48 | 36.63 | 22.00 | 13.17 | 38.35 | 32.09 | 13.25 | 12.83 | 9.87 | 10.47 | 1.14 (0.96-1.35) |
|  |  | CC | 4.55 | 8.92 | 0.00 | 2.14 | 3.16 | 4.73 | 0.00 | 0.53 | 0.76 | 0.23 | 0.56 (0.36-0.88) |
|  |  |  |  |  |  |  |  |  |  |  |  |  | *P trend =* *0.785* |
|  |  |  |  |  |  |  |  |  |  |  |  |  |  |
| SNP 117 | rs1966571 | TT | 24.33 | 26.09 | 53.40 | 60.07 | 49.88 | 56.76 | 80.85 | 77.34 | 81.82 | 83.80 | ref |
|  |  | CT | 46.29 | 47.34 | 40.78 | 33.92 | 43.68 | 36.47 | 17.63 | 21.35 | 16.92 | 14.81 | 1.12 (0.96-1.32) |
|  |  | CC | 29.38 | 26.57 | 5.83 | 6.01 | 6.44 | 6.76 | 1.52 | 1.30 | 1.26 | 1.39 | 1.15 (0.88-1.50) |
|  |  |  |  |  |  |  |  |  |  |  |  |  | *P trend =* *0.147* |
|  |  |  |  |  |  |  |  |  |  |  |  |  |  |
| SNP 119 | rs7734558 | GG | 68.18 | 62.38 | 65.26 | 67.14 | 92.36 | 95.16 | 46.96 | 45.89 | 20.47 | 24.47 | ref |
|  |  | AG | 29.09 | 33.57 | 28.42 | 28.27 | 7.64 | 4.84 | 41.53 | 43.77 | 50.78 | 44.00 | 1.02 (0.86-1.21) |
|  |  | AA | 2.73 | 4.05 | 6.32 | 4.59 | 0.00 | 0.00 | 11.50 | 10.34 | 28.76 | 31.53 | 0.95 (0.74-1.24) |
|  |  |  |  |  |  |  |  |  |  |  |  |  | *P trend =* *0.855* |
|  |  |  |  |  |  |  |  |  |  |  |  |  |  |
| SNP 120 | rs4703500 | AA | 63.05 | 60.00 | 82.52 | 86.07 | 58.00 | 63.31 | 91.87 | 92.63 | 96.96 | 95.51 | ref |
|  |  | AG | 32.55 | 33.49 | 17.48 | 12.50 | 38.42 | 31.41 | 8.13 | 7.11 | 3.04 | 4.49 | 1.11 (0.93-1.34) |
|  |  | GG | 4.40 | 6.51 | 0.00 | 1.43 | 3.58 | 5.28 | 0.00 | 0.26 | 0.00 | 0.00 | 0.64 (0.40-1.02) |
|  |  |  |  |  |  |  |  |  |  |  |  |  | *P trend =* *0.829* |
|  |  |  |  |  |  |  |  |  |  |  |  |  |  |
| SNP 129 | rs37382 | AA | 19.59 | 19.81 | 52.43 | 59.30 | 51.44 | 55.18 | 80.61 | 75.20 | 81.06 | 78.79 | ref |
|  |  | AG | 45.61 | 47.73 | 41.75 | 33.68 | 41.87 | 37.59 | 17.88 | 22.98 | 17.68 | 19.58 | 0.98 (0.84-1.15) |
|  |  | GG | 34.80 | 32.46 | 5.83 | 7.02 | 6.70 | 7.23 | 1.52 | 1.83 | 1.26 | 1.63 | 1.00 (0.78-1.30) |
|  |  |  |  |  |  |  |  |  |  |  |  |  | *P trend =* *0.944* |
| Block 7 | |  |  |  |  |  |  |  |  |  |  |  |  |
| SNP 136 | rs43215 | CC | 38.3 | 41.33 | 71.43 | 67.82 | 48.46 | 51.32 | 82.53 | 81.98 | 82.46 | 77.85 | ref |
|  |  | CT | 46.78 | 45.13 | 27.62 | 27.34 | 42.76 | 40.53 | 15.96 | 17.23 | 16.79 | 21.00 | 0.99 (0.84-1.15) |
|  |  | TT | 14.91 | 13.54 | 0.95 | 4.84 | 8.79 | 8.15 | 1.51 | 0.78 | 0.75 | 1.14 | 1.03 (0.76-1.38) |
|  |  |  |  |  |  |  |  |  |  |  |  |  | *P trend =* *0.989* |
|  |  |  |  |  |  |  |  |  |  |  |  |  |  |
| SNP 137 | rs12655058 | GG | 98.26 | 95.31 | 97.22 | 95.17 | 98.12 | 99.05 | 90.15 | 93.75 | 90.52 | 90.89 | ref |
|  |  | AG | 1.74 | 4.69 | 2.78 | 4.83 | 1.88 | 0.95 | 9.85 | 6.25 | 8.98 | 8.88 | 1.04 (0.77-1.40) |
|  |  | AA | 0.00 | 0.00 | 0.00 | 0.00 | 0.00 | 0.00 | 0.00 | 0.00 | 0.50 | 0.23 | 1.87 (0.17-20.90) |
|  |  |  |  |  |  |  |  |  |  |  |  |  | *P trend =* *0.732* |
|  |  |  |  |  |  |  |  |  |  |  |  |  |  |
| SNP 139 | rs37387 | CC | 40.71 | 43.10 | 71.15 | 67.59 | 47.86 | 51.44 | 82.04 | 81.20 | 81.75 | 77.40 | ref |
|  |  | CT | 45.72 | 45.48 | 28.85 | 27.59 | 43.10 | 40.38 | 16.77 | 18.02 | 17.50 | 21.23 | 0.98 (0.84-1.15) |
|  |  | TT | 13.57 | 11.43 | 0.00 | 4.83 | 9.05 | 8.17 | 1.20 | 0.78 | 0.75 | 1.37 | 1.03 (0.76-1.40) |
|  |  |  |  |  |  |  |  |  |  |  |  |  | *P trend =* *0.995* |
|  |  |  |  |  |  |  |  |  |  |  |  |  |  |
| SNP 144 | rs16871473 | TT | 79.82 | 79.05 | 72.12 | 74.47 | 93.33 | 93.53 | 94.56 | 92.13 | 92.64 | 95.14 | ref |
| missense |  | CT | 18.07 | 19.52 | 26.92 | 23.40 | 6.43 | 6.47 | 5.14 | 7.87 | 7.36 | 4.63 | 0.99 (0.80-1.24) |
|  |  | CC | 2.11 | 1.43 | 0.96 | 2.13 | 0.24 | 0.00 | 0.30 | 0.00 | 0.00 | 0.23 | 1.22 (0.52-2.86) |
|  |  |  |  |  |  |  |  |  |  |  |  |  | *P trend =* *0.883* |
|  |  |  |  |  |  |  |  |  |  |  |  |  |  |
| SNP 147 | A+17112G | AA | 63.05 | 62.53 | 82.08 | 80.97 | 95.27 | 92.82 | 92.17 | 89.87 | 89.50 | 91.34 | ref |
|  |  | AG | 31.38 | 33.17 | 15.09 | 17.99 | 4.49 | 7.18 | 7.23 | 9.87 | 10.00 | 8.43 | 0.88 (0.72-1.08) |
|  |  | GG | 5.57 | 4.30 | 2.83 | 1.04 | 0.24 | 0.00 | 0.60 | 0.26 | 0.50 | 0.23 | 1.54 (0.86-2.74) |
|  |  |  |  |  |  |  |  |  |  |  |  |  | *P trend =* *0.832* |
|  |  |  |  |  |  |  |  |  |  |  |  |  |  |
| SNP 148 | rs37364 | TT | 14.41 | 16.51 | 54.72 | 47.02 | 46.12 | 47.73 | 69.18 | 65.62 | 66.83 | 64.22 | ref |
|  |  | GT | 39.41 | 43.63 | 34.91 | 43.16 | 43.53 | 43.20 | 26.59 | 30.97 | 29.65 | 30.05 | 0.90 (0.78-1.05) |
|  |  | GG | 46.18 | 39.86 | 10.38 | 9.82 | 10.35 | 9.07 | 4.23 | 3.41 | 3.52 | 5.73 | 1.07 (0.86-1.35) |
|  |  |  |  |  |  |  |  |  |  |  |  |  | *P trend =* *0.971* |
|  |  |  |  |  |  |  |  |  |  |  |  |  |  |
| SNP 149 | rs3776605 | CC | 98.55 | 97.89 | 99.07 | 98.62 | 90.95 | 89.21 | 100.00 | 100.00 | 99.75 | 100.00 | ref |
|  |  | CT | 1.45 | 2.11 | 0.93 | 1.38 | 8.81 | 10.55 | 0.00 | 0.00 | 0.25 | 0.00 | 0.81 (0.53-1.22) |
|  |  | TT | 0.00 | 0.00 | 0.00 | 0.00 | 0.24 | 0.24 | 0.00 | 0.00 | 0.00 | 0.00 | 0.90 (0.06-14.54) |
|  |  |  |  |  |  |  |  |  |  |  |  |  | *P trend =* *0.325* |
|  |  |  |  |  |  |  |  |  |  |  |  |  |  |
| SNP 151 | rs1057828 | CC | 65.47 | 64.00 | 82.41 | 81.31 | 95.06 | 93.06 | 93.11 | 90.65 | 90.77 | 91.57 | ref |
|  |  | AC | 29.73 | 31.76 | 14.81 | 17.99 | 4.71 | 6.94 | 6.89 | 9.09 | 8.73 | 8.20 | 0.86 (0.70-1.06) |
|  |  | AA | 4.80 | 4.24 | 2.78 | 0.69 | 0.24 | 0.00 | 0.00 | 0.26 | 0.50 | 0.23 | 1.30 (0.70-2.39) |
|  |  |  |  |  |  |  |  |  |  |  |  |  | *P trend =* *0.451* |
|  |  |  |  |  |  |  |  |  |  |  |  |  |  |
| SNP 153 | rs112461 | AA | 46.54 | 52.35 | 63.33 | 59.11 | 49.27 | 52.72 | 75.08 | 75.33 | 75.95 | 71.46 | ref |
|  |  | AT | 46.86 | 42.47 | 35.56 | 36.80 | 40.98 | 40.10 | 23.32 | 23.34 | 22.53 | 25.29 | 1.02 (0.87-1.18) |
|  |  | TT | 6.60 | 5.19 | 1.11 | 4.09 | 9.76 | 7.18 | 1.60 | 1.33 | 1.52 | 3.25 | 1.07 (0.77-1.50) |
|  |  |  |  |  |  |  |  |  |  |  |  |  | *P trend =* *0.700* |
| Block 8 | |  |  |  |  |  |  |  |  |  |  |  |  |
| SNP 154 | rs401694 | CC | 22.12 | 24.34 | 45.00 | 52.19 | 50.36 | 53.64 | 74.92 | 73.49 | 75.77 | 70.33 | ref |
|  |  | CT | 53.10 | 48.92 | 50.00 | 41.61 | 39.66 | 38.59 | 22.63 | 24.41 | 22.45 | 26.64 | 1.01 (0.87-1.18) |
|  |  | TT | 24.78 | 26.75 | 5.00 | 6.20 | 9.98 | 7.77 | 2.45 | 2.10 | 1.79 | 3.04 | 0.97 (0.75-1.25) |
|  |  |  |  |  |  |  |  |  |  |  |  |  | *P trend =* *0.907* |
|  |  |  |  |  |  |  |  |  |  |  |  |  |  |
| SNP 156 | rs1549623 | GG | 58.48 | 54.52 | 96.88 | 98.94 | 100.00 | 100.00 | 97.89 | 97.65 | 99.47 | 99.54 | ref |
|  |  | AG | 34.60 | 39.29 | 3.13 | 1.06 | 0.00 | 0.00 | 2.11 | 2.35 | 0.53 | 0.46 | 0.87 (0.65-1.17) |
|  |  | AA | 6.92 | 6.19 | 0.00 | 0.00 | 0.00 | 0.00 | 0.00 | 0.00 | 0.00 | 0.00 | 1.10 (0.59-2.05) |
|  |  |  |  |  |  |  |  |  |  |  |  |  | *P trend =* *0.656* |
|  |  |  |  |  |  |  |  |  |  |  |  |  |  |
| SNP 157 | rs392279 | TT | 36.95 | 39.57 | 46.08 | 51.23 | 51.07 | 52.42 | 74.55 | 75.39 | 78.59 | 75.12 | ref |
|  |  | CT | 52.49 | 47.16 | 49.02 | 41.75 | 38.66 | 39.13 | 23.35 | 23.04 | 19.65 | 23.47 | 1.03 (0.89-1.20) |
|  |  | CC | 10.56 | 13.27 | 4.90 | 7.02 | 10.26 | 8.45 | 2.10 | 1.57 | 1.76 | 1.41 | 0.99 (0.74-1.32) |
|  |  |  |  |  |  |  |  |  |  |  |  |  | *P trend =* *0.836* |
|  |  |  |  |  |  |  |  |  |  |  |  |  |  |
| SNP 161 | rs371913 | GG | 44.61 | 43.54 | 51.49 | 57.97 | 53.00 | 53.86 | 86.14 | 86.65 | 90.15 | 88.50 | ref |
|  |  | AG | 47.52 | 45.69 | 43.56 | 36.96 | 38.37 | 37.92 | 13.25 | 12.83 | 9.34 | 11.27 | 1.03 (0.87-1.21) |
|  |  | AA | 7.87 | 10.77 | 4.95 | 5.07 | 8.63 | 8.21 | 0.60 | 0.52 | 0.51 | 0.23 | 0.90 (0.65-1.25) |
|  |  |  |  |  |  |  |  |  |  |  |  |  | *P trend =* *0.840* |
| Block 9 | |  |  |  |  |  |  |  |  |  |  |  |  |
| SNP 167 | rs187490 | AA | 34.31 | 34.45 | 33.02 | 43.21 | 48.58 | 48.57 | 51.05 | 48.82 | 49.75 | 47.36 | ref |
|  |  | AG | 42.82 | 48.8 | 50.00 | 45.64 | 42.18 | 41.19 | 43.24 | 42.78 | 41.92 | 43.91 | 0.97 (0.84-1.12) |
|  |  | GG | 22.87 | 16.75 | 16.98 | 11.15 | 9.24 | 10.24 | 5.71 | 8.40 | 8.33 | 8.74 | 1.08 (0.86-1.36) |
|  |  |  |  |  |  |  |  |  |  |  |  |  | *P trend =0.753* |
|  |  |  |  |  |  |  |  |  |  |  |  |  |  |
| SNP 168 | rs37373 | TT | 40.94 | 43.20 | 32.38 | 42.71 | 47.51 | 47.62 | 52.27 | 51.06 | 49.75 | 47.25 | ref |
|  |  | CT | 42.98 | 44.87 | 50.48 | 46.18 | 43.23 | 41.90 | 42.30 | 40.74 | 41.96 | 44.27 | 1.01 (0.88-1.17) |
|  |  | CC | 16.08 | 11.93 | 17.14 | 11.11 | 9.26 | 10.48 | 5.44 | 8.20 | 8.29 | 8.49 | 1.06 (0.84-1.34) |
|  |  |  |  |  |  |  |  |  |  |  |  |  | *P trend =* *0.667* |
|  |  |  |  |  |  |  |  |  |  |  |  |  |  |
| SNP 170 | rs2270557 | GG | 76.61 | 69.03 | 95.28 | 93.4 | 77.59 | 75.66 | 93.99 | 95.04 | 95.26 | 95.22 | ref |
|  |  | AG | 20.18 | 29.08 | 4.72 | 5.90 | 21.70 | 22.67 | 6.01 | 4.96 | 4.74 | 4.78 | 0.83 (0.68-1.02) |
|  |  | AA | 3.22 | 1.89 | 0.00 | 0.69 | 0.71 | 1.67 | 0.00 | 0.00 | 0.00 | 0.00 | 0.95 (0.46-1.96) |
|  |  |  |  |  |  |  |  |  |  |  |  |  | *P trend =0.104* |
|  |  |  |  |  |  |  |  |  |  |  |  |  |  |
| SNP 171 | rs344151 | TT | 72.02 | 69.74 | 98.13 | 99.31 | 100.00 | 100.00 | 97.9 | 97.13 | 99.50 | 99.32 | ref |
|  |  | CT | 23.81 | 28.61 | 1.87 | 0.69 | 0.00 | 0.00 | 2.10 | 2.87 | 0.25 | 0.68 | 0.80 (0.59-1.09) |
|  |  | CC | 4.17 | 1.65 | 0.00 | 0.00 | 0.00 | 0.00 | 0.00 | 0.00 | 0.25 | 0.00 | 2.60 (1.05-6.49) |
|  |  |  |  |  |  |  |  |  |  |  |  |  | *P trend =* *0.989* |
|  |  |  |  |  |  |  |  |  |  |  |  |  |  |
| SNP 172 | rs37370 | TT | 92.44 | 94.13 | 59.43 | 55.02 | 28.19 | 30.55 | 51.21 | 52.09 | 79.4 | 81.51 | ref |
|  |  | CT | 7.56 | 5.63 | 35.85 | 34.60 | 50.60 | 50.60 | 39.39 | 40.31 | 18.59 | 16.44 | 1.07 (0.90-1.26) |
|  |  | CC | 0.00 | 0.23 | 4.72 | 10.38 | 21.20 | 18.85 | 9.39 | 7.59 | 2.01 | 2.05 | 1.09 (0.83-1.43) |
|  |  |  |  |  |  |  |  |  |  |  |  |  | *P trend =* *0.405* |
|  |  |  |  |  |  |  |  |  |  |  |  |  |  |

AA, African Americans; NH, Native Hawaiians; JA, Japanese Americans; LA, Latinas, WH, Whites.

^a^ ORs adjusted for age and ethnicity.

^b^ P-value for test of trend.

**Table S6. Associations between prolactin (PRL) tagSNPs and plasma PRL levels**

| SNP | SNP Name | N (%) | Genotype | LS means^a^ (95% CI) | | p^b^ |
| --- | --- | --- | --- | --- | --- | --- |
|  |  |  |  |  |  |  |
| Block 1 |  |  |  |  |  |  |
| SNP 1 | rs1205945 | 289 (90.60) | CC | 8.04 | (7.34 - 8.81) | 0.584 |
|  |  | 30 (9.40) | CT | 7.45 | (5.72 - 9.70) |  |
|  |  |  | TT |  |  |  |
|  |  |  |  |  |  |  |
| SNP 2 | rs2744098 | 201 (63.01) | GG | 8.07 | (7.25 - 8.97) | 0.868 |
|  |  | 99 (31.03) | GT | 7.77 | (6.71 – 9.00) |  |
|  |  | 19 (5.96) | TT | 8.27 | (6.00 - 11.39) |  |
|  |  |  |  |  |  |  |
| SNP 4 | rs2655419 | 212 (66.46) | GG | 8.11 | (7.31 – 9.00) | 0.521 |
|  |  | 92 (28.84) | AG | 7.84 | (6.75 - 9.11) |  |
|  |  | 15 (4.70) | AA | 7.23 | (5.04 - 10.35) |  |
|  |  |  |  |  |  |  |
| SNP 6 | rs9358533 | 191 (59.87) | CC | 7.71 | (6.90 - 8.62) | 0.418 |
|  |  | 109 (34.17) | CT | 8.46 | (7.33 - 9.78) |  |
|  |  | 19 (5.96) | TT | 8.11 | (5.79 - 11.36) |  |
|  |  |  |  |  |  |  |
| SNP 9 | rs10946546 | 156 (48.90) | CC | 7.78 | (6.83 - 8.85) | 0.902 |
|  |  | 120 (37.62) | CT | 8.44 | (7.37 - 9.66) |  |
|  |  | 43 (13.48) | TT | 7.52 | (6.02 - 9.40) |  |
|  |  |  |  |  |  |  |
| SNP 23 | rs2473122 | 220 (68.97) | GG | 7.91 | (7.14 - 8.76) | 0.491 |
|  |  | 85 (26.65) | AG | 8.01 | (6.85 - 9.37) |  |
|  |  | 14 (4.39) | AA | 9.50 | (6.51 - 13.87) |  |
|  |  |  |  |  |  |  |
| Block 2 |  |  |  |  |  |  |
| SNP 25 | rs767938 | 115 (37.95) | CC | 8.37 | (7.29 - 9.61) | 0.606 |
|  |  | 139 (45.87) | CT | 7.51 | (6.61 - 8.52) |  |
|  |  | 49 (16.17) | TT | 8.15 | (6.56 - 10.13) |  |
|  |  |  |  |  |  |  |
| SNP 28 | rs9358531 | 110 (42.80) | TT | 7.42 | (6.37 - 8.65) | 0.607 |
|  |  | 113 (43.97) | GT | 8.21 | (7.09 - 9.51) |  |
|  |  | 34 (13.23) | GG | 7.68 | (5.98 - 9.86) |  |
|  |  |  |  |  |  |  |
| SNP 30 | rs1156546 | 101 (39.30) | AA | 8.38 | (7.22 - 9.74) | 0.218 |
|  |  | 113 (43.97) | AC | 7.46 | (6.45 - 8.62) |  |
|  |  | 43 (16.73) | CC | 7.23 | (5.76 - 9.07) |  |
|  |  |  |  |  |  |  |
| SNP 32 | rs2655426 | 134 (52.14) | GG | 7.58 | (6.62 - 8.68) | 0.256 |
|  |  | 94 (36.58) | GT | 7.66 | (6.55 - 8.95) |  |
|  |  | 29 (11.28) | TT | 9.51 | (7.18 - 12.6) |  |
|  |  |  |  |  |  |  |
| SNP 33 | rs1341238 | 167 (64.98) | GG | 7.15 | (6.35 - 8.06) | 0.025 |
|  |  | 65 (25.29) | AG | 9.23 | (7.68 - 11.09) |  |
|  |  | 25 (9.73) | AA | 9.02 | (6.69 - 12.16) |  |
|  |  |  |  |  |  |  |
| SNP 34 | rs9466314 | 248 (96.50) | AA | 7.67 | (6.94 - 8.49) | 0.039 |
|  |  | 8 (3.11) | AT | 10.48 | (6.25 - 17.58) |  |
|  |  | 1 (0.39) | TT | 31.75 | (7.69 - 131.02) |  |
|  |  |  |  |  |  |  |
| SNP 36 | rs2744117 | 213 (70.30) | GG | 8.18 | (7.37 - 9.08) | 0.226 |
|  |  | 80 (26.40) | GT | 7.47 | (6.28 - 8.87) |  |
|  |  | 10 (3.30) | TT | 6.26 | (3.8 - 10.31) |  |
|  |  |  |  |  |  |  |
| SNP 38 | rs849876 | 264 (87.13) | AA | 7.81 | (7.11 - 8.58) | 0.370 |
|  |  | 38 (12.54) | AG | 8.92 | (7.07 - 11.25) |  |
|  |  | 1 (0.33) | GG | 6.14 | (1.51 - 25.02) |  |
|  |  |  |  |  |  |  |
| SNP 39 | rs3756824 | 246 (81.19) | CC | 7.56 | (6.86 - 8.34) | 0.035 |
|  |  | 56 (18.48) | CG | 9.73 | (8.00 - 11.82) |  |
|  |  | 1 (0.33) | GG | 6.15 | (1.55 - 24.49) |  |
|  |  |  |  |  |  |  |
| SNP 43 | rs12202764 | 198 (77.04) | TT | 7.65 | (6.82 - 8.58) | 0.382 |
|  |  | 50 (19.46) | AT | 7.96 | (6.30 - 10.04) |  |
|  |  | 9 (3.50) | AA | 9.89 | (6.14 - 15.94) |  |
|  |  |  |  |  |  |  |
| SNP 44 | rs2244502 | 145 (47.85) | AA | 6.96 | (6.15 - 7.88) | 0.002 |
|  |  | 126 (41.58) | AT | 8.64 | (7.60 - 9.82) |  |
|  |  | 32 (10.56) | TT | 10.30 | (7.91 - 13.41) |  |
|  |  |  |  |  |  |  |
| Block 3 |  |  |  |  |  |  |
| SNP 47 | A+4346G | 269 (96.76) | GG | 7.97 | (7.21 - 8.80) | 0.523 |
|  |  | 9 (3.24) | AG | 6.75 | (4.04 - 11.25) |  |
|  |  |  | AA |  |  |  |
|  |  |  |  |  |  |  |
| SNP 51 | rs849884 | 318 (97.85) | CC | 8.20 | (7.51 - 8.94) | 0.986 |
|  |  | 7 (2.15) | CT | 8.16 | (4.79 - 13.89) |  |
|  |  |  | TT |  |  |  |
|  |  |  |  |  |  |  |
| SNP 52 | rs7759000 | 246 (75.69) | TT | 7.95 | (7.22 - 8.75) | 0.075 |
|  |  | 70 (21.54) | CT | 8.68 | (7.20 - 10.46) |  |
|  |  | 9 (2.77) | CC | 13.23 | (8.08 - 21.65) |  |
|  |  |  |  |  |  |  |
| SNP 54 | rs849886 | 83 (25.54) | CC | 9.33 | (7.83 - 11.13) | 0.048 |
|  |  | 157 (48.31) | CT | 8.22 | (7.31 - 9.24) |  |
|  |  | 85 (26.15) | TT | 7.27 | (6.15 - 8.59) |  |
|  |  |  |  |  |  |  |
| SNP 56 | rs1205960 | 205 (63.08) | CC | 8.32 | (7.49 - 9.23) | 0.396 |
|  |  | 105 (32.31) | CT | 8.23 | (7.12 - 9.50) |  |
|  |  | 15 (4.62) | TT | 6.52 | (4.53 - 9.38) |  |
|  |  |  |  |  |  |  |
| SNP 57 | rs6239 | 228 (82.01) | CC | 7.51 | (6.74 - 8.38) | 0.056 |
|  |  | 47 (16.91) | CT | 9.91 | (8.01 - 12.26) |  |
|  |  | 3 (1.08) | TT | 7.11 | (3.12 - 16.19) |  |
|  |  |  |  |  |  |  |
| SNP 58 | rs10946545 | 228 (82.01) | GG | 8.11 | (7.25 - 9.07) | 0.511 |
|  |  | 47 (16.91) | AG | 7.41 | (6.13 - 8.96) |  |
|  |  | 3 (1.08) | AA | 7.90 | (4.99 - 12.49) |  |
|  |  |  |  |  |  |  |
| SNP 59 | rs1205961 | 196 (70.50) | GG | 8.07 | (7.09 - 9.19) | 0.905 |
|  |  | 71 (25.54) | AG | 8.41 | (7.35 - 9.62) |  |
|  |  | 11 (3.96) | AA | 8.02 | (6.29 - 10.22) |  |
|  |  |  |  |  |  |  |
| Block 4 |  |  |  |  |  |  |
| SNP 62 | rs849870 | 191 (64.09) | CC | 8.72 | (7.81 - 9.72) | 0.013 |
|  |  | 96 (32.21) | CT | 7.28 | (6.30 - 8.41) |  |
|  |  | 11 (3.69) | TT | 5.66 | (3.69 - 8.70) |  |
|  |  |  |  |  |  |  |
| SNP 65 | rs849872 | 142 (47.65) | TT | 8.96 | (7.92 - 10.14) | 0.018 |
|  |  | 123 (41.28) | CT | 7.47 | (6.59 - 8.48) |  |
|  |  | 33 (11.07) | CC | 6.93 | (5.42 - 8.85) |  |
|  |  |  |  |  |  |  |
| SNP 68 | rs2744119 | 199 (66.78) | AA | 7.83 | (7.03 - 8.72) | 0.492 |
|  |  | 86 (28.86) | AG | 8.66 | (7.35 - 10.2) |  |
|  |  | 13 (4.36) | GG | 7.84 | (5.26 - 11.68) |  |
|  |  |  |  |  |  |  |
| SNP 69 | rs2744120 | 79 (26.51) | AA | 7.74 | (6.59 - 9.08) | 0.083 |
|  |  | 135 (45.30) | AG | 7.56 | (6.70 - 8.54) |  |
|  |  | 84 (28.19) | GG | 9.43 | (8.02 - 11.08) |  |
|  |  |  |  |  |  |  |
| SNP 70 | rs12524841 | 165 (64.71) | TT | 7.73 | (6.85 - 8.73) | 0.547 |
|  |  | 77 (30.20) | GT | 8.00 | (6.76 - 9.48) |  |
|  |  | 13 (5.10) | GG | 8.76 | (5.91 - 12.96) |  |
|  |  |  |  |  |  |  |
| SNP 73 | rs2153646 | 147 (49.33) | TT | 8.57 | (7.58 - 9.68) | 0.387 |
|  |  | 122 (40.94) | AT | 7.43 | (6.52 - 8.47) |  |
|  |  | 29 (9.73) | AA | 8.47 | (6.51 - 11.01) |  |
|  |  |  |  |  |  |  |
| SNP 75 | rs6940783 | 200 (78.43) | CC | 7.61 | (6.78 - 8.53) | 0.298 |
|  |  | 49 (19.22) | CG | 8.73 | (7.07 - 10.77) |  |
|  |  | 6 (2.35) | GG | 8.40 | (4.71 - 14.99) |  |
|  |  |  |  |  |  |  |
| SNP 77 | rs2066266 | 125 (49.02) | AA | 8.46 | (7.29 - 9.83) | 0.313 |
|  |  | 90 (35.29) | AG | 7.36 | (6.32 - 8.57) |  |
|  |  | 40 (15.69) | GG | 7.61 | (6.00 - 9.64) |  |
|  |  |  |  |  |  |  |

AA, African Americans; NH, Native Hawaiians; JA, Japanese Americans; LA, Latinas, WH, Whites.

^a^ Least square means of prolactin levels and 95% confidence interval; LS means adjusted for age, ethnicity, and assay batch.

^b^ P-value for test of trend.

**Table S7. Associations between prolactin receptor (PRLR) tagSNPs and plasma PRL levels**

| SNP | SNP Name | N (%) | Genotype | LS means^a^ (95% CI) | | p^b^ |
| --- | --- | --- | --- | --- | --- | --- |
|  |  |  |  |  |  |  |
| Block 1 |  |  |  |  |  |  |
|  |  |  |  |  |  |  |
| SNP 7 | rs11741701 | 202 (61.96) | GG | 8.24 | (7.41 - 9.17) | 0.588 |
|  |  | 105 (32.21) | AG | 8.44 | (7.30 - 9.75) |  |
|  |  | 19 (5.83) | AA | 9.10 | (6.54 - 12.67) |  |
|  |  |  |  |  |  |  |
| SNP 9 | rs7726240 | 261 (80.06) | TT | 8.15 | (7.40 - 8.98) | 0.209 |
|  |  | 53 (16.26) | CT | 8.94 | (7.28 - 10.97) |  |
|  |  | 12 (3.68) | CC | 10.44 | (6.81 - 15.99) |  |
|  |  |  |  |  |  |  |
| SNP 11 | rs7734446 | 162 (58.06) | AA | 8.96 | (7.86 - 10.22) | 0.097 |
|  |  | 85 (30.47) | AG | 7.14 | (5.99 - 8.51) |  |
|  |  | 32 (11.47) | GG | 7.36 | (5.56 - 9.75) |  |
|  |  |  |  |  |  |  |
| SNP 16 | rs7728298 | 101 (36.20) | AA | 8.32 | (7.09 - 9.77) | 0.652 |
|  |  | 114 (40.86) | AG | 7.55 | (6.50 - 8.78) |  |
|  |  | 64 (22.94) | GG | 9.08 | (7.47 - 11.05) |  |
|  |  |  |  |  |  |  |
| SNP 23 | rs4703398 | 301 (92.33) | GG | 8.32 | (7.61 - 9.11) | 0.816 |
|  |  | 24 (7.36) | AG | 8.65 | (6.41 - 11.68) |  |
|  |  | 1 (0.31) | AA | 8.41 | (2.05 - 34.41) |  |
|  |  |  |  |  |  |  |
| SNP 30 | hCV11281097 | 99 (30.37) | TT | 8.59 | (7.34 - 10.05) | 0.829 |
|  |  | 139 (42.64) | GT | 8.16 | (7.19 - 9.25) |  |
|  |  | 88 (26.99) | GG | 8.40 | (7.11 - 9.92) |  |
|  |  |  |  |  |  |  |
| Block 2 |  |  |  |  |  |  |
| SNP 31 | rs10068521 | 263 (90.38) | GG | 8.19 | (7.40 - 9.06) | 0.432 |
|  |  | 26 (8.93) | CG | 8.01 | (6.02 - 10.66) |  |
|  |  | 2 (0.69) | CC | 3.99 | (1.47 - 10.83) |  |
|  |  |  |  |  |  |  |
| SNP 32 | rs7733450 | 244 (83.85) | TT | 7.97 | (7.18 - 8.84) | 0.454 |
|  |  | 42 (14.43) | AT | 8.97 | (7.12 - 11.29) |  |
|  |  | 5 (1.72) | AA | 8.02 | (4.19 - 15.35) |  |
|  |  |  |  |  |  |  |
| SNP 35 | rs6863018 | 91 (31.27) | GG | 8.38 | (7.1 - 9.88) | 0.409 |
|  |  | 130 (44.67) | AG | 8.26 | (7.21 - 9.46) |  |
|  |  | 70 (24.05) | AA | 7.51 | (6.23 - 9.05) |  |
|  |  |  |  |  |  |  |
| SNP 38 | rs931740 | 152 (44.31) | CC | 8.22 | (7.25 - 9.31) | 0.841 |
|  |  | 136 (39.65) | CT | 8.19 | (7.21 - 9.30) |  |
|  |  | 55 (16.03) | TT | 8.49 | (6.91 - 10.43) |  |
|  |  |  |  |  |  |  |
| SNP 39 | rs7731153 | 172 (59.11) | AA | 8.24 | (7.26 - 9.36) | 0.622 |
|  |  | 82 (28.18) | AG | 8.04 | (6.74 - 9.59) |  |
|  |  | 37 (12.71) | GG | 7.60 | (5.81 - 9.96) |  |
|  |  |  |  |  |  |  |
| Block 3 |  |  |  |  |  |  |
| SNP 41 | rs6451190 | 149 (53.21) | CC | 7.62 | (6.64 - 8.74) | 0.286 |
|  |  | 96 (34.29) | CG | 8.73 | (7.38 - 10.34) |  |
|  |  | 35 (12.5) | GG | 8.82 | (6.68 - 11.66) |  |
|  |  |  |  |  |  |  |
| SNP 49 | rs34024951 | 220 (78.57) | CC | 8.39 | (7.55 - 9.33) | 0.079 |
|  |  | 48 (17.14) | CT | 7.36 | (5.97 - 9.08) |  |
|  |  | 12 (4.29) | TT | 5.85 | (3.81 - 8.98) |  |
|  |  |  |  |  |  |  |
| SNP 51 | rs4703510 | 137 (41.52) | TT | 7.98 | (7.00 - 9.09) | 0.615 |
|  |  | 136 (41.21) | AT | 8.67 | (7.64 - 9.83) |  |
|  |  | 57 (17.27) | AA | 8.31 | (6.82 - 10.11) |  |
|  |  |  |  |  |  |  |
| SNP 52 | rs4235652 | 105 (37.5) | TT | 7.89 | (6.8 - 9.16) | 0.857 |
|  |  | 122 (43.57) | CT | 8.37 | (7.28 - 9.63) |  |
|  |  | 53 (18.93) | CC | 7.98 | (6.45 - 9.86) |  |
|  |  |  |  |  |  |  |
| SNP 53 | rs2047740 | 97 (29.39) | GG | 8.20 | (7.05 - 9.54) | 0.768 |
|  |  | 154 (46.67) | AG | 8.28 | (7.37 - 9.29) |  |
|  |  | 79 (23.94) | AA | 8.50 | (7.17 - 10.07) |  |
|  |  |  |  |  |  |  |
| SNP 55 | rs875701 | 88 (31.43) | AA | 8.11 | (6.89 - 9.55) | 0.853 |
|  |  | 127 (45.36) | AG | 8.21 | (7.20 - 9.38) |  |
|  |  |  | GG | 7.88 | (6.52 - 9.52) |  |
|  |  |  |  |  |  |  |
| SNP 57 | rs4703509 | 196 (59.39) | TT | 8.63 | (7.75 - 9.61) | 0.367 |
|  |  | 107 (32.42) | CT | 7.79 | (6.80 - 8.93) |  |
|  |  |  | CC | 8.21 | (6.16 - 10.96) |  |
|  |  |  |  |  |  |  |
| SNP 60 | rs1587607 | 224 (67.88) | AA | 8.37 | (7.57 - 9.26) | 0.933 |
|  |  | 90 (27.27) | AG | 8.08 | (6.98 - 9.36) |  |
|  |  |  | GG | 8.84 | (6.24 - 12.52) |  |
|  |  |  |  |  |  |  |
| SNP 63 | rs7705216 | 236 (84.29) | CC | 8.04 | (7.25 - 8.92) | 0.605 |
|  |  | 40 (14.29) | CG | 8.27 | (6.61 - 10.34) |  |
|  |  |  | GG | 9.97 | (4.99 - 19.91) |  |
|  |  |  |  |  |  |  |
| Block 4 |  |  |  |  |  |  |
| SNP 73 | rs10941235 | 135 (49.45) | CC | 8.97 | (7.78 - 10.33) | 0.042 |
|  |  | 107 (39.19) | CT | 7.09 | (6.13 - 8.20) |  |
|  |  | 31 (11.36) | TT | 7.40 | (5.67 - 9.67) |  |
|  |  |  |  |  |  |  |
| SNP 75 | rs7700286 | 220 (80.59) | TT | 8.07 | (7.20 - 9.04) | 0.434 |
|  |  | 48 (17.58) | CT | 7.46 | (6.03 - 9.23) |  |
|  |  | 5 (1.83) | CC | 6.95 | (3.64 - 13.29) |  |
|  |  |  |  |  |  |  |
| SNP 77 | rs10067710 | 189 (69.23) | AA | 8.01 | (7.06 - 9.09) | 0.766 |
|  |  | 66 (24.18) | AT | 7.84 | (6.49 - 9.48) |  |
|  |  | 18 (6.59) | TT | 7.59 | (5.20 - 11.09) |  |
|  |  |  |  |  |  |  |
| SNP 78 | rs34212252 | 248 (76.31) | GG | 8.00 | (7.23 - 8.86) | 0.361 |
|  |  | 66 (20.31) | CG | 8.08 | (6.77 - 9.64) |  |
|  |  | 11 (3.38) | CC | 10.86 | (7.09 - 16.63) |  |
|  |  |  |  |  |  |  |
| SNP 79 | rs6895193 | 101 (37.00) | GG | 7.41 | (6.35 - 8.65) |  |
|  |  | 116 (42.49) | AG | 7.97 | (6.85 - 9.28) | 0.137 |
|  |  | 56 (20.51) | AA | 9.02 | (7.33 - 11.1) |  |
|  |  |  |  |  |  |  |
| SNP 84 | rs12518881 | 253 (77.85) | GG | 8.43 | (7.64 - 9.31) | 0.065 |
|  |  | 70 (21.54) | AG | 7.31 | (6.12 - 8.73) |  |
|  |  | 2 (0.62) | AA | 3.91 | (1.46 - 10.49) |  |
|  |  |  |  |  |  |  |
| SNP 86 | rs1039427 | 172 (52.92) | CC | 8.66 | (7.67 - 9.76) | 0.259 |
|  |  | 119 (36.62) | CT | 7.42 | (6.49 - 8.49) |  |
|  |  | 34 (10.46) | TT | 8.22 | (6.44 - 10.49) |  |
|  |  |  |  |  |  |  |
| Block 5 |  |  |  |  |  |  |
| SNP 98 | rs7732013 | 232 (78.38) | CC | 7.87 | (7.06 - 8.77) | 0.497 |
|  |  | 55 (18.58) | CT | 9.11 | (7.25 - 11.45) |  |
|  |  | 9 (3.04) | TT | 7.64 | (4.64 - 12.58) |  |
|  |  |  |  |  |  |  |
| SNP 99 | rs2047741 | 242 (69.14) | CC | 8.00 | (7.24 - 8.84) | 0.478 |
|  |  | 87 (24.86) | CG | 8.65 | (7.32 - 10.22) |  |
|  |  | 21 (6.00) | GG | 8.57 | (6.16 - 11.9) |  |
|  |  |  |  |  |  |  |
| SNP 102 | rs1587605 | 292 (83.43) | TT | 8.14 | (7.44 - 8.91) | 0.606 |
|  |  | 55 (15.71) | CT | 8.22 | (6.74 - 10.03) |  |
|  |  | 3 (0.86) | CC | 12.56 | (5.68 - 27.79) |  |
|  |  |  |  |  |  |  |
| SNP 106 | rs3797212 | 306 (87.43) | CC | 8.30 | (7.6 - 9.07) | 0.244 |
|  |  | 43 (12.29) | CT | 7.80 | (6.21 - 9.8) |  |
|  |  | 1 (0.29) | TT | 1.69 | (0.43 - 6.64) |  |
|  |  |  |  |  |  |  |
| SNP 111 | rs2962086 | 220 (74.32) | AA | 7.77 | (6.97 - 8.67) | 0.090 |
|  |  | 68 (22.97) | AG | 8.39 | (6.94 - 10.13) |  |
|  |  | 8 (2.70) | GG | 13.99 | (8.42 - 23.25) |  |
|  |  |  |  |  |  |  |
| SNP 112 | rs930068 | 277 (93.58) | TT | 8.15 | (7.37 - 9.02) | 0.377 |
|  |  | 18 (6.08) | GT | 6.92 | (4.83 - 9.90) |  |
|  |  | 1 (0.34) | GG | 6.75 | (1.64 - 27.76) |  |
|  |  |  |  |  |  |  |
| Block 6 |  |  |  |  |  |  |
| SNP 114 | rs35322400 | 254 (74.27) | TT | 8.22 | (7.46 - 9.06) | 0.823 |
|  |  | 80 (23.39) | CT | 8.36 | (7.01 - 9.95) |  |
|  |  | 8 (2.34) | CC | 8.67 | (5.19 - 14.47) |  |
|  |  |  |  |  |  |  |
| SNP 117 | rs1966571 | 213 (62.28) | TT | 8.18 | (7.34 - 9.11) | 0.766 |
|  |  | 104 (30.41) | CT | 8.37 | (7.18 - 9.76) |  |
|  |  | 25 (7.31) | CC | 8.53 | (6.26 - 11.63) |  |
|  |  |  |  |  |  |  |
| SNP 119 | rs7734558 | 190 (55.56) | GG | 8.42 | (7.51 - 9.44) | 0.263 |
|  |  | 123 (35.96) | AG | 8.67 | (7.56 - 9.95) |  |
|  |  | 29 (8.48) | AA | 6.34 | (4.84 - 8.31) |  |
|  |  |  |  |  |  |  |
| SNP 120 | rs4703500 | 278 (81.29) | AA | 8.27 | (7.54 - 9.08) | 0.729 |
|  |  | 57 (16.67) | AG | 8.31 | (6.79 - 10.18) |  |
|  |  | 7 (2.05) | GG | 6.83 | (3.98 - 11.72) |  |
|  |  |  |  |  |  |  |
| SNP 129 | rs37382 | 203 (59.36) | AA | 8.52 | (7.63 - 9.52) | 0.561 |
|  |  | 110 (32.16) | AG | 7.73 | (6.67 - 8.96) |  |
|  |  | 29 (8.48) | GG | 8.41 | (6.28 - 11.25) |  |
|  |  |  |  |  |  |  |
| Block 7 |  |  |  |  |  |  |
| SNP 136 | rs43215 | 171 (61.73) | CC | 7.87 | (6.99 - 8.87) | 0.449 |
|  |  | 96 (34.66) | CT | 8.05 | (6.90 - 9.40) |  |
|  |  | 10 (3.61) | TT | 10.39 | (6.45 - 16.73) |  |
|  |  |  |  |  |  |  |
| SNP 137 | rs12655058 | 255 (92.06) | GG | 8.00 | (7.23 - 8.86) | 0.901 |
|  |  | 22 (7.94) | AG | 7.84 | (5.76 - 10.68) |  |
|  |  |  | AA |  |  |  |
|  |  |  |  |  |  |  |
| SNP 139 | rs37387 | 173 (62.45) | CC | 7.84 | (6.98 - 8.81) | 0.383 |
|  |  | 94 (33.94) | CT | 8.12 | (6.96 - 9.48) |  |
|  |  | 10 (3.61) | TT | 10.41 | (6.47 - 16.76) |  |
|  |  |  |  |  |  |  |
| SNP 144 | rs16871473 | 289 (87.58) | TT | 8.09 | (7.39 - 8.85) | 0.746 |
| missense |  | 36 (10.91) | CT | 8.48 | (6.56 - 10.97) |  |
|  |  | 5 (1.52) | CC | 6.01 | (3.22 - 11.21) |  |
|  |  |  |  |  |  |  |
| SNP 147 | A+17112G | 232 (83.75) | AA | 7.74 | (6.96 - 8.6) | 0.133 |
|  |  | 40 (14.44) | AG | 9.22 | (7.2 - 11.8) |  |
|  |  | 5 (1.81) | GG | 10.66 | (5.58 - 20.36) |  |
|  |  |  |  |  |  |  |
| SNP 148 | rs37364 | 136 (49.1) | TT | 7.34 | (6.44 - 8.35) | 0.037 |
|  |  | 106 (38.27) | GT | 8.49 | (7.32 - 9.86) |  |
|  |  | 35 (12.64) | GG | 9.72 | (7.5 - 12.6) |  |
|  |  |  |  |  |  |  |
| SNP 149 | rs3776605 | 264 (95.31) | CC | 8.09 | (7.33 - 8.91) | 0.100 |
|  |  | 13 (4.69) | CT | 5.72 | (3.80 - 8.61) |  |
|  |  |  | TT |  |  |  |
|  |  |  |  |  |  |  |
| SNP 151 | rs1057828 | 235 (84.84) | CC | 7.72 | (6.95 - 8.57) | 0.100 |
|  |  | 37 (13.36) | AC | 9.45 | (7.33 - 12.19) |  |
|  |  | 5 (1.81) | AA | 10.72 | (5.62 - 20.46) |  |
|  |  |  |  |  |  |  |
| SNP 153 | rs112461 | 173 (62.45) | AA | 7.78 | (6.94 - 8.72) | 0.360 |
|  |  | 92 (33.21) | AT | 8.37 | (7.13 - 9.84) |  |
|  |  | 12 (4.33) | TT | 8.84 | (5.82 - 13.43) |  |
|  |  |  |  |  |  |  |
| Block 8 |  |  |  |  |  |  |
| SNP 154 | rs401694 | 193 (55.94) | CC | 8.18 | (7.33 - 9.12) | 0.849 |
|  |  | 120 (34.78) | CT | 8.23 | (7.18 - 9.44) |  |
|  |  | 32 (9.28) | TT | 8.43 | (6.51 - 10.93) |  |
|  |  |  |  |  |  |  |
| SNP 156 | rs1549623 | 311 (90.14) | GG | 8.07 | (7.38 - 8.83) | 0.252 |
|  |  | 29 (8.41) | AG | 9.48 | (6.89 - 13.05) |  |
|  |  | 5 (1.45) | AA | 10.67 | (5.62 - 20.26) |  |
|  |  |  |  |  |  |  |
| SNP 157 | rs392279 | 204 (59.13) | TT | 8.21 | (7.39 - 9.13) | 0.684 |
|  |  | 119 (34.49) | CT | 8.00 | (6.98 - 9.17) |  |
|  |  | 22 (6.38) | CC | 9.52 | (7.03 - 12.89) |  |
|  |  |  |  |  |  |  |
| SNP 161 | rs371913 | 224 (64.93) | GG | 8.36 | (7.56 - 9.25) | 0.748 |
|  |  | 102 (29.57) | AG | 7.80 | (6.71 - 9.06) |  |
|  |  | 19 (5.51) | AA | 8.74 | (6.30 - 12.13) |  |
|  |  |  |  |  |  |  |
| Block 9 |  |  |  |  |  |  |
| SNP 167 | rs187490 | 131 (43.81) | AA | 8.09 | (7.10 - 9.21) | 0.724 |
|  |  | 135 (45.15) | AG | 8.03 | (7.03 - 9.17) |  |
|  |  | 33 (11.04) | GG | 8.75 | (6.7 - 11.43) |  |
|  |  |  |  |  |  |  |
| SNP 168 | rs37373 | 137 (45.82) | TT | 8.08 | (7.11 - 9.17) | 0.771 |
|  |  | 132 (44.15) | CT | 8.09 | (7.06 - 9.26) |  |
|  |  | 30 (10.03) | CC | 8.56 | (6.5 - 11.28) |  |
|  |  |  |  |  |  |  |
| SNP 170 | rs2270557 | 260 (86.96) | GG | 8.13 | (7.36 - 8.98) | 0.943 |
|  |  | 38 (12.71) | AG | 8.00 | (6.24 - 10.26) |  |
|  |  | 1 (0.33) | AA | 9.00 | (2.14 - 37.8) |  |
|  |  |  |  |  |  |  |
| SNP 171 | rs344151 | 277 (92.64) | TT | 8.13 | (7.36 - 8.97) | 0.885 |
|  |  | 21 (7.02) | CT | 7.26 | (5.10 - 10.32) |  |
|  |  | 1 (0.33) | CC | 28.39 | (6.76 - 119.28) |  |
|  |  |  |  |  |  |  |
| SNP 172 | rs37370 | 177 (59.20) | TT | 8.54 | (7.52 - 9.69) | 0.365 |
|  |  | 92 (30.77) | CT | 7.48 | (6.34 - 8.82) |  |
|  |  | 30 (10.03) | CC | 7.86 | (5.97 - 10.36) |  |

AA, African Americans; NH, Native Hawaiians; JA, Japanese Americans; LA, Latinas, WH, Whites.

^a^ Least square means of prolactin levels and 95% confidence interval; LS means adjusted for age, ethnicity, and assay batch.

^b^ P-value for test of trend.

**Table S8. Common haplotypes frequencies in linkage disequilibrium (LD) blocks of prolactin (PRL)**

| Haplotypes^a^ |  | Haplotype frequencies (%) in the multiethnic panel^b^ | | | | |
| --- | --- | --- | --- | --- | --- | --- |
|  |  | AA | NH | JA | LA | WH |
|  | | |  |  |  |  |
| Block 1:  SNPs 1-24; tag SNPs 1, 2, 4, 6, 9, 23 | | |  |  |  |  |
|  |  |  |  |  |  |  |
| 1A | CGGCTG | 16.4 | 39.9 | 33.3 | 28.5 | 37.9 |
| 1B | CGGCCG | 28.2 | 13.2 | 8.8 | 37.8 | 40.9 |
| 1C | CGGTCG | 27.9 | 23.1 | 28.6 | 11.6 | 9.8 |
| 1D | CTACCA | 3.4 | 9.1 | 7.3 | 7.3 | 1.4 |
| 1E | TTACCA | 12.1 | 3.6 |  | 1.0 | 6.4 |
| 1F | CGACCA | 1.3 | 6.1 | 14.7 | 10.3 | 1.4 |
| 1G | CGGCCA | 6.1 |  |  |  |  |
| Total^c^ |  | 95.4 | 94.9 | 92.7 | 96.5 | 97.9 |
| Minimum *R_h_^2^*^d^ |  | 72.5 | 86.0 | 71.8 | 87.2 | 84.5 |
|  |  |  |  |  |  |  |
| “Block” 2:  SNPs 25-45; tag SNPs 25, 28, 30, 32, 33, 34, 36, 38, 39, 43, 44 | | | |  |  |  |
|  |  |  |  |  |  |  |
| 2A | CGAGGAGAGTA | 16.7 | 38.1 | 32.1 | 27.1 | 38.5 |
| 2B | CTCGGAGAGTT | 6.0 |  |  | 2.1 |  |
| 2C | CTCGGAGACTT |  | 6.4 | 1.5 |  |  |
| 2D | CTCGGATAGTA | 2.1 | 14.6 | 25.3 | 8.9 | 5.7 |
| 2E | TTATAAGAGAT | 1.7 | 5.1 |  | 11.4 | 14.9 |
| 2F | TTATAAGACTT |  | 4.3 | 17.8 | 11.4 | 2.0 |
| 2G | TTATAAGGGTT | 10.8 | 3.6 |  | 2.1 | 5.8 |
| 2H | TTCGGAGAGTA | 17.8 | 10.2 |  | 20.3 | 9.8 |
| 2I | TTATAAGAGTA | 6.6 | 3.0 | 2.9 | 2.4 | 5.9 |
| 2J | TTCGGAGACTT | 1.4 |  | 5.0 |  |  |
| 2K | CTCGGAGAGAT | 5.3 |  |  |  |  |
| Total^c^ |  | 68.4 | 85.2 | 84.6 | 85.8 | 82.5 |
| Minimum *R_h_^2^*^d^ |  | 55.8 | 84.5 | 99.4 | 91.0 | 91.7 |

| Block 3:  SNPs 46-59; tag SNPs 47, 51, 52, 54, 56, 57, 58, 59 | | |  |  |  |  |
| --- | --- | --- | --- | --- | --- | --- |
|  |  |  |  |  |  |  |
| 3A | GCTTCCGG | 18.3 | 29.4 | 42.1 | 34.8 | 37.5 |
| 3B | GCTCCCGA | 14.4 |  |  | 3.1 |  |
| 3C | GCTCTCGG | 6.7 | 4.3 | 8.4 | 3.1 | 11.1 |
| 3D | GCTCTCGA | 25.4 | 16.7 | 1.4 | 12.2 | 10.6 |
| 3E | GCTTCCAG | 2.9 | 18.8 | 22.9 | 6.4 | 7.1 |
| 3F | GCCCCCGG | 7.0 | 1.6 |  | 4.8 | 4.3 |
| 3G | GCCCCCGA | 10.0 | 4.8 |  | 9.1 | 16.7 |
| 3H | GTTCTCGA | 1.7 | 2.8 |  | 1.4 | 5.1 |
| 3I | GCTCCTGG | 1.4 | 10.9 | 21.3 | 16.6 |  |
| 3J | GCTTCCGA | 5.2 | 3.1 |  | 5.2 | 5.3 |
| 3K | ACTCCCGA | 5.7 |  |  |  |  |
| Total^c^ |  | 98.7 | 92.5 | 96.2 | 96.8 | 97.7 |
| Minimum *R_h_^2^*^d^ |  | 77.0 | 92.8 | 91.0 | 89.9 | 83.7 |
|  |  |  |  |  |  |  |
| Block 4:  SNPs 61-77; tag SNPs 62, 65, 68, 69, 70, 73, 75, 77 | | | |  |  |  |
|  |  |  |  |  |  |  |
| 4A | CTAGTTCA | 28.4 | 26.2 | 36.1 | 33.5 | 39.0 |
| 4B | CTAATTGG | 7.9 | 8.4 |  | 12.1 | 27.1 |
| 4C | CTAAGACG | 19.9 | 2.9 | 2.1 | 6.4 | 5.7 |
| 4D | CTGGTTCA | 3.7 | 16.6 | 17.4 | 17.2 | 11.8 |
| 4E | CCAAGACA | 2.1 | 10.9 | 30.7 | 15.7 |  |
| 4F | TCAATTCG | 12.6 | 19.2 |  | 1.4 | 1.4 |
| 4G | TCAATACG | 3.0 | 7.6 | 4.1 | 10.7 | 10.7 |
| 4H | CTAATACA | 2.8 | 3.6 | 5.7 |  |  |
| 4I | CTAAGACA | 5.6 |  |  |  |  |
| Total^c^ |  | 85.9 | 95.3 | 96.3 | 97.0 | 95.6 |
| Minimum *R_h_^2^*^d^ |  | 90.2 | 71.1 | 96.9 | 72.8 | 86.1 |

^a^ Haplotypes observed with ≥5% frequency in at least one ethnic group.

^b^ Frequencies estimated using the tagging SNPs; AA, African Americans; NH, Native Hawaiians; JA, Japanese Americans; LA, Latinas; WH, Whites.

^c^ The percentage of all chromosomes accounted for by the haplotypes.

^d^ The *R_h_^2^* that is given is the minimum *R_h_^2^* of the common haplotype in each ethnic group.

**Table S9. Common haplotypes frequencies in linkage disequilibrium (LD) blocks of prolactin receptor (PRLR)**

| Haplotypes^a^ |  | | Haplotype frequencies (%) in the multiethnic panel^b^ | | | | |
| --- | --- | --- | --- | --- | --- | --- | --- |
|  |  | | AA | NH | JA | LA | WH |
|  | | | |  |  |  |  |
| Block 1:  SNPs 6-30; tag SNPs 6, 7, 9, 11, 12, 16, 23, 24, 29, 30 | | | |  |  |  |  |
|  |  | |  |  |  |  |  |
| 1A1 | AGTATGGATG | | 9.2 | 17.2 | 5.0 | 16.9 | 34.6 |
| 1A2 | GGTATGGATG | |  | 4.5 |  | 6.4 | 5.9 |
| 1A3 | AGTACGGATG | | 2.2 |  |  | 6.6 |  |
| 1B | GGTACAGATT | | 8.2 | 5.8 | 5.4 | 12.1 | 8.0 |
| 1C | GGTACGGATT | | 14.5 |  |  | 1.1 |  |
| 1D | GGTGCAGATT | | 4.3 | 42.8 | 45.7 | 14.7 | 3.8 |
| 1E | GGCACGGGTT | | 20.6 | 3.7 | 2.2 | 1.8 | 3.7 |
| 1F | GATACAGATG | | 21.5 | 20.3 | 23.5 | 21.4 | 35.0 |
| 1G | GGTACGAATT | | 1.4 |  | 6.1 | 7.5 | 2.9 |
| 1H | GGTGCAGATG | | 7.1 |  |  | 7.5 |  |
| Total^c^ |  | | 89.0 | 94.2 | 87.9 | 96.0 | 94.0 |
| Minimum *R_h_^2^*^d^ |  | | 91.2 | 99.8 | 98.2 | 88.6 | 100.0 |
|  |  | |  |  |  |  |  |
| Block 1: without tag SNPs 6, 12, 24, 29 | | | |  |  |  |  |
| 1A = 1A1+1A2+1A3 | | GTAGGG | 13.7 | 21.7 | 5.8 | 31.7 | 41.4 |
| 1B | | GTAAGT | 8.7 | 5.8 | 5.3 | 11.0 | 8.0 |
| 1C | | GTAGGT | 20.8 |  |  | 1.2 |  |
| 1D | | GTGAGT | 5.0 | 42.8 | 46.4 | 14.5 | 4.8 |
| 1E | | GCAGGT | 22.1 | 7.3 | 7.6 | 1.7 | 7.1 |
| 1F | | ATAAGG | 22.8 | 21.0 | 23.4 | 21.4 | 35.0 |
| 1G | | GTAGAT | 4.4 |  | 7.7 | 7.6 | 2.9 |
| 1H | | GTGAGG |  |  |  | 7.6 |  |
| Total^c^ | |  | 97.5 | 98.6 | 96.3 | 96.7 | 99.3 |
| Minimum *R_h_^2^*^d^ | | | 46.3 | 75.0 | 61.2 | 14.9 | 8.9 |
|  |  | |  |  |  |  |  |
| Block 2:  SNPs 31-39; tag SNPs 31, 32, 35, 38, 39 | | | | |  |  |  |
|  |  | |  |  |  |  |  |
| 2A | GTACA | | 43.6 | 39.9 | 29.8 | 61.3 | 69.2 |
| 2B | GTGCA | | 16.1 |  |  |  |  |
| 2C | GTGTA | | 21.0 | 8.7 | 3.7 | 14.4 | 15.0 |
| 2D | GTGTG | | 3.9 | 42.0 | 47.8 | 12.0 | 4.2 |
| 2E | GAGCA | | 10.9 | 7.3 | 8.6 | 2.1 | 7.1 |
| 2F | CTGCA | | 3.5 |  | 3.0 | 8.6 | 2.9 |
| Total^c^ |  | | 98.9 | 97.8 | 92.8 | 98.5 | 98.4 |
| Minimum *R_h_^2^*^d^ |  | | 94.3 | 99.9 | 99.5 | 98.9 | 99.2 |

| Block 3:  SNPs 41-66; tag SNPs 41, 49, 51, 52, 53, 55, 57, 60, 63 | | |  |  |  |  |
| --- | --- | --- | --- | --- | --- | --- |
|  |  |  |  |  |  |  |
| 3A | CCTTAGTAC | 35.5 | 31.2 | 24.7 | 55.6 | 65.0 |
| 3B | CCTTAGTAG | 1.5 | 7.3 |  |  |  |
| 3C | CCTTGACAC | 1.1 | 5.1 | 2.4 | 5.0 | 3.6 |
| 3D | CCTTGACGC | 5.8 |  |  |  |  |
| 3E | CCTTGACGG | 1.3 |  |  | 5.0 | 10.4 |
| 3F | CTACGACGC | 23.9 | 5.8 | 4.3 | 8.6 | 8.6 |
| 3G | GCACGATAC | 3.6 | 41.3 | 60.7 | 14.3 | 7.9 |
| 3H | CCTCGACGG |  | 5.6 |  |  | 3.2 |
| 3I | CCTTAGTGC | 3.6 |  | 5.3 |  |  |
| Total^c^ |  | 76.3 | 96.2 | 97.4 | 88.5 | 98.6 |
| Minimum *R_h_^2^*^d^ |  | 81.2 | 93.4 | 99.4 | 87.6 | 90.2 |
|  |  | 96.7^e^ |  |  |  |  |

| Block 4:  SNPs 73-88; tag SNPs 73, 75, 77, 78, 79, 84, 86 | | | |  |  |  |
| --- | --- | --- | --- | --- | --- | --- |
|  |  |  |  |  |  |  |
| 4A | CTTGGGC | 10.7 | 13.8 |  | 26.4 | 32.8 |
| 4B | CTAGGGC | 10.7 | 3.6 | 11.4 | 2.2 | 1.5 |
| 4C | CTAGAGC | 33.8 | 42.8 | 75.0 | 41.4 | 34.3 |
| 4D | TTAGGGT |  | 15.2 |  | 13.5 | 6.1 |
| 4E | TTAGGAT | 6.1 | 10.1 |  | 3.4 | 14.2 |
| 4F | TTACGGT | 16.0 | 2.2 |  | 2.4 | 2.6 |
| 4G | TCACGGT | 7.3 | 8.0 | 12.1 | 8.3 | 7.9 |
| Total^c^ |  | 84.6 | 95.6 | 98.6 | 97.6 | 99.3 |
| Minimum *R_h_^2^*^d^ |  | 93.8 | 99.3 | 100.0 | 96.4 | 96.9 |
|  |  |  |  |  |  |  |
| Block 5:  SNPs 95-113; tag SNPs 98, 99, 102, 106, 111, 112 | | |  |  |  |  |
|  |  |  |  |  |  |  |
| 5A | CCTCAT | 47.2 | 72.5 | 77.1 | 89.3 |  |
| 5B | CCTTAT |  | 6.5 |  |  |  |
| 5C | CGCCGT | 9.9 | 9.4 | 17.9 | 2.9 |  |
| 5D | TGTCGT | 17.3 |  |  |  |  |
| 5E | TGTTAT | 7.8 | 9.4 | 5.0 | 2.9 | 1.4 |
| Total^c^ |  | 82.1 | 97.8 | 100.0 | 95.0 | 1.4 |
| Minimum *R_h_^2^*^d^ |  | 85.6 | 91.6 | 100.0 | 92.4 | 92.0 |
|  |  | 87.3^e^ |  |  |  |  |
| Block 6:  SNPs 114-135; tag SNPs 114, 117, 119, 120, 129 | | | |  |  |  |
|  |  |  |  |  |  |  |
| 6A | TTAAA | 15.1 | 21.3 |  | 29.5 | 53.8 |
| 6B | TTGAA | 22.2 | 51.2 | 70.6 | 59.0 | 34.7 |
| 6C | TCGAG | 15.4 | 16.7 | 5.7 | 1.8 | 2.4 |
| 6D | TCGGG | 8.9 |  |  |  |  |
| 6E | CCGAG | 9.9 |  |  | 2.1 | 5.3 |
| 6F | CCGGG | 14.4 | 10.1 | 18.2 | 2.1 |  |
| Total^c^ |  | 86.0 | 99.2 | 94.5 | 94.4 | 96.2 |
| Minimum *R_h_^2^*^d^ |  | 52.4 | 97.1 | 90.0 | 98.1 | 96.9 |
|  |  | 78.7^e^ |  |  |  |  |
| Block 7:  SNPs 136-153; tag SNPs 136, 137, 139, 144, 147, 148, 149, 151, 153 | | | |  |  |  |
|  |  |  |  |  |  |  |
| 7A | CGCTATCCT | 31.3 | 58.7 | 57.7 | 79.1 | 68.2 |
| 7B | CGCTAGCCA | 5.4 | 7.3 | 2.2 | 5.1 | 11.0 |
| 7C | CGCCATCCT |  | 8.7 |  |  |  |
| 7D | TGTTAGCCT | 11.0 |  |  |  |  |
| 7E | TGTTAGCCA | 18.2 | 16.7 | 26.3 | 6.4 | 7.6 |
| 7F | CACTATCCT |  | 1.5 |  | 4.3 | 5.8 |
| 7G | CGCCGGCCT | 6.7 | 3.3 |  |  |  |
| 7H | CGCTAGCCT | 5.3 |  |  |  |  |
| Total^c^ |  | 77.9 | 96.0 | 86.2 | 94.9 | 92.6 |
| Minimum *R_h_^2^*^d^ |  | 56.9 | 100.0 | 96.8 | 95.3 | 95.6 |
|  |  | 87.5^e^ |  |  |  |  |

| Block 8:  SNPs 154-161; tag SNPs 154, 156, 157, 161 | | | |  |  |  |
| --- | --- | --- | --- | --- | --- | --- |
|  |  |  |  |  |  |  |
| 8A | CGTG | 34.1 | 71.7 | 67.1 | 85.7 | 79.1 |
| 8B | CATG | 14.3 |  |  | 1.5 |  |
| 8C | TGCG |  | 4.4 |  | 3.6 | 8.5 |
| 8D | TGCA | 31.2 | 22.4 | 28.6 | 5.7 | 6.4 |
| 8E | TATG | 17.0 |  |  |  |  |
| Total^c^ |  | 96.5 | 98.5 | 95.7 | 96.4 | 94.0 |
| Minimum *R_h_^2^*^d^ |  | 85.0 | 95.9 | 99.9 | 99.6 | 99.2 |
|  |  |  |  |  |  |  |
| Block 9:  SNPs 167-173; tag SNPs 167, 168, 170, 171, 172 | | |  |  |  |  |
|  |  |  |  |  |  |  |
| 9A | ATGTT | 29.8 | 35.9 | 16.6 | 45.1 | 61.0 |
| 9B | ATGTC |  | 27.2 | 39.8 | 26.9 | 2.5 |
| 9C | ATGCT | 16.0 |  |  | 1.5 |  |
| 9D | ATATT | 12.9 | 3.6 | 12.9 | 3.0 |  |
| 9E | GCGTT | 32.0 | 32.6 | 28.2 | 22.9 | 33.9 |
| Total^c^ |  | 90.6 | 99.3 | 97.5 | 99.4 | 97.4 |
| Minimum *R_h_^2^*^d^ |  | 83.9 | 100.0 | 97.8 | 95.7 | 100.0 |

^a^ Haplotypes observed with ≥5% frequency in at least one ethnic group.

^b^ Frequencies estimated using the tagging SNPs; AA, African Americans; NH, Native Hawaiians; JA, Japanese Americans; LA, Latinas; WH, Whites.

^c^ The percentage of all chromosomes accounted for by the haplotypes.

^d^ The *R_h_^2^* that is given is the minimum *R_h_^2^* of the common haplotype in each ethnic group.

^e^ The *R_h_^2^* that is given is the minimum *R_h_^2^* of the common haplotype for African Americans for African American-specific block sizes: block 3 (49-58), block 5 (102-113), block 6 (114-124), and block 7 (147-153).

**Table S10. Associations between prolactin (PRL) haplotypes and breast cancer risk**

|  | | Haplotype^a^ | Racial-Ethnic Groups | | | | | |  |  |
| --- | --- | --- | --- | --- | --- | --- | --- | --- | --- | --- |
|  | |  | % Cases/ % Controls | | | | | |  |  |
|  | |  | AA | | NH | JA | LA | WH | OR^b^ (95% CI)^c^ | P |
| No. cases/controls | | | 345/426 | | 109/290 | 425/420 | 335/386 | 401/440 |  |  |
|  |  | |  | |  |  |  |  |  |  |
| Block 1 | | |  | |  |  |  |  |  |  |
| 1A | CGGCTG | | 12.10/ 11.97 | | 41.86/ 39.92 | 32.08/ 34.16 | 31.12/ 32.89 | 39.17/ 39.96 | 1.00 |  |
| 1B | CGGCCG | | 29.10/ 30.05 | | 17.18/ 14.31 | 9.32/ 9.41 | 29.81/ 27.83 | 37.46/ 37.45 | 1.06 (0.93-1.21) | *0.401* |
| 1C | CGGTCG | | 27.76/ 29.69 | | 20.06/ 24.69 | 30.07/ 32.53 | 16.25/ 14.73 | 10.73/ 8.94 | 1.01 (0.88-1.16) | *0.935* |
| 1D | CTACCA | | 6.25/ 5.45 | | 12.72/ 15.60 | 21.8/ 18.16 | 17.33/ 20.04 | 4.37/ 3.89 | 1.07 (0.91-1.26) | *0.434* |
| 1E | TTACCA | | 8.40/ 10.98 | | 1.28/ 1.90 | 0.10/ 0.32 | 3.77/ 2.13 | 6.48/ 6.98 | 0.94 (0.73-1.20) | *0.606* |
|  |  | |  | |  |  |  |  |  |  |
| “Block” 2 | | |  | |  |  |  |  |  |  |
| 2A | CGAGGAGAGTA | | 11.03/ 11.11 | | 39.33/ 37.16 | 30.4/ 32.66 | 26.85/ 28.68 | 37.55/ 37.73 | 1.00 |  |
| 2B | CTCGGAGAGTT | | 6.88/ 7.19 | | 0.06/ 0.33 | 0.25/ 0.23 | 1.30/ 1.63 | 0.64/ 0.50 | 0.95 (0.65-1.38) | *0.775* |
| 2C | CTCGGAGACTT | | 0.00/ 0.00 | | 5.51/ 5.24 | 1.27/ 1.62 | 0.42/ 0.10 | 0.16/ 0.12 | 1.00 (0.58-1.73) | *0.991* |
| 2D | CTCGGATAGTA | | 2.85/ 2.40 | | 13.29/ 16.22 | 26.55/ 28.23 | 8.49/ 7.25 | 6.67/ 4.79 | 1.05 (0.88-1.25) | *0.572* |
| 2E | TTATAAGAGAT | | 3.12/ 2.63 | | 0.67/ 1.11 | 0.00/ 0.12 | 5.77/ 5.06 | 5.96/ 5.62 | 1.09 (0.84-1.41) | *0.521* |
| 2F | TTATAAGACTT | | 0.86/ 0.51 | | 4.08/ 4.87 | 18.22/ 14.44 | 10.2/ 11.29 | 2.87/ 2.83 | 1.16 (0.94-1.42) | *0.165* |
| 2G | TTATAAGGGTT | | 7.05/ 8.42 | | 0.94/ 2.21 | 0.12/ 0.24 | 2.87/ 2.14 | 4.85/ 6.21 | 0.86 (0.65-1.13) | *0.269* |
| 2H | TTCGGAGAGTA | | 18.27/ 17.28 | | 11.73/ 11.96 | 1.87/ 1.21 | 9.99/ 12.36 | 8.68/ 9.09 | 1.00 (0.83-1.20) | *0.966* |
|  |  | |  | |  |  |  |  |  |  |
| Block 3 | | |  | |  |  |  |  |  |  |
| 3A | GCTTCCGG | | 18.17/ 18.67 | | 31.57/ 30.31 | 42.22/ 43.93 | 32.57/ 35.03 | 36.74/ 36.28 | 1.00 |  |
| 3B | GCTCCCGA | | 13.81/ 14.15 | | 1.15/ 0.64 | 0.00/ 0.00 | 2.74/ 3.69 | 1.31/ 1.24 | 0.95 (0.72-1.26) | *0.734* |
| 3C | GCTCTCGG | | 4.80/ 5.55 | | 3.60/ 5.04 | 6.16/ 7.09 | 6.40/ 6.22 | 11.15/ 10.85 | 0.94 (0.76-1.16) | *0.539* |
| 3D | GCTCTCGA | | 26.95/ 27.06 | | 18.23/ 18.31 | 2.74/ 2.24 | 10.92/ 11.87 | 9.62/ 10.59 | 1.02 (0.86-1.20) | *0.867* |
| 3E | GCTTCCAG | | 2.50/ 2.93 | | 17.17/ 20.17 | 22.51/ 25.88 | 9.03/ 7.68 | 7.75/ 6.10 | 0.99 (0.84-1.17) | *0.886* |
| 3F | GCCCCCGG | | 6.50/ 5.31 | | 2.55/ 0.82 | 0.00/ 0.00 | 4.52/ 4.38 | 6.03/ 4.80 | 1.30 (0.97-1.75) | *0.077* |
| 3G | GCCCCCGA | | 18.38/ 15.00 | | 5.25/ 4.50 | 0.12/ 0.24 | 12.46/ 11.24 | 14.9/ 17.4 | 1.07 (0.89-1.28) | *0.489* |
| 3H | GTTCTCGA | | 0.91/ 1.41 | | 0.11/ 1.22 | 0.01/ 0.24 | 2.86/ 1.46 | 3.88/ 5.08 | 0.87 (0.60-1.26) | *0.471* |
| 3I | GCTCCTGG | | 0.00/ 0.00 | | 12.66/ 13.78 | 23.62/ 18.38 | 0.00/ 0.00 | 0.00/ 0.00 | 1.27 (1.02-1.59) | *0.036* |
|  |  | |  | |  |  |  |  |  |  |
| Global test^d^ χ2 = 10.69, df = 10, p = 0.382 | | | | | |  |  |  |  |  |
|  | |  | |  |  |  |  |  |  |  |
| Block 4 | | | |  |  |  |  |  |  |  |
| 4A | CTAGTTCA | | | 29.55/ 29.41 | 26.3/ 21.99 | 25.19/ 30.55 | 31.14/ 31.65 | 40.71/ 38.7 | 1.00 |  |
| 4B | CTAATTGG | | | 12.93/ 11.29 | 5.99/ 7.42 | 0.12/ 0.36 | 16.35/ 15.15 | 23.86/ 23.87 | 1.05 (0.90-1.24) | *0.533* |
| 4C | CTAAGACG | | | 14.84/ 16.96 | 1.46/ 2.61 | 1.09/ 1.86 | 5.19/ 4.06 | 4.85/ 5.86 | 0.89 (0.72-1.10) | *0.278* |
| 4D | CTGGTTCA | | | 2.95/ 3.11 | 24.38/ 18.48 | 28.66/ 27.15 | 17.80/ 14.09 | 11.09/ 11.11 | 1.12 (0.97-1.31) | *0.133* |
| 4E | CCAAGACA | | | 1.46/ 0.85 | 10.22/ 14.08 | 32.22/ 28.16 | 13.60/ 18.01 | 2.99/ 2.40 | 1.04 (0.88-1.22) | *0.660* |
| 4F | TCAATTCG | | | 18.74/ 17.05 | 11.92/ 15.08 | 0.00/ 0.08 | 3.28/ 3.62 | 3.07/ 3.70 | 0.98 (0.80-1.22) | *0.880* |
| 4G | TCAATACG | | | 6.19/ 5.97 | 8.58/ 8.86 | 4.17/ 3.37 | 8.60/ 9.82 | 10.65/ 9.80 | 1.05 (0.86-1.27) | *0.635* |
|  |  | | |  |  |  |  |  |  |  |

^a^ Haplotypes with frequencies ≥ 5% in the multiethnic panel are shown.

^b^ Odds Ratio and 95% confidence interval; ORs adjusted for age and ethnicity.

^c^ Reference group is the most common haplotype.

^d^ The most common haplotype was the reference group. All rare haplotypes (<5%) were combined as one group. Degrees of freedom (df) = number of haplotypes (common and rare haplotype combined)-1.

**Table S11. Associations between prolactin receptor (PRLR) haplotypes and breast cancer risk**

|  | | Haplotype^a^ | | Racial-Ethnic Groups | | | | |  |  |
| --- | --- | --- | --- | --- | --- | --- | --- | --- | --- | --- |
|  | |  | | % Cases/ % Controls | | | | |  |  |
|  | |  | | AA | NH | JA | LA | WH | OR^b^ (95% CI)^c^ | P |
| No. cases/controls | | | | 345/426 | 109/290 | 425/420 | 335/386 | 401/440 |  |  |
|  |  | | |  |  |  |  |  |  |  |
| Block 1^d^ | | | | |  |  |  |  |  |  |
| 1A | GTAGGG | | 13.91/ 14.83 | | 23.53/ 25.8 | 5.27/ 7.68 | 28.75/ 27.6 | 38.48/ 39.91 | 1.00 |  |
| 1B | GTAAGT | | 7.18/ 8.48 | | 5.06/ 4.90 | 2.86/ 4.09 | 14.43/ 12.53 | 12.29/ 8.10 | 1.16 (0.95-1.41) | *0.151* |
| 1C | GTAGGT | | 28.55/ 24.39 | | 0.79/ 0.40 | 0.00/ 0.00 | 1.98/ 1.77 | 0.99/ 1.46 | 1.25 (0.98-1.59) | *0.073* |
| 1D | GTGAGT | | 2.84/ 2.64 | | 50.47/ 42 | 47.1/ 48.57 | 14.63/ 14.88 | 8.57/ 6.57 | 1.10 (0.94-1.29) | *0.225* |
| 1E | GCAGGT | | 17.27/ 22.16 | | 5.17/ 5.96 | 11.33/ 10.19 | 2.84/ 2.54 | 4.36/ 4.89 | 0.96 (0.79-1.17) | *0.693* |
| 1F | ATAAGG | | 21.63/ 19.88 | | 10.47/ 17.79 | 24.87/ 20.32 | 24.56/ 27.92 | 31.9/ 34.84 | 1.03 (0.90-1.19) | *0.637* |
|  |  | |  | |  |  |  |  |  |  |
| Block 2 | | |  | |  |  |  |  |  |  |
| 2A | GTACA | | 42.57/ 40.54 | | 31.41/ 43.02 | 31.08/ 28.78 | 58.53/ 60.6 | 67.44/ 69.91 | 1.00 |  |
| 2B | GTGCA | | 26.82/ 27.29 | | 0.02/ 0.55 | 0.71/ 0.32 | 3.61/ 3.73 | 1.53/ 2.00 | 0.98 (0.79-1.21) | *0.837* |
| 2C | GTGTA | | 16.71/ 22.44 | | 9.87/ 6.82 | 3.32/ 3.53 | 17.81/ 15.59 | 15.98/ 12.59 | 1.04 (0.89-1.21) | *0.631* |
| 2D | GTGTG | | 2.08/ 2.63 | | 49.86/ 40.41 | 46.42/ 49.01 | 12.86/ 12.11 | 8.04/ 5.94 | 1.07 (0.93-1.24) | *0.330* |
| 2E | GAGCA | | 0.00/ 0.00 | | 4.56/ 6.34 | 11.55/ 10.27 | 0.00/ 0.00 | 4.46/ 5.35 | 1.03 (0.81-1.32) | *0.791* |
| 2F | CTGCA | | 9.24/ 4.67 | | 1.84/ 1.21 | 0.96/ 1.36 | 5.09/ 6.08 | 2.00/ 3.21 | 1.16 (0.89-1.50) | *0.267* |
|  |  | |  | |  |  |  |  |  |  |
| Block 3 | | |  | |  |  |  |  |  |  |
| 3A | CCTTAGTAC | | 41.27/ 35.32 | | 22.78/ 31.8 | 28.62/ 26.55 | 55.45/ 57.19 | 65.29/ 66.52 | 1.00 |  |
| 3B | CCTTAGTAG | | 2.08/ 1.04 | | 6.36/ 8.93 | 0.00/ 0.00 | 0.30/ 0.15 | 0.13/ 0.02 | 1.02 (0.64-1.63) | *0.926* |
| 3C | CCTTGACAC | | 2.84/ 1.63 | | 9.49/ 6.63 | 2.38/ 1.92 | 5.25/ 6.08 | 1.88/ 3.16 | 1.05 (0.80-1.38) | *0.723* |
| 3D | CCTTGACGC | | 6.93/ 6.99 | | 1.37/ 0.37 | 0.69/ 0.58 | 0.32/ 0.65 | 0.26/ 0.24 | 0.97 (0.67-1.41) | *0.864* |
| 3E | CCTTGACGG | | 1.97/ 4.07 | | 7.46/ 4.82 | 1.06/ 1.43 | 10.29/ 7.52 | 15.20/ 11.48 | 1.19 (0.98-1.46) | *0.084* |
| 3F | CTACGACGC | | 16.22/ 20.07 | | 2.42/ 4.86 | 6.92/ 6.90 | 6.84/ 7.66 | 7.60/ 7.83 | 0.85 (0.71-1.01) | *0.070* |
| 3G | GCACGATAC | | 4.35/ 4.22 | | 43.82/ 38.44 | 54.3/ 55.43 | 18.5/ 18.37 | 8.59/ 8.08 | 1.01 (0.88-1.16) | *0.888* |
|  |  | |  | |  |  |  |  |  |  |
| Block 4 | | |  | |  |  |  |  |  |  |
| 4A | CTTGGGC | | 11.24/ 10.63 | | 11.97/ 17.03 | 0.47/ 1.19 | 29.49/ 25.79 | 35.96/ 35.59 | 1.00 |  |
| 4B | CTAGGGC | | 16.92/ 13.09 | | 3.01/ 4.51 | 13.29/ 10.65 | 3.71/ 3.69 | 1.42/ 1.39 | 1.17 (0.94-1.45) | *0.166* |
| 4C | CTAGAGC | | 30.68/ 29.67 | | 42.01/ 41.07 | 67.87/ 68.98 | 40.31/ 44.52 | 33.31/ 35.3 | 0.95 (0.83-1.10) | *0.488* |
| 4D | TTAGGGT | | 2.61/ 2.42 | | 9.17/ 8.08 | 2.23/ 1.42 | 5.51/ 6.55 | 3.71/ 4.48 | 0.96 (0.74-1.26) | *0.780* |
| 4E | TTAGGAT | | 3.47/ 4.88 | | 13.91/ 14.34 | 0.82/ 1.31 | 10.33/ 7.68 | 15.18/ 12.03 | 1.10 (0.89-1.35) | *0.391* |
| 4F | TTACGGT | | 18.33/ 21.64 | | 0.10/ 1.20 | 0.00/ 0.00 | 8.70/ 9.94 | 2.05/ 2.58 | 0.82 (0.65-1.03) | *0.086* |
| 4G | TCACGGT | | 0.00/ 0.00 | | 7.77/ 8.49 | 14.11/ 15.35 | 0.00/ 0.00 | 7.47/ 7.20 | 0.92 (0.73-1.16) | *0.487* |
|  |  | |  | |  |  |  |  |  |  |
| Block 5 | | |  | |  |  |  |  |  |  |
| 5A | CCTCAT | | 47.44/ 50.06 | | 73.38/ 76.36 | 77.29/ 78.17 | 89.39/ 87.65 | 89.13/ 90.69 | 1.00 |  |
| 5B | CCTTAT | | 0.00/ 0.00 | | 7.62/ 7.41 | 0.00/ 0.00 | 0.00/ 0.00 | 0.00/ 0.12 | 0.99 (0.55-1.78) | *0.964* |
| 5C | CGCCGT | | 9.39/ 9.81 | | 8.74/ 7.05 | 16.82/ 16.57 | 2.18/ 2.32 | 0.48/ 0.19 | 1.04 (0.86-1.26) | *0.672* |
| 5D | TGTCGT | | 17.94/ 15.77 | | 0.93/ 1.03 | 0.00/ 0.00 | 1.04/ 0.91 | 0.12/ 0.06 | 1.21 (0.93-1.58) | *0.147* |
| 5E | TGTTAT | | 5.38/ 6.44 | | 7.38/ 6.03 | 4.22/ 4.05 | 2.29/ 3.88 | 3.23/ 2.41 | 0.97 (0.77-1.23) | *0.815* |
|  |  | |  | |  |  |  |  |  |  |
| Block 6 | | |  | |  |  |  |  |  |  |
| 6A | TTAAA | | 14.33/ 16.15 | | 20.03/ 18.36 | 3.5/ 1.96 | 30.72/ 30.83 | 52.66/ 50.87 | 1.00 |  |
| 6B | TTGAA | | 27.08/ 26.63 | | 53.12/ 56.99 | 65.39/ 68.56 | 57.39/ 55.64 | 36.18/ 37.25 | 0.95 (0.84-1.08) | *0.461* |
| 6C | TCGAG | | 21.49/ 15.96 | | 14.91/ 13.89 | 5.04/ 4.35 | 2.69/ 4.62 | 3.60/ 2.58 | 1.16 (0.94-1.42) | *0.163* |
| 6D | TCGGG | | 8.33/ 8.11 | | 0.02/ 0.18 | 0.83/ 0.99 | 0.18/ 0.53 | 0.00/ 0.24 | 0.89 (0.61-1.29) | *0.528* |
| 6E | CCGAG | | 10.44/ 10.80 | | 2.04/ 0.77 | 0.00/ 0.02 | 2.41/ 3.30 | 3.77/ 3.48 | 0.94 (0.72-1.23) | *0.644* |
| 6F | CCGGG | | 12.35/ 14.98 | | 9.27/ 7.68 | 19.21/ 17.63 | 3.88/ 3.37 | 1.52/ 2.02 | 0.97 (0.80-1.18) | *0.763* |
|  |  | |  | |  |  |  |  |  |  |
| Block 7 | | |  | |  |  |  |  |  |  |
| 7A | CGCTATCCT | | 31.59/ 34.87 | | 62.66/ 62.1 | 59.49/ 59.95 | 79.09/ 78.32 | 78.18/ 76.22 | 1.00 |  |
| 7B | CGCTAGCCA | | 10.00/ 7.66 | | 4.12/ 4.22 | 1.46/ 1.69 | 7.38/ 7.28 | 7.79/ 8.6 | 1.04 (0.85-1.28) | *0.692* |
| 7C | CGCCATCCT | | 0.00/ 0.00 | | 6.55/ 4.90 | 0.74/ 0.24 | 0.30/ 0.28 | 0.13/ 0.24 | 1.45 (0.82-2.56) | *0.205* |
| 7D | TGTTAGCCT | | 11.38/ 11.39 | | 0.47/ 0.01 | 0.36/ 0.50 | 0.32/ 0.55 | 0.00/ 0.22 | 0.95 (0.69-1.32) | *0.768* |
| 7E | TGTTAGCCA | | 19.65/ 17.86 | | 13.16/ 17.53 | 27.23/ 24.74 | 5.59/ 5.69 | 4.58/ 6.84 | 1.00 (0.86-1.15) | *0.945* |
|  |  | |  | |  |  |  |  |  |  |
| Block 8 | | |  | |  |  |  |  |  |  |
| 8A | CGTG | | 36.54/ 35.76 | | 69.56/ 71.27 | 69.17/ 71.26 | 83.83/ 84.64 | 86.28/ 82.83 | 1.00 |  |
| 8B | CATG | | 11.75/ 12.37 | | 0.68/ 0.31 | 0.00/ 0.00 | 0.76/ 0.66 | 0.26/ 0.02 | 0.97 (0.70-1.34) | *0.853* |
| 8C | TGCG | | 4.70/ 2.70 | | 2.08/ 3.27 | 1.68/ 0.50 | 5.43/ 5.75 | 6.06/ 6.59 | 1.11 (0.87-1.42) | *0.405* |
| 8D | TGCA | | 31.53/ 33.35 | | 25.88/ 23.81 | 26.71/ 25.78 | 6.91/ 6.82 | 5.16/ 5.60 | 0.98 (0.86-1.12) | *0.792* |
| 8E | TATG | | 12.47/ 13.26 | | 0.45/ 0.00 | 0.00/ 0.00 | 0.30/ 0.51 | 0.00/ 0.00 | 0.93 (0.68-1.26) | *0.622* |
|  |  | |  | |  |  |  |  |  |  |
| Block 9 | | |  | |  |  |  |  |  |  |
| 9A | ATGTT | | 22.72/ 23.73 | | 31.4/ 34.26 | 12.44/ 11.97 | 39.37/ 38.60 | 56.46/ 56.20 | 1.00 |  |
| 9B | ATGTC | | 3.79/ 3.05 | | 22.76/ 27.34 | 45.2/ 43.45 | 29.12/ 27.80 | 11.32/ 10.28 | 1.04 (0.90-1.19) | *0.608* |
| 9C | ATGCT | | 15.65/ 15.57 | | 0.93/ 0.34 | 0.00/ 0.00 | 0.81/ 1.29 | 0.37/ 0.34 | 0.97 (0.73-1.28) | *0.818* |
| 9D | ATATT | | 13.31/ 16.47 | | 2.37/ 3.65 | 11.42/ 12.90 | 2.86/ 2.48 | 2.37/ 2.39 | 0.86 (0.70-1.05) | *0.142* |
| 9E | GCGTT | | 37.46/ 34.44 | | 42.46/ 34 | 29.89/ 29.94 | 26.52/ 28.7 | 29.47/ 30.44 | 1.02 (0.90-1.15) | *0.771* |
|  |  | |  | |  |  |  |  |  |  |

^a^ Haplotypes with frequencies ≥ 5% in the multiethnic panel are shown.

^b^ Odds Ratio and 95% confidence interval; ORs adjusted for age and ethnicity.

^c^ Reference group is the most common haplotype.

^d^ Haplotypes for block 1 were based on tagSNPs 7, 9, 11, 16, 23, 30 (rs11741701, rs7726240, rs7734446, rs7728298, rs4703398, hCV11281097), without tagSNPs 6, 12, 24, 29 (rs9986182, rs9292582, rs6451192, rs7701473).

**Table S12. Associations between prolactin (PRL) haplotypes and breast cancer risk by race/ethnicity**

|  |  |  | OR^a^ (95% CI) |  |  |  |
| --- | --- | --- | --- | --- | --- | --- |
|  | AA | NH | JA | LA | WH | P^b^ |
| Block 1 |  |  |  |  |  |  |
| 1A | 1.00 | 1.00 | 1.00 | 1.00 | 1.00 | *0.764* |
| 1B | 0.95 (0.67-1.37) | 1.22 (0.74-1.99) | 1.06 (0.73-1.53) | 1.15 (0.88-1.49) | 1.03 (0.81-1.30) | *0.275* |
| 1C | 0.93 (0.65-1.33) | 0.74 (0.48-1.16) | 1.00 (0.78-1.27) | 1.17 (0.84-1.62) | 1.23 (0.86-1.75) | *0.795* |
| 1D | 1.17 (0.69-1.96) | 0.74 (0.44-1.24) | 1.28 (0.97-1.69) | 0.92 (0.68-1.25) | 1.14 (0.71-1.84) | *0.132* |
| 1E | 0.75 (0.48-1.18) | 0.61 (0.16-2.30) | 0.20 (0.01-3.69) | 1.94 (1.00-3.74) | 0.93 (0.62-1.40) | *0.209* |
|  |  |  |  |  |  |  |
| “Block” 2 |  |  |  |  |  |  |
| 2A | 1.00 | 1.00 | 1.00 | 1.00 | 1.00 | *0.232* |
| 2B | 0.98 (0.58-1.63) |  | 1.18 (0.10-13.46) | 0.78 (0.30-2.03) | 1.10 (0.26-4.70) | *0.601* |
| 2C |  | 0.94 (0.44-1.99) | 0.84 (0.34-2.07) |  | 1.22 (0.05-28.22) | *0.218* |
| 2D | 1.21 (0.57-2.59) | 0.76 (0.45-1.26) | 1.01 (0.79-1.31) | 1.22 (0.79-1.90) | 1.44 (0.91-2.28) | *0.932* |
| 2E | 1.20 (0.61-2.36) | 0.55 (0.08-3.95) |  | 1.15 (0.73-1.79) | 1.06 (0.73-1.55) | *0.252* |
| 2F | 1.94 (0.50-7.44) | 0.84 (0.36-1.98) | 1.44 (1.06-1.96) | 0.92 (0.61-1.37) | 1.01 (0.56-1.81) | *0.470* |
| 2G | 0.81 (0.49-1.33) | 0.38 (0.08-1.82) | 0.54 (0.05-6.05) | 1.46 (0.72-2.96) | 0.76 (0.48-1.19) | *0.984* |
| 2H | 1.09 (0.73-1.62) | 0.94 (0.55-1.61) | 1.84 (0.75-4.50) | 0.85 (0.58-1.24) | 0.92 (0.63-1.34) | *0.428* |
|  |  |  |  |  |  |  |
| Block 3 |  |  |  |  |  |  |
| 3A | 1.00 | 1.00 | 1.00 | 1.00 | 1.00 | *0.225* |
| 3B | 0.99 (0.67-1.45) | 1.67 (0.23-11.92) |  | 0.69 (0.33-1.41) | 0.86 (0.31-2.37) | *0.875* |
| 3C | 0.83 (0.47-1.47) | 0.75 (0.29-1.92) | 0.87 (0.57-1.31) | 1.17 (0.72-1.93) | 0.98 (0.68-1.41) | *0.830* |
| 3D | 1.04 (0.75-1.43) | 1.08 (0.68-1.73) | 1.35 (0.72-2.53) | 0.96 (0.66-1.40) | 0.89 (0.62-1.28) | *0.264* |
| 3E | 0.85 (0.43-1.68) | 0.85 (0.52-1.40) | 0.92 (0.72-1.17) | 1.27 (0.84-1.92) | 1.28 (0.84-1.94) | *0.330* |
| 3F | 1.26 (0.73-2.19) | 3.94 (0.94-16.56) |  | 1.11 (0.63-1.96) | 1.35 (0.83-2.20) | *0.203* |
| 3G | 1.26 (0.89-1.80) | 1.13 (0.51-2.52) | 0.53 (0.05-6.03) | 1.24 (0.86-1.79) | 0.84 (0.62-1.14) | *0.112* |
| 3H | 0.69 (0.24-1.93) |  |  | 2.26 (1.04-4.89) | 0.71 (0.42-1.18) | *0.093* |
| 3I |  | 0.91 (0.52-1.58) | 1.39 (1.07-1.81) |  |  | *0.193* |
|  |  |  |  |  |  |  |
| Block 4 |  |  |  |  |  |  |
| 4A | 1.00 | 1.00 | 1.00 | 1.00 | 1.00 | *0.726* |
| 4B | 1.14 (0.81-1.59) | 0.65 (0.32-1.33) | 0.39 (0.04-3.67) | 1.09 (0.80-1.50) | 0.96 (0.75-1.24) | *0.368* |
| 4C | 0.86 (0.63-1.18) | 0.47 (0.13-1.69) | 0.67 (0.29-1.58) | 1.28 (0.77-2.14) | 0.81 (0.52-1.27) | *0.439* |
| 4D | 0.91 (0.49-1.70) | 1.05 (0.66-1.66) | 1.28 (0.98-1.66) | 1.29 (0.94-1.78) | 0.90 (0.65-1.25) | *0.017* |
| 4E | 1.64 (0.63-4.28) | 0.63 (0.35-1.12) | 1.40 (1.09-1.80) | 0.76 (0.55-1.05) | 1.21 (0.66-2.21) | *0.767* |
| 4F | 1.09 (0.81-1.49) | 0.67 (0.39-1.15) |  | 0.92 (0.50-1.69) | 0.81 (0.46-1.44) | *0.779* |
| 4G | 1.03 (0.65-1.64) | 0.87 (0.46-1.65) | 1.54 (0.92-2.59) | 0.90 (0.61-1.32) | 1.02 (0.73-1.43) | *0.278* |
|  |  |  |  |  |  |  |

AA, African Americans; NH, Native Hawaiians; JA, Japanese Americans; LA, Latinas, WH, Whites.

^a^ Effect per haplotype copy and adjusted for age.

^b^ P for heterogeneity of effects across racial-ethnic groups.

**Table S13. Associations between prolactin receptor (PRLR) haplotypes and breast cancer risk by race/ethnicity**

|  |  |  | OR^a^ (95% CI) |  |  |  |
| --- | --- | --- | --- | --- | --- | --- |
|  | AA | NH | JA | LA | WH | P^b^ |
| Block 1 | (without tagSNP 6,12,24,29) | |  |  |  |  |
| 1A | 1.00 | 1.00 | 1.00 | 1.00 | 1.00 | *0.039* |
| 1B | 0.83 (0.52-1.34) | 1.02 (0.47-2.21) | 1.05 (0.56-1.99) | 1.12 (0.79-1.59) | 1.62 (1.14-2.32) | *0.499* |
| 1C | 1.34 (0.94-1.90) | 2.45 (0.26-23.42) |  | 1.08 (0.45-2.59) | 0.65 (0.26-1.66) | *0.147* |
| 1D | 1.15 (0.58-2.29) | 1.32 (0.89-1.97) | 1.41 (0.94-2.11) | 0.94 (0.67-1.31) | 1.36 (0.92-2.00) | *0.274* |
| 1E | 0.80 (0.56-1.17) | 0.96 (0.44-2.12) | 1.60 (0.99-2.58) | 1.04 (0.54-2.02) | 0.94 (0.58-1.54) | *0.001* |
| 1F | 1.18 (0.82-1.68) | 0.62 (0.35-1.1) | 1.78 (1.16-2.72) | 0.83 (0.62-1.11) | 0.96 (0.77-1.20) | *0.512* |
|  |  |  |  |  |  |  |
| Block 2 |  |  |  |  |  |  |
| 2A | 1.00 | 1.00 | 1.00 | 1.00 | 1.00 | *0.645* |
| 2B | 0.92 (0.71-1.19) |  | 2.61 (0.48-14.12) | 1.01 (0.57-1.78) | 0.74 (0.35-1.56) | *0.002* |
| 2C | 0.68 (0.51-0.91) | 1.91 (1.07-3.41) |  | 1.20 (0.89-1.61) | 1.36 (1.01-1.81) | *0.042* |
| 2D | 0.75 (0.35-1.61) | 1.67 (1.17-2.38) | 0.87 (0.70-1.09) | 1.11 (0.80-1.54) | 1.40 (0.95-2.07) | *0.344* |
| 2E |  | 1.07 (0.49-2.32) | 1.01 (0.73-1.42) |  | 0.90 (0.56-1.43) | *0.004* |
| 2F | 1.94 (1.26-2.99) | 1.96 (0.54-7.15) | 0.61 (0.23-1.58) | 0.88 (0.55-1.39) | 0.62 (0.32-1.18) | *0.714* |
|  |  |  |  |  |  |  |
| Block 3 |  |  |  |  |  |  |
| 3A | 1.00 | 1.00 | 1.00 | 1.00 | 1.00 | *0.156* |
| 3B | 2.05 (0.81-5.18) | 0.92 (0.47-1.83) | 1.13 (0.58-2.21) | 2.06 (0.20-21.63) |  | *0.077* |
| 3C | 1.71 (0.76-3.87) | 2.04 (1.05-3.97) | 1.08 (0.30-3.92) | 0.92 (0.58-1.46) | 0.59 (0.30-1.14) | *0.513* |
| 3D | 0.86 (0.56-1.32) | 5.18 (0.74-36.13) | 0.70 (0.29-1.69) | 0.50 (0.09-2.70) | 1.06 (0.14-7.89) | *0.010* |
| 3E | 0.40 (0.20-0.77) | 1.94 (1.01-3.76) | 0.93 (0.62-1.42) | 1.42 (0.97-2.10) | 1.38 (1.03-1.87) | *0.665* |
| 3F | 0.68 (0.50-0.92) | 0.63 (0.23-1.73) | 0.78 (0.51-1.18) | 0.94 (0.63-1.41) | 1.00 (0.69-1.45) | *0.507* |
| 3G | 0.90 (0.54-1.50) | 1.52 (1.02-2.26) | 0.91 (0.73-1.14) | 1.06 (0.80-1.41) | 1.08 (0.75-1.56) | *0.070* |
|  |  |  |  |  |  |  |
| Block 4 |  |  |  |  |  |  |
| 4A | 1.00 | 1.00 | 1.00 | 1.00 | 1.00 | *0.580* |
| 4B | 1.19 (0.79-1.79) | 0.97 (0.37-2.57) | 3.00 (0.91-9.94) | 0.88 (0.49-1.59) | 0.92 (0.39-2.15) | *0.554* |
| 4C | 0.97 (0.67-1.4) | 1.47 (0.89-2.44) | 2.36 (0.73-7.60) | 0.80 (0.62-1.03) | 0.95 (0.75-1.20) | *0.707* |
| 4D | 1.04 (0.51-2.12) | 1.73 (0.82-3.66) | 3.61 (0.93-13.94) | 0.73 (0.46-1.19) | 0.80 (0.47-1.37) | *0.088* |
| 4E | 0.64 (0.34-1.20) | 1.31 (0.73-2.36) | 1.51 (0.34-6.80) | 1.18 (0.79-1.77) | 1.30 (0.94-1.80) | *0.841* |
| 4F | 0.78 (0.53-1.15) |  |  | 0.77 (0.51-1.15) | 0.83 (0.42-1.67) | *0.885* |
| 4G |  | 1.24 (0.62-2.51) | 2.24 (0.67-7.50) |  | 0.98 (0.67-1.44) | *0.013* |
|  |  |  |  |  |  |  |
| Block 5 |  |  |  |  |  |  |
| 5A | 1.00 | 1.00 | 1.00 | 1.00 | 1.00 | *0.776* |
| 5B |  | 1.06 (0.58-1.92) |  |  |  | *0.833* |
| 5C | 1.00 (0.69-1.44) | 1.23 (0.70-2.16) | 1.03 (0.80-1.34) | 0.91 (0.45-1.84) | 2.70 (0.38-19.02) | *0.989* |
| 5D | 1.19 (0.90-1.58) | 1.02 (0.20-5.26) |  | 1.15 (0.39-3.40) | 1.31 (0.03-55.51) | *0.342* |
| 5E | 0.88 (0.56-1.36) | 1.30 (0.69-2.45) | 1.04 (0.64-1.71) | 0.58 (0.31-1.08) | 1.25 (0.71-2.21) | *0.804* |
|  |  |  |  |  |  |  |
| Block 6 |  |  |  |  |  |  |
| 6A | 1.00 | 1.00 | 1.00 | 1.00 | 1.00 | *0.401* |
| 6B | 1.17 (0.84-1.64) | 0.87 (0.58-1.32) | 0.49 (0.26-0.94) | 1.02 (0.81-1.29) | 0.94 (0.76-1.16) | *0.072* |
| 6C | 1.54 (1.08-2.20) | 1.00 (0.58-1.73) |  | 0.58 (0.31-1.05) | 1.36 (0.76-2.44) | *0.657* |
| 6D | 1.16 (0.73-1.83) |  | 0.41 (0.12-1.41) | 0.32 (0.04-2.68) |  | *0.414* |
| 6E | 1.07 (0.71-1.63) | 2.83 (0.64-12.43) |  | 0.74 (0.38-1.43) | 1.02 (0.60-1.73) | *0.457* |
| 6F | 0.94 (0.64-1.38) | 1.08 (0.58-2.04) | 0.56 (0.29-1.11) | 1.11 (0.62-1.97) | 0.81 (0.37-1.76) | *0.205* |
|  |  |  |  |  |  |  |
| Block 7 |  |  |  |  |  |  |
| 7A | 1.00 | 1.00 | 1.00 | 1.00 | 1.00 | *0.834* |
| 7B | 1.48 (0.99-2.20) | 0.74 (0.31-1.78) | 0.83 (0.38-1.84) | 1.02 (0.68-1.54) | 0.89 (0.62-1.28) | *0.155* |
| 7C |  | 1.29 (0.64-2.61) | 3.42 (0.66-17.78) | 1.03 (0.14-7.41) | 0.55 (0.05-6.28) | *0.696* |
| 7D | 1.08 (0.75-1.55) |  | 0.71 (0.16-3.20) | 0.53 (0.10-2.93) |  | *0.062* |
| 7E | 1.21 (0.89-1.64) | 0.66 (0.40-1.08) | 1.11 (0.89-1.39) | 0.97 (0.61-1.54) | 0.62 (0.40-0.96) | *0.754* |
|  |  |  |  |  |  |  |
| Block 8 |  |  |  |  |  |  |
| 8A | 1.00 | 1.00 | 1.00 | 1.00 | 1.00 | *0.713* |
| 8B | 0.94 (0.66-1.34) | 2.79 (0.20-38.33) |  | 1.15 (0.33-4.03) |  | *0.042* |
| 8C | 1.75 (0.98-3.10) | 0.60 (0.20-1.82) | 3.44 (1.14-10.34) | 0.95 (0.60-1.50) | 0.89 (0.59-1.33) | *0.785* |
| 8D | 0.92 (0.72-1.18) | 1.11 (0.76-1.63) | 1.07 (0.86-1.32) | 1.03 (0.68-1.57) | 0.88 (0.57-1.35) | *0.838* |
| 8E | 0.92 (0.66-1.28) |  |  | 0.59 (0.11-3.35) |  | *0.018* |
|  |  |  |  |  |  |  |
| Block 9 |  |  |  |  |  |  |
| 9A | 1.00 | 1.00 | 1.00 | 1.00 | 1.00 | *0.612* |
| 9B | 1.30 (0.73-2.32) | 0.96 (0.63-1.45) | 1.00 (0.73-1.37) | 1.04 (0.80-1.34) | 1.07 (0.78-1.45) | *0.627* |
| 9C | 1.03 (0.74-1.44) | 3.45 (0.45-26.29) |  | 0.39 (0.11-1.38) | 0.99 (0.24-4.07) | *0.780* |
| 9D | 0.83 (0.59-1.17) | 0.76 (0.28-2.03) | 0.84 (0.57-1.26) | 1.09 (0.56-2.15) | 0.95 (0.50-1.84) | *0.964* |
| 9E | 1.12 (0.86-1.46) | 1.44 (0.98-2.11) | 0.95 (0.68-1.32) | 0.89 (0.68-1.17) | 0.95 (0.76-1.19) | *0.418* |
|  |  |  |  |  |  |  |

AA, African Americans; NH, Native Hawaiians; JA, Japanese Americans; LA, Latinas, WH, Whites.

^a^ Effect per haplotype copy and adjusted for age.

^b^ P for heterogeneity of effects across racial-ethnic groups.

# Table S14. Associations between prolactin (PRL) haplotypes and prolactin levels

|  |  | LS means^b^ (95% CI) | | |  |
| --- | --- | --- | --- | --- | --- |
|  | Haplotype^a^ | None | One copy | Two copies | P^c^ |
| Block 1 |  |  |  |  |  |
| 1A | CGGCTG | 8.7 (7.8 - 9.8) | 7.3 (6.3 - 8.5) | 8.6 (5.8 - 12.6) | *0.514* |
| 1B | CGGCCG | 8.0 (7.0 - 9.2) | 8.7 (7.6 - 10.0) | 7.5 (5.8 - 9.6) | *0.611* |
| 1C | CGGTCG | 8.2 (7.4 - 9.2) | 8.2 (7.0 - 9.6) | 8.2 (5.8 - 11.6) | *0.611* |
| 1D | CTACCA | 8.0 (7.2 - 8.8) | 9.0 (7.5 - 10.9) | 10.7 (6.2 - 18.3) | *0.476* |
| 1E | TTACCA | 8.2 (7.5 – 9.0) | 8.7 (6.5 - 11.8) |  | *0.602* |
|  |  |  |  |  |  |
| “Block” 2 |  |  |  |  |  |
| 2A | CGAGGAGAGTA | 8.1 (7.4 - 8.8) | 10.9 (7.8 - 15.3) |  | *0.440* |
| 2B | CTCGGAGAGTT | 8.2 (7.5 - 8.9) | 9.5 (5.6 - 16.1) |  | *0.593* |
| 2C | CTCGGAGACTT | 8.6 (7.8 - 9.5) | 7.2 (6.0 - 8.7) | 6.6 (4.0 – 11.0) | *0.428* |
| 2D | CTCGGATAGTA | 8.4 (7.4 - 9.5) | 8.4 (7.3 - 9.7) | 7.0 (5.4 – 9.0) | *0.557* |
| 2E | TTATAAGAGAT | 8.2 (7.5 - 8.9) | 8.8 (5.4 - 14.2) | 12.8 (2.9 - 56.4) | *0.592* |
| 2F | TTATAAGACTT | 8.0 (7.3 - 8.8) | 10.2 (7.9 - 13.2) | 6.9 (2.5 - 18.8) | *0.500* |
| 2G | TTATAAGGGTT | 8.2 (7.5 - 8.9) | 9.2 (6.7 - 12.7) |  | *0.581* |
| 2H | TTCGGAGAGTA | 8.3 (7.6 - 9.2) | 7.5 (6.1 - 9.3) | 11.0 (4.9 - 24.8) | *0.600* |
|  |  |  |  |  |  |
| Block 3 |  |  |  |  |  |
| 3A | GCTTCCGG | 8.1 (7.3 - 8.9) | 8.8 (7.2 - 10.8) | 6.9 (1.7 - 28.6) | *0.588* |
| 3B | GCTCCCGA | 8.0 (7.3 - 8.8) | 9.8 (7.7 - 12.4) |  | *0.475* |
| 3C | GCTCTCGG | 8.5 (7.7 - 9.4) | 7.6 (6.3 - 9.1) | 6.7 (4.0 - 11.2) | *0.515* |
| 3D | GCTCTCGA | 8.6 (7.6 - 9.9) | 8.3 (7.3 - 9.3) | 6.4 (4.9 - 8.5) | *0.462* |
| 3E | GCTTCCAG | 8.4 (7.6 - 9.3) | 7.6 (6.4 - 9.1) | 8.2 (5.2 - 12.9) | *0.575* |
| 3F | GCCCCCGG | 8.3 (7.5 – 9.0) | 7.9 (5.4 - 11.5) |  | *0.608* |
| 3G | GCCCCCGA | 8.1 (7.3 - 8.9) | 8.2 (6.5 - 10.3) | 14.8 (8.3 - 26.3) | *0.504* |
| 3H | GTTCTCGA | 8.2 (7.5 - 8.9) | 9.0 (5.2 - 15.4) | 19.4 (4.7 - 79.1) | *0.553* |
| 3I | GCTCCTGG | 8.0 (7.3 - 8.8) | 10.4 (7.9 - 13.6) | 7.6 (3.3 - 17.3) | *0.500* |
|  |  |  |  |  |  |
| Block 4 |  |  |  |  |  |
| 4A | CTAGTTCA | 8.1 (7.2 - 9.1) | 8.3 (7.2 - 9.5) | 8.7 (6.7 - 11.3) | *0.595* |
| 4B | CTAATTGG | 8.1 (7.3 - 8.9) | 8.9 (7.1 - 11.1) | 9.4 (5.0 - 17.8) | *0.564* |
| 4C | CTAAGACG | 8.1 (7.4 - 8.9) | 8.7 (6.8 - 11.2) | 27.5 (6.7 - 113.5) | *0.555* |
| 4D | CTGGTTCA | 8.3 (7.5 - 9.2) | 8.1 (6.8 - 9.6) | 7.3 (4.6 - 11.8) | *0.595* |
| 4E | CCAAGACA | 8.0 (7.3 - 8.8) | 9.3 (7.5 - 11.6) | 8.5 (5.6 - 13.1) | *0.561* |
| 4F | TCAATTCG | 8.6 (7.8 - 9.4) | 6.9 (5.5 - 8.6) | 1.4 (0.3 - 5.7) | *0.295* |
| 4G | TCAATACG | 8.4 (7.6 - 9.2) | 7.5 (6.0 - 9.3) | 6.3 (2.8 - 14.2) | *0.534* |
|  |  |  |  |  |  |

^a^ Haplotypes with frequencies ≥ 5% in the multiethnic panel are shown.

^b^ Least square means of prolactin levels and 95% confidence interval; LS means adjusted for age, ethnicity, and assay batch.

^c^ p-value for test of heterogeneity or p-value for test of difference.

# Table S15. Associations between prolactin receptor (PRLR) haplotypes and prolactin levels

|  |  | LS means^b^ (95% CI) | | |  |
| --- | --- | --- | --- | --- | --- |
|  | Haplotype^a^ | None | One copy | Two copies | P^c^ |
| Block 1 |  |  |  |  |  |
| 1A | GTAGGG | 8.3 (7.5 - 9.1) | 7.7 (6.0 - 9.8) | 10.5 (4.6 - 24.1) | *0.609* |
| 1B | GTAAGT | 8.4 (7.5 - 9.4) | 8.2 (7.0 - 9.5) | 7.1 (5.2 - 9.8) | *0.567* |
| 1C | GTAGGT | 8.2 (7.5 – 9.0) | 8.4 (5.9 - 12) | 8.6 (3.0 - 24.4) | *0.610* |
| 1D | GTGAGT | 8.6 (7.6 - 9.6) | 7.6 (6.4 - 8.9) | 8.4 (6.3 - 11.1) | *0.597* |
| 1E | GCAGGT | 8.0 (7.2 - 8.8) | 9.2 (7.4 - 11.4) | 10.8 (6.8 - 17.2) | *0.457* |
| 1F | ATAAGG | 8.2 (7.3 - 9.1) | 7.9 (6.8 - 9.2) | 11.0 (7.7 - 15.7) | *0.573* |
|  |  |  |  |  |  |
| Block 2 |  |  |  |  |  |
| 2A | GTACA | 8.7 (7.5 - 10.2) | 8.4 (7.4 - 9.5) | 7.2 (5.9 - 8.7) | *0.486* |
| 2B | GTGCA | 8.0 (7.3 - 8.8) | 9.7 (7.2 - 13.1) | 20.3 (7.4 - 55.4) | *0.430* |
| 2C | GTGTA | 8.2 (7.4 – 9.0) | 8.3 (6.8 – 10.0) | 11.1 (6.2 – 20.0) | *0.588* |
| 2D | GTGTG | 8.4 (7.5 - 9.4) | 7.8 (6.5 - 9.3) | 8.3 (6.3 – 11.0) | *0.606* |
| 2E | GAGCA | 8.1 (7.4 - 8.9) | 9.3 (7.1 - 12.4) | 10.4 (5.4 - 19.8) | *0.533* |
| 2F | CTGCA | 8.3 (7.5 – 9.0) | 8.9 (6.5 - 12.3) | 3.8 (1.4 - 10.4) | *0.602* |
|  |  |  |  |  |  |
| Block 3 |  |  |  |  |  |
| 3A | CCTTAGTAC | 8.3 (7.2 - 9.5) | 7.8 (6.9 - 8.9) | 9.1 (7.3 - 11.4) | *0.605* |
| 3B | CCTTAGTAG | 8.2 (7.5 – 9.0) | 9.2 (5.7 - 14.9) | 5.3 (1.3 - 21.3) | *0.611* |
| 3C | CCTTGACAC | 8.4 (7.6 - 9.2) | 7.6 (5.6 - 10.3) | 3.7 (1.3 - 10.1) | *0.517* |
| 3D | CCTTGACGC | 8.1 (7.5 - 8.9) | 11.5 (6.9 - 19.1) |  | *0.511* |
| 3E | CCTTGACGG | 8.2 (7.4 – 9.0) | 8.7 (6.7 - 11.2) | 6.7 (2.4 - 18.4) | *0.609* |
| 3F | CTACGACGC | 8.4 (7.6 - 9.2) | 7.5 (6.0 - 9.3) | 8.0 (4.2 - 15.1) | *0.574* |
| 3G | GCACGATAC | 7.5 (6.6 - 8.5) | 9.1 (7.8 - 10.7) | 9.6 (7.3 - 12.7) | *0.426* |
|  |  |  |  |  |  |
| Block 4 |  |  |  |  |  |
| 4A | CTTGGGC | 8.2 (7.5 - 9.0) | 8.2 (6.5 - 10.2) | 17.4 (6.4 - 47.1) | *0.595* |
| 4B | CTAGGGC | 7.6 (6.6 - 8.7) | 8.2 (7.2 - 9.5) | 9.7 (8 - 11.7) | *0.409* |
| 4C | CTAGAGC | 8.4 (7.6 - 9.4) | 7.8 (6.5 - 9.2) | 8.3 (5.7 - 12) | *0.596* |
| 4D | TTAGGGT | 8.5 (7.7 - 9.3) | 6.6 (5.1 - 8.7) |  | *0.447* |
| 4E | TTAGGAT | 8.4 (7.6 - 9.2) | 7.7 (6.2 - 9.5) | 7.3 (3.6 - 14.7) | *0.573* |
| 4F | TTACGGT | 8.2 (7.5 - 9.0) | 7.5 (5.7 - 9.9) | 14.9 (7.3 - 30.3) | *0.600* |
| 4G | TCACGGT | 8.3 (7.6 - 9.1) | 7.8 (6.0 - 10.2) | 5.6 (2.8 - 11.3) | *0.558* |
|  |  |  |  |  |  |
| Block 5 |  |  |  |  |  |
| 5A | CCTCAT | 11.6 (8.0 - 16.7) | 8.2 (7.0 - 9.6) | 8.0 (7.2 - 8.9) | *0.499* |
| 5B | CCTTAT | 8.2 (7.6 - 9.0) | 7.7 (4.5 - 13.5) |  | *0.608* |
| 5C | CGCCGT | 8.2 (7.4 - 8.9) | 8.7 (6.8 - 11.2) | 11.6 (4.3 - 31.6) | *0.582* |
| 5D | TGTCGT | 8.0 (7.3 - 8.7) | 13.9 (9.2 - 21.1) | 11.8 (4.4 - 32.1) | *0.305* |
| 5E | TGTTAT | 8.1 (7.4 - 8.9) | 9.7 (7.3 - 13.1) | 1.8 (0.5 - 7.4) | *0.608* |
|  |  |  |  |  |  |
| Block 6 |  |  |  |  |  |
| 6A | TTAAA | 8.4 (7.4 - 9.4) | 8.4 (7.2 - 9.8) | 6.7 (4.9 - 9.1) | *0.559* |
| 6B | TTGAA | 7.9 (6.7 - 9.5) | 8.1 (7.2 - 9.2) | 8.7 (7.3 - 10.3) | *0.582* |
| 6C | TCGAG | 8 (7.2 - 8.7) | 10.3 (8.2 - 13.1) | 6.0 (2.7 - 13.5) | *0.503* |
| 6D | TCGGG | 8.2 (7.5 – 9.0) | 8.5 (4.5 - 16.2) |  | *0.611* |
| 6E | CCGAG | 8.3 (7.6 - 9.1) | 7.6 (5.4 - 10.8) | 8.6 (2.1 - 35.3) | *0.604* |
| 6F | CCGGG | 8.2 (7.5 – 9.0) | 8.4 (6.7 - 10.5) | 10.2 (4.5 - 23.4) | *0.604* |
|  |  |  |  |  |  |
| Block 7 |  |  |  |  |  |
| 7A | CGCTATCCT | 8.8 (6.9 - 11.2) | 8.3 (7.3 - 9.4) | 8.0 (7.0 - 9.2) | *0.588* |
| 7B | CGCTAGCCA | 8.1 (7.4 - 8.8) | 9.8 (7.5 - 12.9) | 15.7 (3.8 - 64.2) | *0.467* |
|  | CGCTGGCAT | 8.2 (7.5 - 8.9) | 11.6 (6.1 - 22.2) | 11.3 (2.8 - 46.1) | *0.544* |
| 7C | CGCCATCCT | 8.3 (7.6 – 9.0) | 7.6 (4.3 - 13.6) | 1.8 (0.4 - 7.4) | *0.469* |
| 7D | TGTTAGCCT | 8.2 (7.5 - 8.9) | 11.2 (6.6 – 19.0) |  | *0.529* |
| 7E | TGTTAGCCA | 8.3 (7.5 - 9.1) | 8.0 (6.8 - 9.4) | 13.4 (7.0 - 25.9) | *0.606* |
|  |  |  |  |  |  |
| Block 8 |  |  |  |  |  |
| 8A | CGTG | 9.0 (6.9 - 11.7) | 8.2 (7.1 - 9.4) | 8.1 (7.2 - 9.2) | *0.595* |
| 8B | CATG | 8.1 (7.4 - 8.9) | 9.4 (6.3 - 14.2) | 12.1 (4.4 - 33.2) | *0.556* |
| 8C | TGCG | 8.1 (7.4 - 8.9) | 9.2 (6.7 - 12.8) | 15.5 (3.8 - 63.7) | *0.549* |
| 8D | TGCA | 8.5 (7.6 - 9.4) | 7.4 (6.3 - 8.7) | 10.0 (6.8 - 14.8) | *0.603* |
| 8E | TATG | 8.2 (7.5 - 8.9) | 10.4 (6.5 - 16.7) |  | *0.555* |
|  |  |  |  |  |  |
| Block 9 |  |  |  |  |  |
| 9A | ATGTT | 8.0 (7.0 - 9.1) | 8.4 (7.3 - 9.7) | 8.7 (6.8 - 11.1) | *0.587* |
| 9B | ATGTC | 8.6 (7.6 - 9.7) | 7.8 (6.7 - 9.2) | 7.7 (5.8 - 10.1) | *0.563* |
| 9C | ATGCT | 8.3 (7.5 – 9.0) | 7.1 (4.9 - 10.2) | 27.5 (6.6 - 114.8) | *0.611* |
| 9D | ATATT | 8.3 (7.5 - 9.1) | 8.0 (6.2 - 10.2) | 7.7 (1.9 - 32.1) | *0.606* |
| 9E | GCGTT | 8.1 (7.2 - 9.1) | 8.3 (7.3 - 9.5) | 8.4 (6.4 - 11.1) | *0.604* |
|  |  |  |  |  |  |

^a^ Haplotypes with frequencies ≥ 5% in the multiethnic panel are shown.

^b^ Least square means of prolactin levels and 95% confidence interval; LS means adjusted for age, ethnicity, and assay batch.

^c^ p-value for test of heterogeneity or test of difference.
