# Supplementary figures and images for: A comprehensive analysis of common genetic variation in prolactin (PRL) and PRL receptor (PRLR) genes in relation to plasma prolactin levels and breast cancer risk: the Multiethnic Cohort
Source: BMC Med Genet. 2007 Dec 1;8:72. doi: 10.1186/1471-2350-8-72 (PMC2219987; doi:10.1186/1471-2350-8-72)

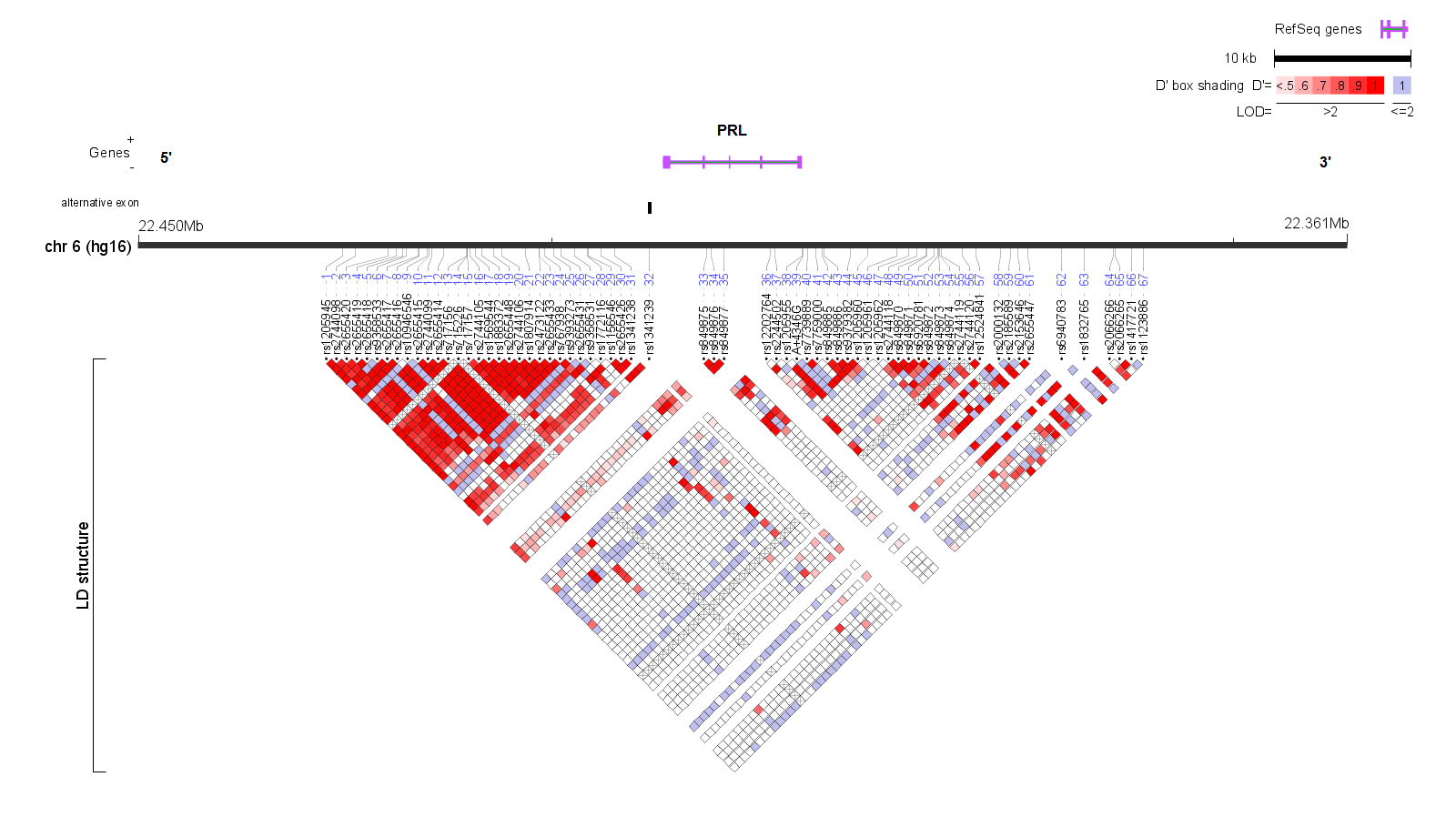

Supplement: Additional file 2 — Linkage disequilibrium (LD) plot across the prolactin (PRL) locus for African-Americans. The horizontal black line depicts the 59-kilobase region of chromosome (chr) 6 analyzed in our multiethnic panel. The PRL gene is shown in grey (RefSeq gene = completed genes from the human genome assembly). Alternative exon 1a (associated with the distal extra-pituitary promoter region) lies 5.8 kb upstream of exon 1 (associated with the pituitary promoter region). The LD plot, presented at the bottom of the figures, is based on the measure of D'. Each diamond indicates the pairwise magnitude of LD, with dark grey indicating strong LD (D' > 0.8) and a logarithm of odds score of greater than 2.0. Single nucleotide polymorphisms (SNPs) < 5% for this ethnic group not shown. (Figure prepared with LocusView, Broad Institute, Cambridge, MA, unpublished software by T. Petryshen, A. Kirby, and M. Ainscow [61]). [file 1471-2350-8-72-S2.png]

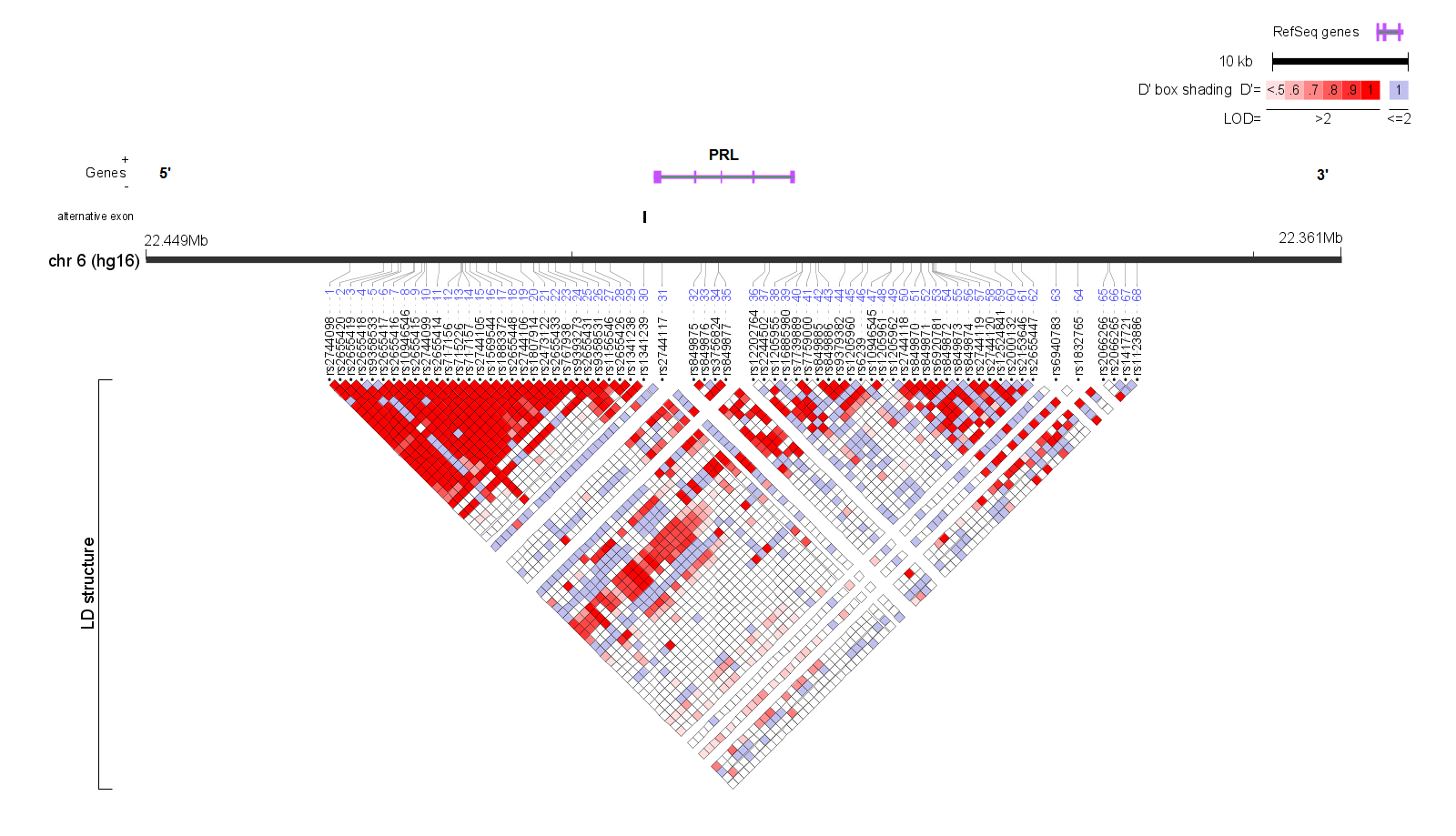

Supplement: Additional file 3 — Linkage disequilibrium (LD) plot across the prolactin (PRL) locus for Native-Hawaiians. The horizontal black line depicts the 59-kilobase region of chromosome (chr) 6 analyzed in our multiethnic panel. The PRL gene is shown in grey (RefSeq gene = completed genes from the human genome assembly). Alternative exon 1a (associated with the distal extra-pituitary promoter region) lies 5.8 kb upstream of exon 1 (associated with the pituitary promoter region). The LD plot, presented at the bottom of the figures, is based on the measure of D'. Each diamond indicates the pairwise magnitude of LD, with dark grey indicating strong LD (D' > 0.8) and a logarithm of odds score of greater than 2.0. Single nucleotide polymorphisms (SNPs) < 5% for this ethnic group not shown. (Figure prepared with LocusView, Broad Institute, Cambridge, MA, unpublished software by T. Petryshen, A. Kirby, and M. Ainscow [61]). [file 1471-2350-8-72-S3.png]

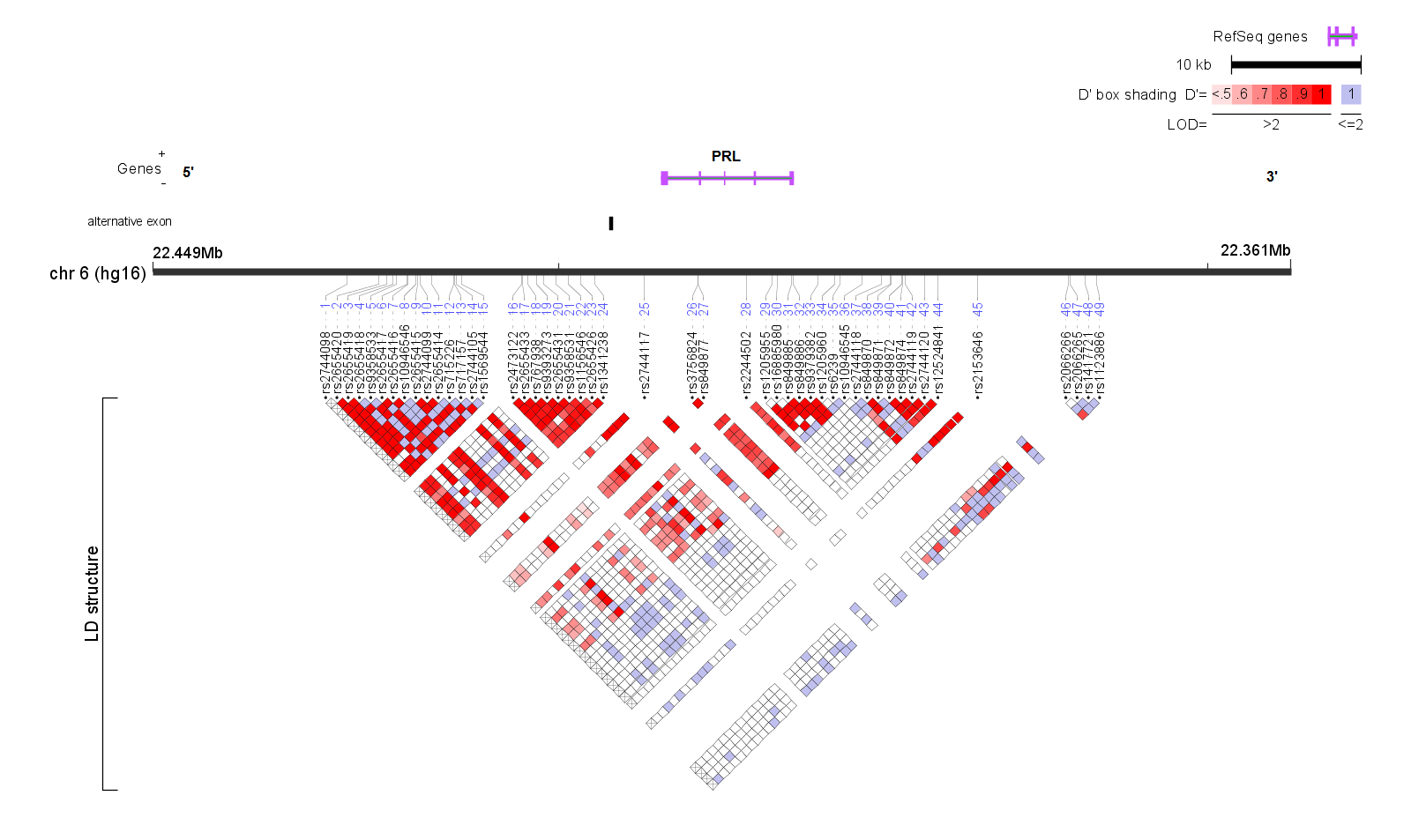

Supplement: Additional file 4 — Linkage disequilibrium (LD) plot across the prolactin (PRL) locus for Japanese-Americans. The horizontal black line depicts the 59-kilobase region of chromosome (chr) 6 analyzed in our multiethnic panel. The PRL gene is shown in grey (RefSeq gene = completed genes from the human genome assembly). Alternative exon 1a (associated with the distal extra-pituitary promoter region) lies 5.8 kb upstream of exon 1 (associated with the pituitary promoter region). The LD plot, presented at the bottom of the figures, is based on the measure of D'. Each diamond indicates the pairwise magnitude of LD, with dark grey indicating strong LD (D' > 0.8) and a logarithm of odds score of greater than 2.0. Single nucleotide polymorphisms (SNPs) < 5% for this ethnic group not shown. (Figure prepared with LocusView, Broad Institute, Cambridge, MA, unpublished software by T. Petryshen, A. Kirby, and M. Ainscow [61]). [file 1471-2350-8-72-S4.png]

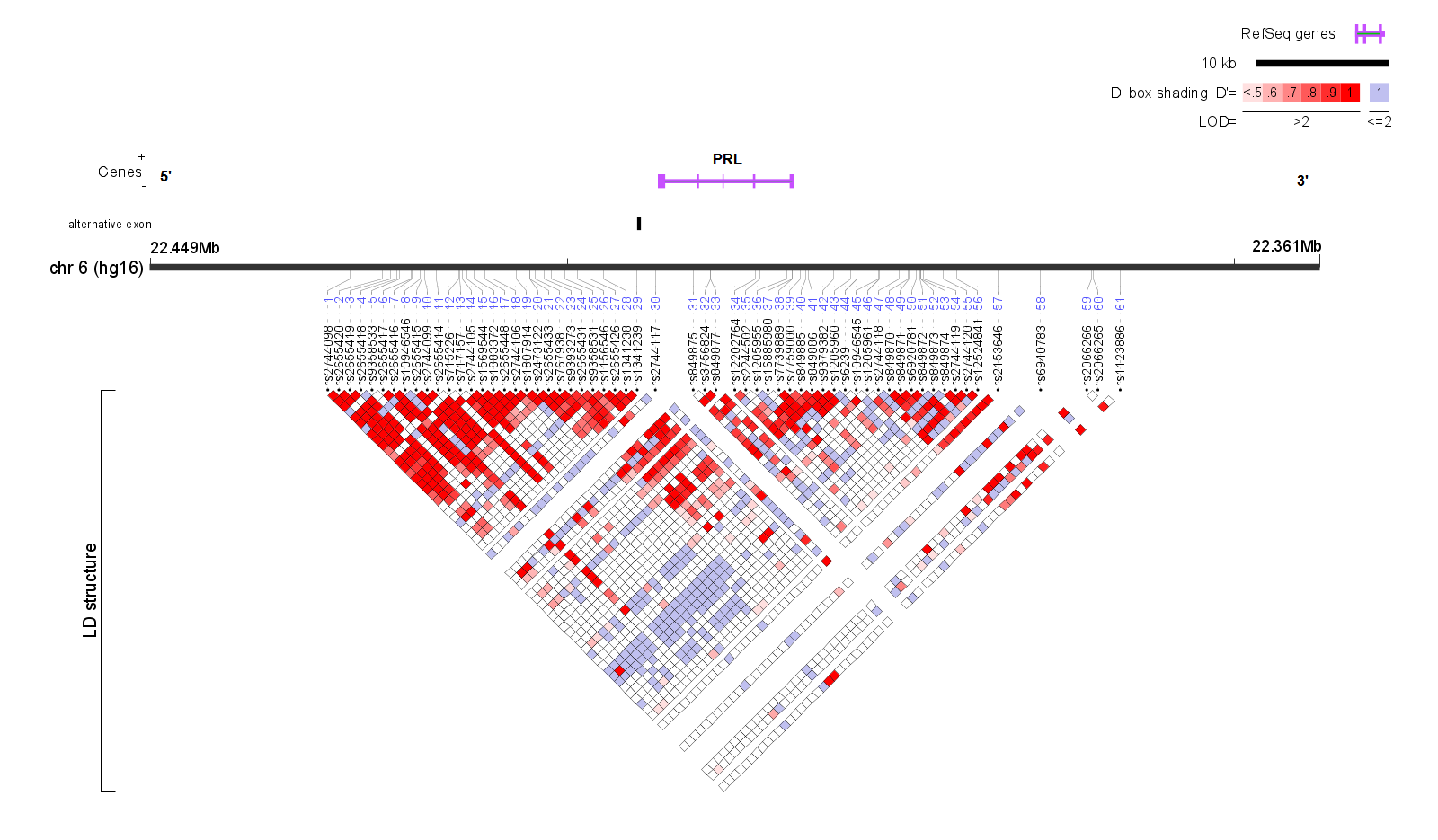

Supplement: Additional file 5 — Linkage disequilibrium (LD) plot across the prolactin (PRL) locus for Latinas. The horizontal black line depicts the 59-kilobase region of chromosome (chr) 6 analyzed in our multiethnic panel. The PRL gene is shown in grey (RefSeq gene = completed genes from the human genome assembly). Alternative exon 1a (associated with the distal extra-pituitary promoter region) lies 5.8 kb upstream of exon 1 (associated with the pituitary promoter region). The LD plot, presented at the bottom of the figures, is based on the measure of D'. Each diamond indicates the pairwise magnitude of LD, with dark grey indicating strong LD (D' > 0.8) and a logarithm of odds score of greater than 2.0. Single nucleotide polymorphisms (SNPs) < 5% for this ethnic group not shown. (Figure prepared with LocusView, Broad Institute, Cambridge, MA, unpublished software by T. Petryshen, A. Kirby, and M. Ainscow [61]). [file 1471-2350-8-72-S5.png]

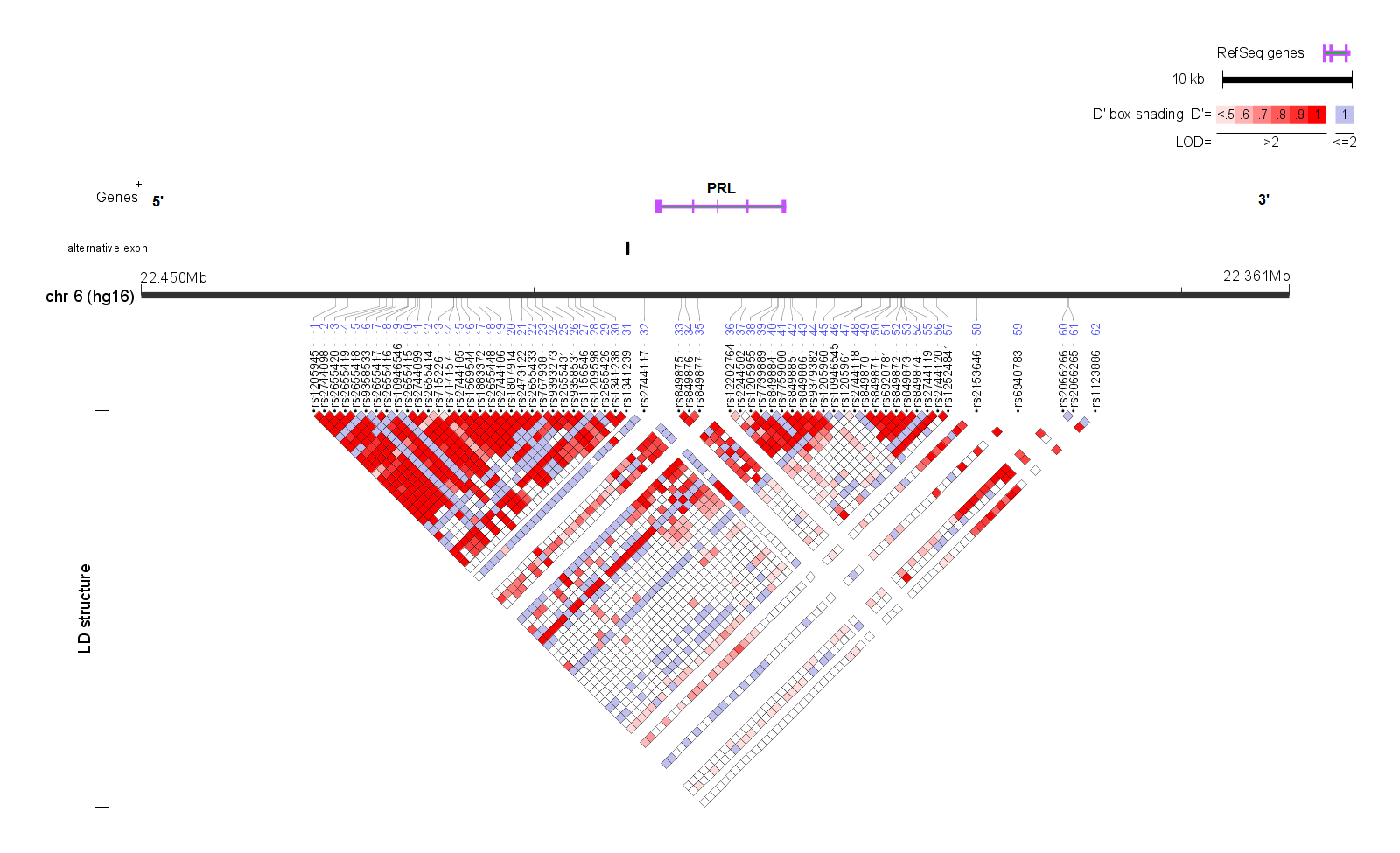

Supplement: Additional file 6 — Linkage disequilibrium (LD) plot across the prolactin (PRL) locus for Whites. The horizontal black line depicts the 59-kilobase region of chromosome (chr) 6 analyzed in our multiethnic panel. The PRL gene is shown in grey (RefSeq gene = completed genes from the human genome assembly). Alternative exon 1a (associated with the distal extra-pituitary promoter region) lies 5.8 kb upstream of exon 1 (associated with the pituitary promoter region). The LD plot, presented at the bottom of the figures, is based on the measure of D'. Each diamond indicates the pairwise magnitude of LD, with dark grey indicating strong LD (D' > 0.8) and a logarithm of odds score of greater than 2.0. Single nucleotide polymorphisms (SNPs) < 5% for this ethnic group not shown. (Figure prepared with LocusView, Broad Institute, Cambridge, MA, unpublished software by T. Petryshen, A. Kirby, and M. Ainscow [61]). [file 1471-2350-8-72-S6.png]

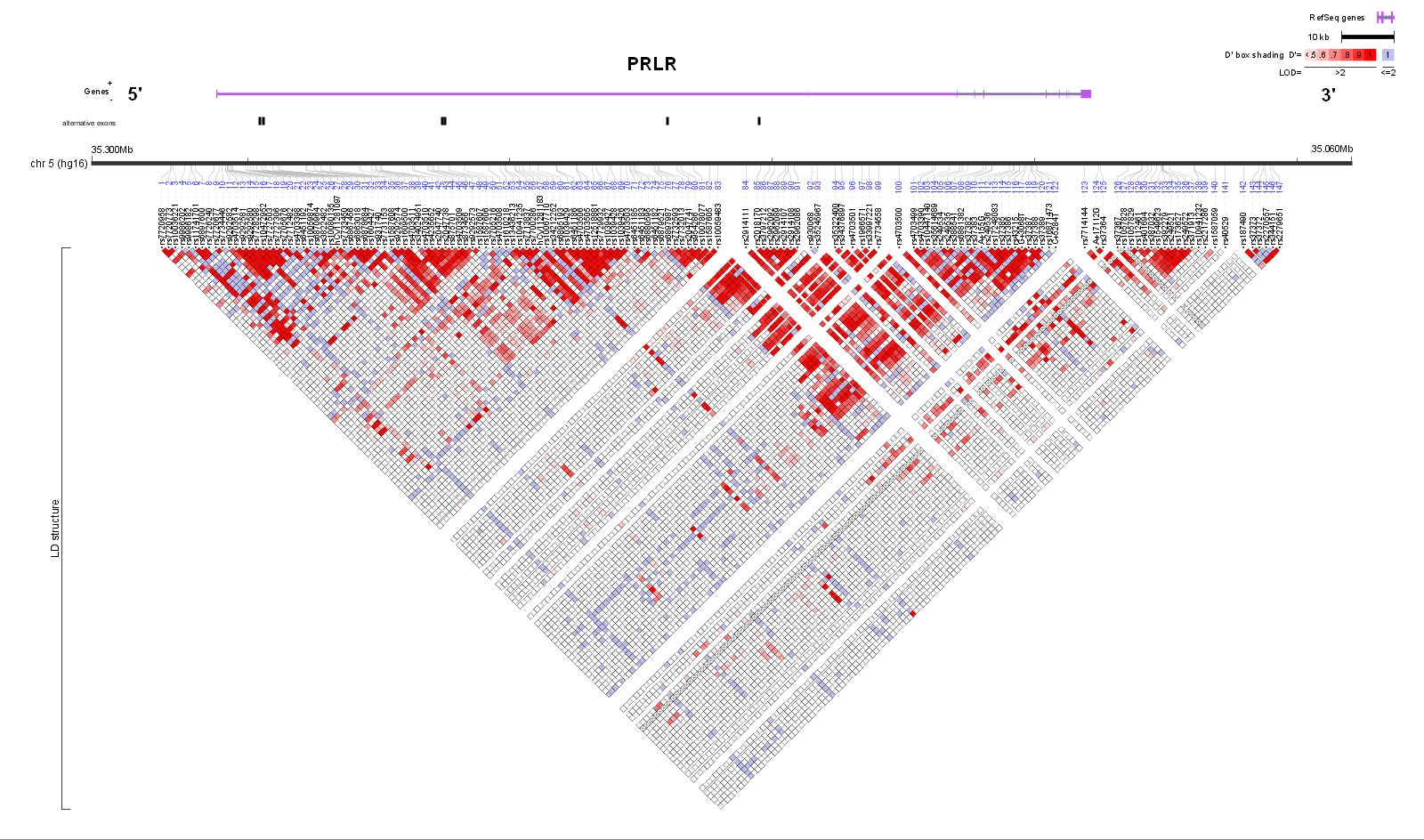

Supplement: Additional file 7 — Linkage disequilibrium (LD) plot across the prolactin receptor (PRLR) locus for African-Americans. The horizontal black line depicts the 210-kilobase region of chromosome (chr) 5 analyzed in our multiethnic panel. The PRLR gene is shown in grey (RefSeq gene = completed genes from the human genome assembly). Alternative first exons are shown in black below the gene: hE13, hE1N1, hE1N2, hE1N3, hE1N4, and hE1N5. The LD plot, presented at the bottom of the figures, is based on the measure of D'. Each diamond indicates the pairwise magnitude of LD, with dark grey indicating strong LD (D' > 0.8) and a logarithm of odds score of greater than 2.0. Single nucleotide polymorphisms (SNPs) < 5% for this ethnic group not shown. (Figure prepared with LocusView, Broad Institute, Cambridge, MA, unpublished software by T. Petryshen, A. Kirby, and M. Ainscow [61]). [file 1471-2350-8-72-S7.png]

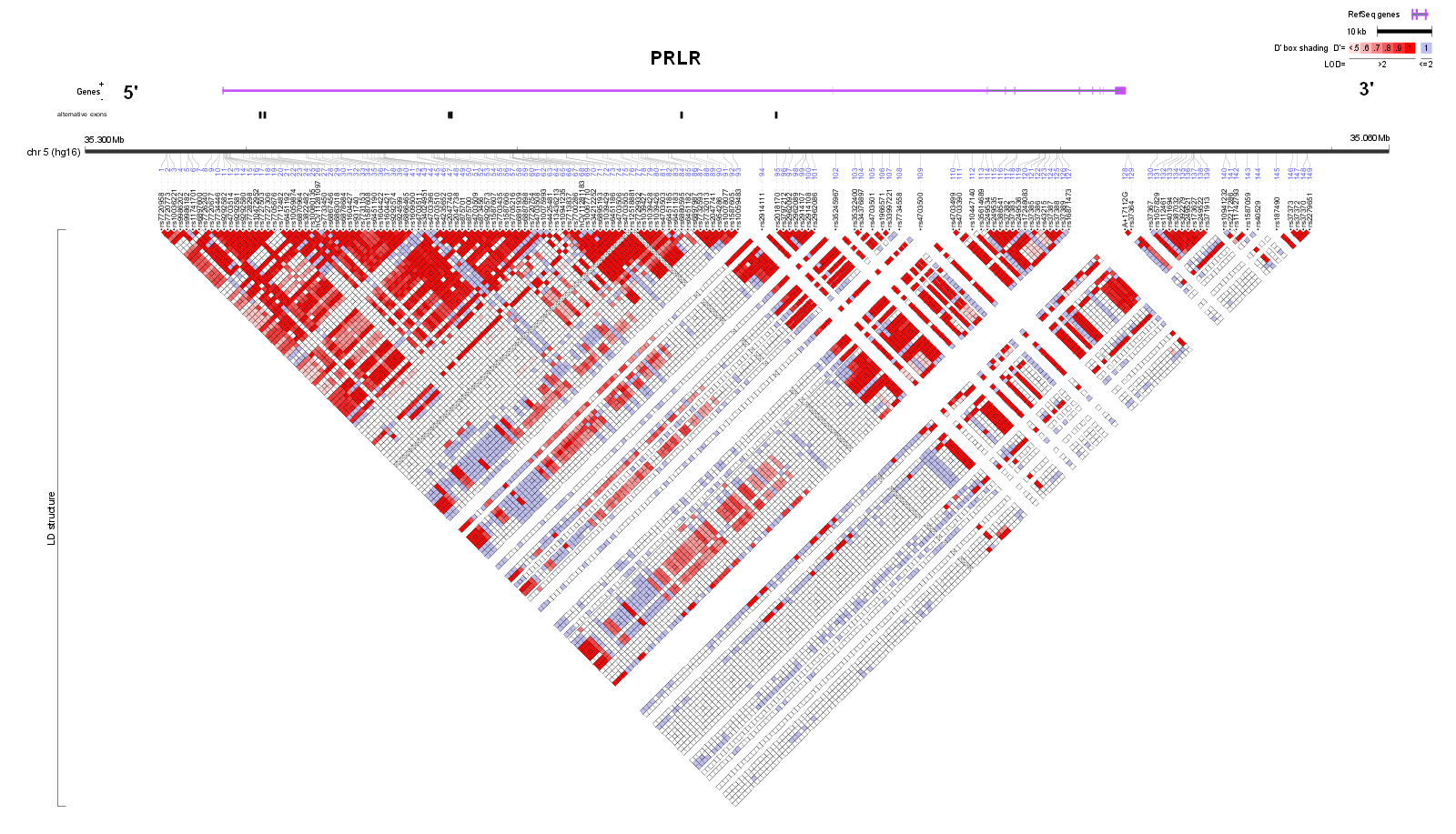

Supplement: Additional file 8 — Linkage disequilibrium (LD) plot across the prolactin receptor (PRLR) locus for Native-Hawaiians. The horizontal black line depicts the 210-kilobase region of chromosome (chr) 5 analyzed in our multiethnic panel. The PRLR gene is shown in grey (RefSeq gene = completed genes from the human genome assembly). Alternative first exons are shown in black below the gene: hE13, hE1N1, hE1N2, hE1N3, hE1N4, and hE1N5. The LD plot, presented at the bottom of the figures, is based on the measure of D'. Each diamond indicates the pairwise magnitude of LD, with dark grey indicating strong LD (D' > 0.8) and a logarithm of odds score of greater than 2.0. Single nucleotide polymorphisms (SNPs) < 5% for this ethnic group not shown. (Figure prepared with LocusView, Broad Institute, Cambridge, MA, unpublished software by T. Petryshen, A. Kirby, and M. Ainscow [61]). [file 1471-2350-8-72-S8.png]

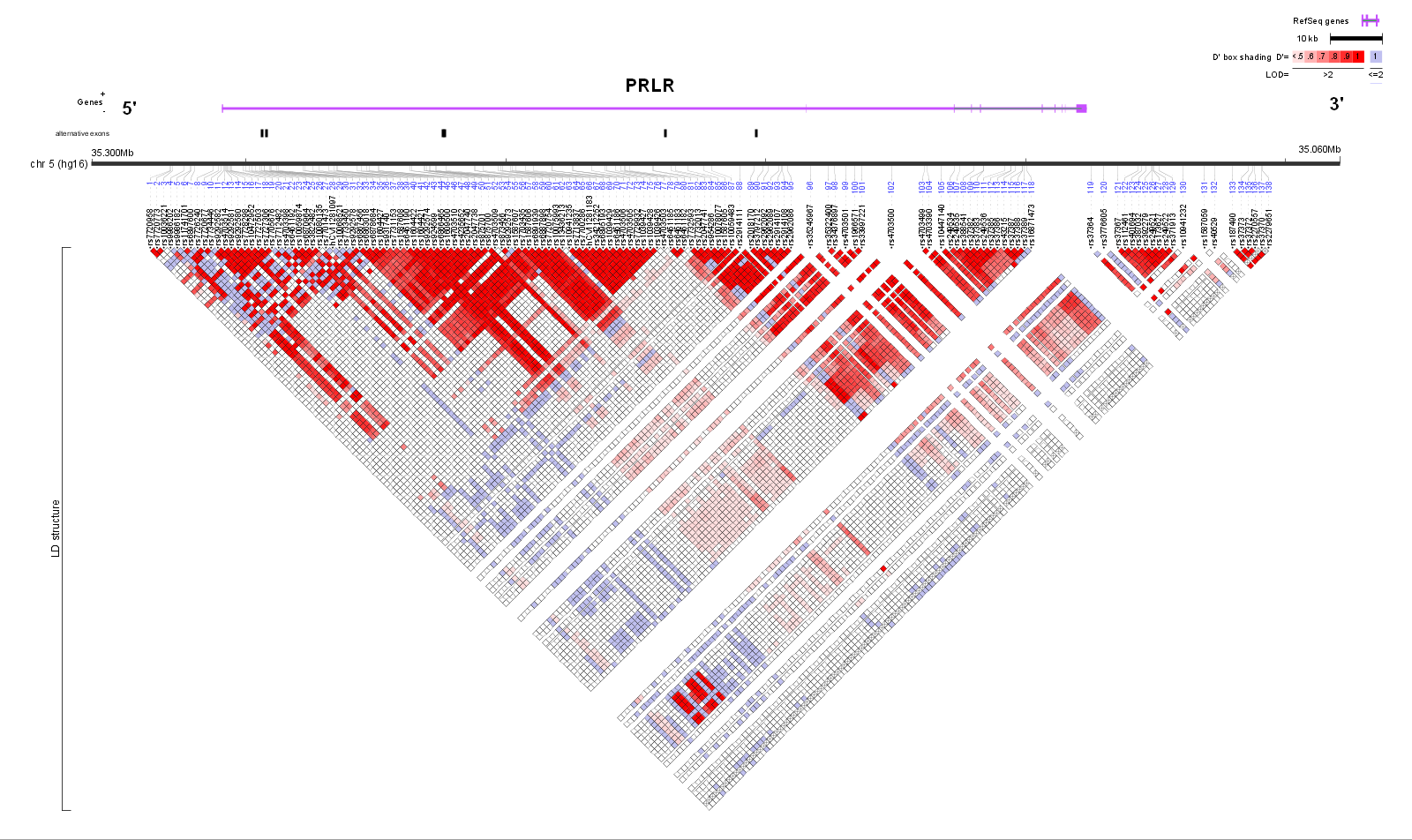

Supplement: Additional file 9 — Linkage disequilibrium (LD) plot across the prolactin receptor (PRLR) locus for Japanese-Americans. The horizontal black line depicts the 210-kilobase region of chromosome (chr) 5 analyzed in our multiethnic panel. The PRLR gene is shown in grey (RefSeq gene = completed genes from the human genome assembly). Alternative first exons are shown in black below the gene: hE13, hE1N1, hE1N2, hE1N3, hE1N4, and hE1N5. The LD plot, presented at the bottom of the figures, is based on the measure of D'. Each diamond indicates the pairwise magnitude of LD, with dark grey indicating strong LD (D' > 0.8) and a logarithm of odds score of greater than 2.0. Single nucleotide polymorphisms (SNPs) < 5% for this ethnic group not shown. (Figure prepared with LocusView, Broad Institute, Cambridge, MA, unpublished software by T. Petryshen, A. Kirby, and M. Ainscow [61]). [file 1471-2350-8-72-S9.png]

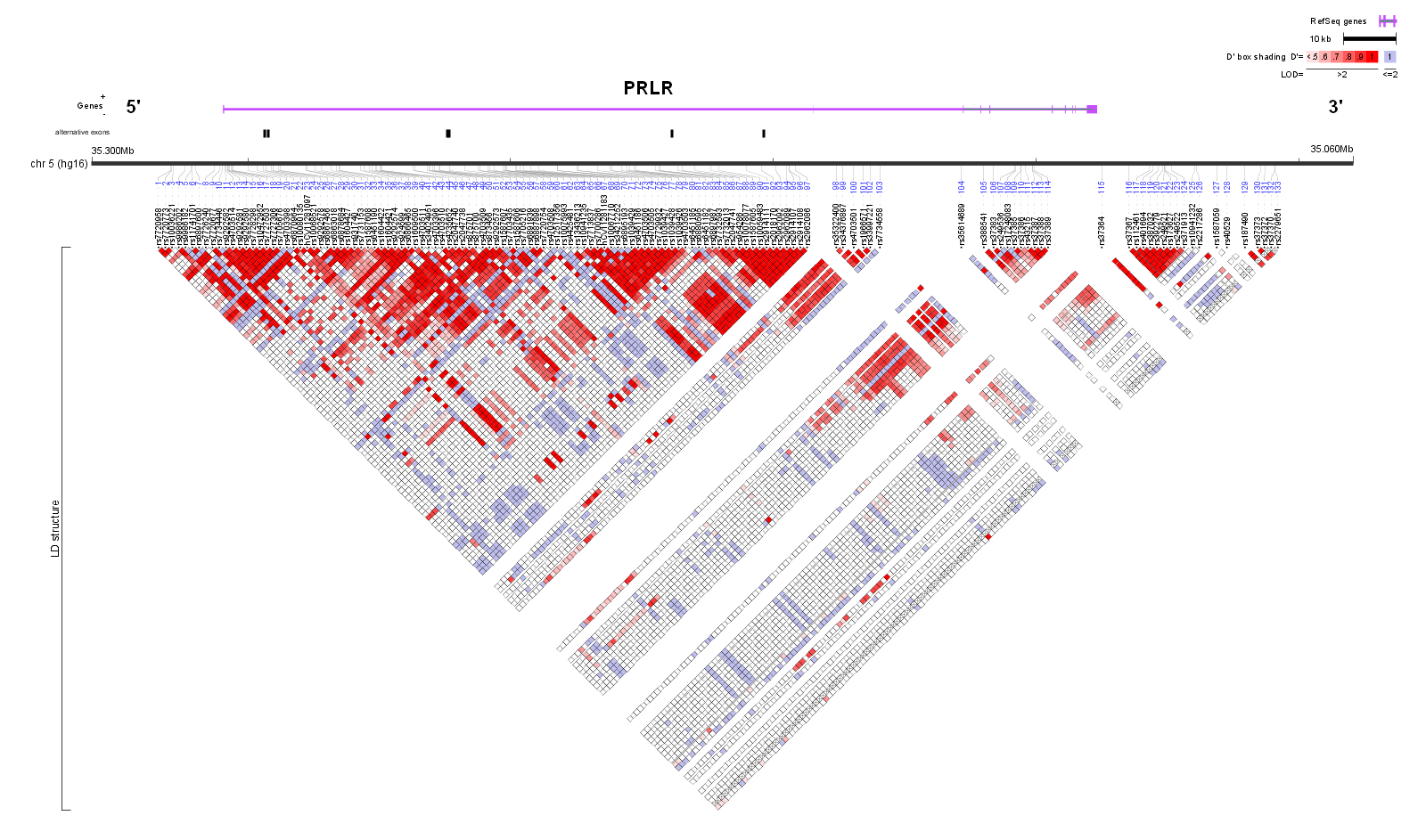

Supplement: Additional file 10 — Linkage disequilibrium (LD) plot across the prolactin receptor (PRLR) locus for Latinas. The horizontal black line depicts the 210-kilobase region of chromosome (chr) 5 analyzed in our multiethnic panel. The PRLR gene is shown in grey (RefSeq gene = completed genes from the human genome assembly). Alternative first exons are shown in black below the gene: hE13, hE1N1, hE1N2, hE1N3, hE1N4, and hE1N5. The LD plot, presented at the bottom of the figures, is based on the measure of D'. Each diamond indicates the pairwise magnitude of LD, with dark grey indicating strong LD (D' > 0.8) and a logarithm of odds score of greater than 2.0. Single nucleotide polymorphisms (SNPs) < 5% for this ethnic group not shown. (Figure prepared with LocusView, Broad Institute, Cambridge, MA, unpublished software by T. Petryshen, A. Kirby, and M. Ainscow [61]). [file 1471-2350-8-72-S10.png]

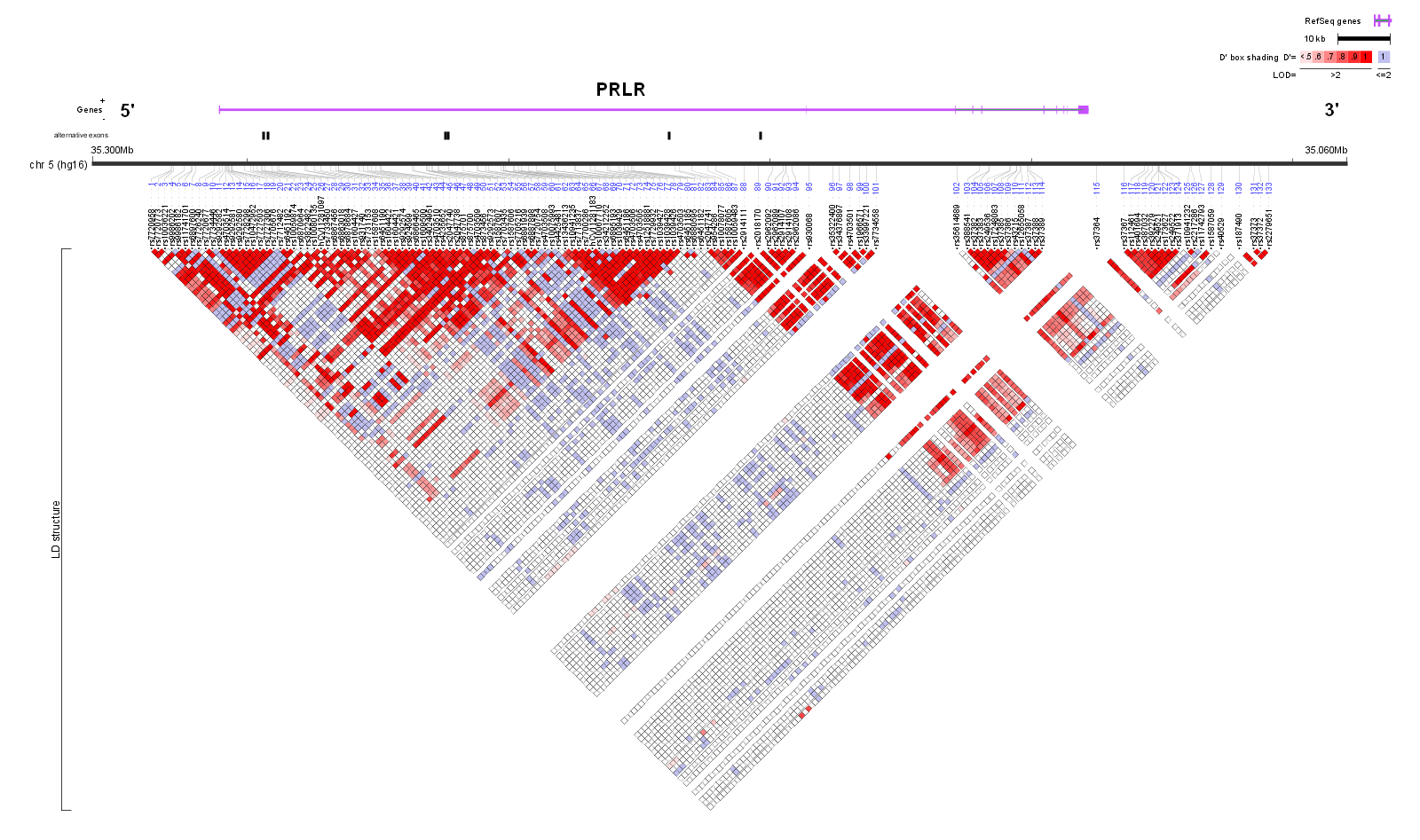

Supplement: Additional file 11 — Linkage disequilibrium (LD) plot across the prolactin receptor (PRLR) locus for Whites. The horizontal black line depicts the 210-kilobase region of chromosome (chr) 5 analyzed in our multiethnic panel. The PRLR gene is shown in grey (RefSeq gene = completed genes from the human genome assembly). Alternative first exons are shown in black below the gene: hE13, hE1N1, hE1N2, hE1N3, hE1N4, and hE1N5. The LD plot, presented at the bottom of the figures, is based on the measure of D'. Each diamond indicates the pairwise magnitude of LD, with dark grey indicating strong LD (D' > 0.8) and a logarithm of odds score of greater than 2.0. Single nucleotide polymorphisms (SNPs) < 5% for this ethnic group not shown. (Figure prepared with LocusView, Broad Institute, Cambridge, MA, unpublished software by T. Petryshen, A. Kirby, and M. Ainscow [61]). [file 1471-2350-8-72-S11.png]
